# A review of Cunaxidae (Acariformes, Trombidiformes): Histories and diagnoses of subfamilies and genera, keys to world species, and some new locality records

**DOI:** 10.3897/zookeys.418.7629

**Published:** 2014-06-20

**Authors:** Michael J. Skvarla, J. Ray Fisher, Ashley P. G. Dowling

**Affiliations:** 1 Department of Entomology, 319 AGRI Building, University of Arkansas, Fayetteville, Arkansas, 72701, USA

**Keywords:** Identification, key, Bdelloidea, Prostigmata, Eupodina

## Abstract

Cunaxidae are predaceous mites found in a variety of habitats. This work provides comprehensive keys to world subfamilies, genera, and species. Diagnoses and historical reviews are provided for subfamilies and genera.

*Cunaxa boneti*, *C. denmarki*, *C. exoterica*, *C. floridanus*, *C. lehmanae*, *C. lukoschusi*, *C. metzi*, *C. myabunderensis*, *C newyorkensis*, *C. rackae*, *C. reevesi*, and *C. reticulatus* are moved to *Rubroscirus* and *C. otiosus*,﻿ *C. valentis*, and *C. rasile* are returned to *Rubroscirus*. *Cunaxoides neopectinatus* is moved to *Pulaeus*. *Neocunaxoides pradhani* and *N. gilbertoi* are transferred to *Scutopalus*. *Pulaeus minutus* and *P. subterraneus* are moved to *Lupaeus*. *Pseudobonzia bakari*, *P. malookensis*, and *P. shamshadi* are transferred to *Neobonzia. Dactyloscirus bifidus* is transferred to *Armascirus*.

*Scirula papillata* is reported from the Western Hemisphere for the first time. *Armascirus ozarkensis*, *A. primigenius*, and *Dactyloscirus dolichosetosus* are reported from new localities.

## Introduction

**Figure 1.**
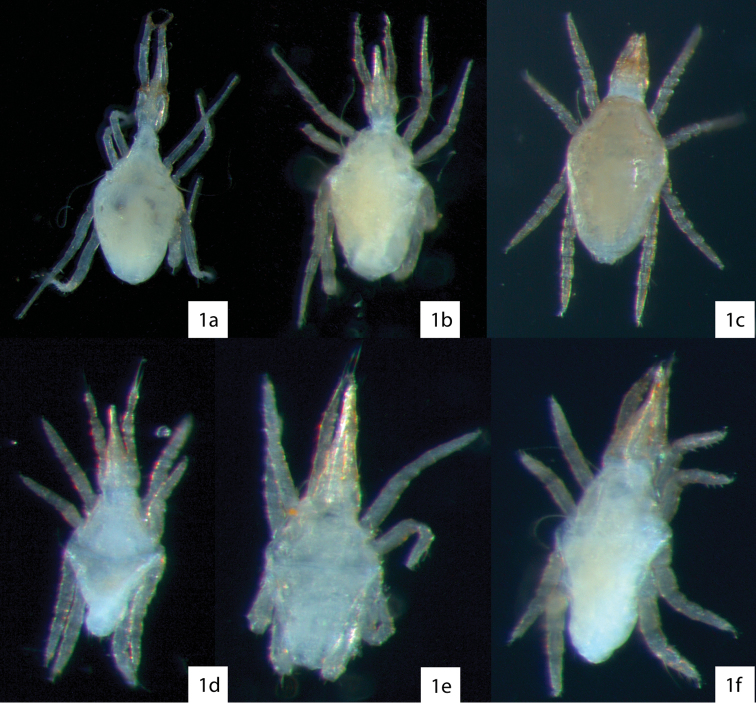
Examples of cunaxids in ethanol illustrating how they would appear while sorting. **1a** *Armascirus* **1b** *Cunaxa* **1c** *Pulaeus* **1d** *Parabonzia* **1e** *Coleoscirus* **1f** *Neobonzia*.

Cunaxidae (Fig. 1) are common predatory mites that are present in forest systems, grasslands, agricultural fields, and anthropogenically disturbed areas. Surveys of mites in these habitats often report only family or generic-level identification. This is problematic because little is known about where cunaxid species occur, both regionally and in what habitats, and unfortunate because such reports are potentially very useful collectively if species were identified.

Part of the reason behind the lack of specific identification is the difficulty in reliably identifying cunaxids without extensive knowledge of the primary literature. Keys to cunaxid species are often regional, so of little use to researchers outside of that specific region, and scattered across countless journals. The last comprehensive attempt to present keys to world species was by Smiley (1992). The number of described species since Smiley published his monograph has more than doubled (166 to 400+). Updated keys reflecting known diversity and current taxonomic opinion are therefore imperative if researchers are to identify individuals to the specific rather than generic or family level.

**Biology.** All cunaxids are thought to be opportunistic predators, though an undescribed *Rubroscirus* was observed to drink drops of honeydew in addition to feeding on live prey (Walter and Proctor 1999). Cunaxids have been reported to feed on active prey such as Collembola (Sellnick 1926, Heryford 1965), bark lice (Zaher et al. 1975a), and thrips (Milne 1977), and relatively inactive prey such as scales (Ewing and Webster 1912, Gerson 1971), nematodes (Taha et al. 1988, Walter and Kaplan1991), phytophagous mites (Meyer and Ryke 1959, Zaher et al. 1975a, Den Heyer and Ryke 1970, Taha et al. 1988, Smiley 1992, Sathiamma 1995, Arbabi and Singh 2000, Ferla 2001, Lahiri et al. 2004, Castro and Moraes 2010), and paratydeid mites (pers. obs.). They fail to survive when offered only plant material (Zaher et al. 1975a).

Both ambush and active hunting have evolved within the family, sometimes within the same subfamily. Within Cunaxinae, for instance, *Armascirus* and *Dactyloscirus* wait, sometimes for hours, to ambush prey (Walter and Proctor 1999), whereas *Allocunaxa* actively search for prey (Castro and Moraes 2010).

Cunaxids occur in most terrestrial habitats, including soil and leaf litter (Den Heyer 1977a, Luxton 1982; Javan et al. 2012); moss and lichen (Sepasgosarian 1984, Tseng 1980); on vegetation (Miller 1925, Swift and Goff 2001, Ferla and Moraes 2002) including coniferous trees (Lehman 1982), tropical trees (Castro and Moraes 2007) including guava trees (Mallikarjunappa and Nageshchandra 1990), Ferla and Moraes 2002), mango trees (Mohamed et al. 2014), coconut trees (Mariau and Biggins 2001; da Silva et al. 2014), and rubber trees (Hernandes and Feres 2006), ornamental plants (Tagore and Putatunda 2003), invasive weeds (Walter 1999), agricultural plants such as citrus trees (Muma 1960, Olivier 1968, Ramsey et al. 1972a, Soliman and Mahfood 1978, Vacante and Nucifora 1986, Quilici et al. 1997, Grout and Ueckermann 1999, Ferla and Moraes 2002, Fadamiro et al. 2009), deciduous fruit trees (Nesbitt 1946, Garman 1948, Lord 1949, Ramsey et al. 1972b, Quilici et al. 1997, Ferla and Moraes 1998, Ferla and Moraes 2002; Shakhsi Zare et al. 2012), cotton (Kuznetzov and Sizova 1978), strawberries (Ferla et al. 2007), grape vineyards (Schruft 1971, Jubb et al. 1985, Molnar 1997), alfalfa fields (Badieritakis et al. 2014), and plants in urban settings (Lahiri et al. 2004); vertebrate nests (Garman 1948, Gupta and Chattopadhyay 1978, Gupta and Paul 1985, Estebanes-Gonzales 1997); caves (Cooreman 1954, Turk 1972, Zacharda 1978); animal debris (Corpuz Raros et al. 1988, Taha et al. 1988); tree holes (Atyeo 1958, Lin and Zhang 2002); house dust (Oliveria and Daemon 2003); and stored food products (Huges 1976, Tseng 1980, Fan 1992). Individual species, however, are thought to be restricted to a particular habitat. For example, *Armascirus taurus* is reported to be most prevalent on the leaves of citrus trees while *Coleoscirus simplex* and *Coleoscirus curtipalpus* are more common in the leaf litter (Muma 1965) and *Parabonzia bdelliformis* is usually collected from treeholes but not nearby litter (Smiley 1992).

While cunaxids are often often found on plants in agricultural settings, their effect on prey populations is unclear. Ewing and Webster (1912) observed *Cunaxoides parvus* feeding on oyster-shell scales on apple trees and Schruft (1971) reported *Cunaxoides oliveri* feeding on eriophyid mites on grapes. Sathiamma (1995) reported *Cunaxa setirostris* to be “a very active and efficient predator on all the motile stages of *Oligonychus iseilemae* [white spider mite]” and that the “predator larva preferred the larval prey; nymphal predator preferred the larvae and early nymphs of the prey and the adult preferred the prey nymphs and adults”. Nucifora and Vacante (1986) reported cunaxids to be auxiliary predators that are useful for crops, but not main predators used in “integrated control techniques”. Rigorous studies investigating the effect of cunaxids on prey populations, however, have not been conducted.

Cunaxids appear to be active year round. Den Heyer (1980a) collected all life stages of *Neocunaxoides* in the Transvaal Highveld during the summer (30 °C+) and winter (minimum 0 °C) months. Zaher et al. (1975b) collected cunaxids throughout the year and demonstrated a positive correlation between abundance and temperature; they also found a slight negative correlation between abundance and relative humidity.

Cunaxids have been reported to be found phoretically on bark beetles, though they were not identified to species (Penttinen et al. 2013).

Both sexual reproduction and thelytokous parthenogenesis have been reported in cunaxids (Walter and Proctor 1999, Castro and Moraes 2010). Within Cunaxinae, Coleoscirinae, and Cunaxoidinae, precopulatory guarding of the quiescent tritonymphal female has been reported (Walter and Kaplan 1991). *Dactyloscirus* males possess a well-developed, sclerotized aedeagus; *Armascirus* and *Rubroscirus* males also possess an aedeagus, though less developed and sclerotized than in *Dactyloscirus* (Den Heyer 1978a, 1979a, 1981a). Castro and Moraes (2010) suggest that *Cunaxatricha tarsospinosa* may be cyclically or facultatively parthenogenetic – one population they studied consisted entirely of females while another population approximately 450 km distant contained males – and that parthenogenesis may be induced by cellular endosymbionts.

Cunaxids spin silk, which is used for a variety of purposes. *Cunaxatricha tarsospinosa* produces a webbing around eggs laid on leaves, but not branches; Castro and Moraes (2010) report that destruction of webbing may reduce viability of the eggs. Nymphal *Armascirus taurus*, *Dactyloscirus inermis*, *Coleoscirus simplex*, and an undescribed *Pulaeus* construct silken molting chambers (Alberti and Ehrnsberger 1977; Walter and Kaplan 1991); the breadth of this behavior suggests it may be widespread among cunaxids. *Cunaxa setirostris* constructs an irregular net of two silk varieties which is used during prey capture (Alberti and Ehrnsberger 1977). It has also been proposed that some species may be venomous, though this has not been confirmed (Den Heyer 1980a, Smiley 1992, Walter and Proctor 1999).

**Biogeography.** Cunaxids have been found on every continent except Antarctica. South Africa and the Philippines have the most well-documented cunaxid diversity – 68 and 57 species respectively – thanks to the efforts of Den Heyer and Corpuz-Raros (Den Heyer 2011a). South America was little studied until Castro and Den Heyer described 8 genera and 10 species from Brazil between 2008 and 2009. Only two species are known from Australia, both reported by Womersley (1933), though Walter (1999) reported 5 undescribed species in 4 genera and Callan et al. (2011) reported another two species at the family level, suggesting many species await discovery there.

The cunaxid fauna of Europe and North America north of Mexico fall between these extremes. Most reports have been sporadic and span more than a century, beginning with Banks (1894) in the United States and Berlese (1887) in Europe. Robert L. Smiley, a well-known North American worker, never collected material. He instead worked on samples that were sent to him, often intercepted by the USDA at ports of entry, so rather than focusing on North American fauna he more generally worked on world species. This has led to a scattered understanding of the species and genera that occur in North America.

## Methods

The diagnoses and keys presented are based on published descriptions and examination of available type specimens. However, for many species the types were not available for examination. The accuracy of the keys is therefore dependent upon the accuracy of the published descriptions. This also influenced which characters were chosen for couplets. Often a character that is potentially useful and informative (such as the presence or absence of a cheliceral seta) was not reported in the original description. Thus, unlike previous keys, characters such as setal counts of leg segments were often preferred. This may prove to be problematic as extra setae are sometimes reported on leg segments; however, examination of multiple specimens in a population should help overcome this.

Den Heyer (2011b, 2013) transferred many species into different genera in the Bdelloidea database that is used by Species 2000 and ITIS Catalogue of Life (CoL). However, nomenclatural acts proposed within these databases are not considered valid under The International Code of Zoological Nomenclature as they do not conform to Article 8.4.2.2. This is intentional for a number of reasons, including avoiding circularity (e.g., a paper that cites CoL about a nomenclatural act, and CoL citing that paper) and time limitations in pursuing a publication that includes all nomenclatural acts proposed within the databases each year (Roskov and Bailly, 2 May 2014, pers. comm.).

### Terminology

An effort is made to utilize terminology that is broadly applicable and well-accepted across mite taxa, despite conventions used among bdelloid researchers. Some terms widely used by bdelloid researchers are either inaccurate or outdated, and others are misleading. Therefore, we follow the suggestions outlined by Fisher et al. (2011), which are elaborated upon below.

**Subcapitulum.** The part of the gnathosoma that bears the palps and chelicerae has been variously termed by researchers of Bdelloidea. One such term – hypostome – more properly refers to the area of the subcapitulum anterior to the oral opening (Evans 1992; Krantz and Walter 2009), and therefore its use in reference to the entire subcapitulum is incorrect. The other term – hypognathum – is synonymous with subcapitulum, and is therefore not inaccurate, but also not broadly used across mite taxa. Thus, we reject the use of hypognathum in favor of subcapitulum and reserve the use of hypostome to the region of the subcapitulum anterior to the oral opening.

**Body segmentation.** The terminology associated with the acariform idiosoma remains controversial. Classically, these regions have been most widely called the propodosoma and hysterosoma. However, Grandjean (1970) proposed an alternate view of acariform idiosomal organization based on a segmentation hypothesis of van der Hammen (1963). Grandjean postulated that the podosoma is dorsally overtaken by the gnathosoma and the opisthosoma and termed the outgrowth of the gnathosoma that obscures the propodosoma the ‘aspidosoma’. Under this hypothesis, referring to the anterio-dorsal half of the idiosoma as the propodosoma is inaccurate, while referring to posterio-dorsal idiosoma as the hysterosoma (opisthosoma + metapodosma) is more inclusive than necessary and should instead be denoted simply as the opisthosoma. This hypothesis has gained popularity and ‘aspidosoma’ is currently used across disparate acariform taxa (e.g., Caeculidae: Coineau 1974; Erythraeidae: Mąkol 2010; Penthalodidae: Jesionowska 2010; Tydeidae: Kazmierski 2008). Contrary to this, Weigmann (2001) pointed out there is neither evidence for the dorsal overgrowth of the gnathosoma obscuring the propodosoma, nor for the overgrowth of the opisthosoma obscuring the metapodosoma. Further, he provided good evidence for retaining ‘propodosoma’ and ‘hysterosoma’. Ultimately, this matter will not be resolved without detailed investigation into developmental biology. Barnett and Thomas (2012, 2013) investigated the embryology of an oribatid (*Archegozetes longisetosus* Aoki, 1965) and demonstrated the opisthosoma of that mite comprises only two segments. Unfortunately, their investigations are as yet unable to resolve the problem of the dorsal podosoma.

Fisher et al. (2011) proposed avoiding hypothesis-dependent terminology pending further evidence for a given hypothesis. Thus, they retained ‘hysterosoma’ to refer to the idiosoma posterior to the sejugal furrow and implemented ‘proterosoma’ for the anterior idiosoma. Both terms were considered hypothesis-independent, but suffered from being more inclusive than necessary. Regardless, ‘hysterosoma’ is already used by many authors to refer to the dorsum posterior to the sejugal furrow, therefore its implementation is uncontroversial. Conversely, ‘proterosoma’ is not widely used to refer to the anterior idiosoma. Thus, referring to those setae as ‘proterosomal setae’ is novel, and therefore less preferred. However, recent investigations provide some support for implementing ‘proterosoma’ – this is discussed below.

Phylogenetic analyses of large datasets that include molecular data has corroborated previous suspicions of the non-monophyly of “Acari” and provided substantial support for a clade that combines camel spiders with acariforms called Poecilophysidea (Dabert et al. 2010, Pepato et al. 2010). In addition to characteristics of the reproductive system that have been previously noted (Alberti 1980a, b, 2000, Alberti and Peretti 2002, Klann et al. 2009), Dunlop et al. (2012) suggested that the sejugal furrow of Acariformes is homologous to a similar body division in Solifugae, lending another potential synapomorphy for this clade. Because of this, the sejugal furrow was elevated as a key morphological trait among both camel spiders and acariforms, which now makes it possible to construct terminology founded in a well-supported hypothesis. This renders terms that are denoted relative to the sejugal furrow (like ‘proterosoma’ and ‘hysterosoma’) as hypothesis-dependent, which is only preferred over hypothesis-independent terminology when the hypothesis is well-supported.

Therefore, we continue with the suggestions of Fisher et al (2011) in using ‘proterosoma’ and ‘hysterosoma’ for two reasons: 1) they are hypothesis-independent with respect to Grandjean’s ‘aspidosoma’ and Weigmann’s ‘propodosoma’; and 2) since 2011, they have been found to be hypothesis-dependent, but on well-supported hypotheses. Obviously, as future research resolves the issue of the acariform idiosomal dorsum (i.e. Grandjean vs. Weigmann), we suggest that new terminology based on those hypotheses should be adopted.

**Idiosomal setae.** For hysterosomal setae, we follow the notation of Grandjean (1939, 1947) that has been widely adopted by acarologists (e.g., van der Hammen 1970; Lindquist 1976, 1977; Kethley 1990; Swift 1996). However, proterosomal setae remain problematic. Historically, proterosomal chaetotaxy followed Grandjean (1939, 1947), which identified internal/external verticals (*vi* and *ve*) and internal/external scapulars (*sci* and *sce*). This notation has always been cumbersome for groups like Bdelloidea which have *sci* always external to *sce*. Given that homology has not been determined for these setae across mite taxa, some authors suggested simply switching the designations of *sci* and *sce* to reflect their position (Den Heyer and Castro 2008a, b, c; Den Heyer 2011c). As a result, frustratingly, the literature now has both *sci* and *sce* referring to each set of setae.

Therefore, we reject the suggestion of Den Heyer and Castro (2008) and follow the suggestion of Fisher et al. (2011), which resorts to a modified version of Atyeo (1960) when referring to proterosomal setae: anterior/posterior trichobothria (*at*/*pt*), and lateral/median proterosomal setae (*lps*/*mps*). Obviously, once homology of these setae can be determined across mite taxa, we suggest revising the terminology accordingly.

**Figure 2.**
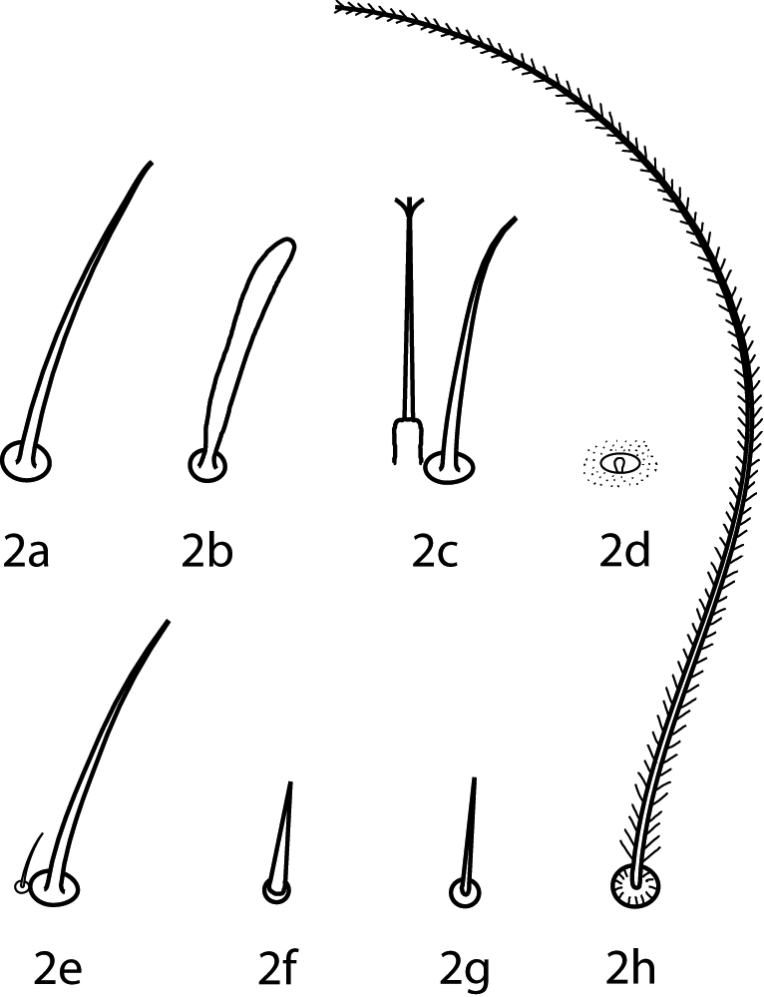
Setal types. Relative sizes will vary within a given setal type **2a** Attenuate solenidion (asl) **2b** Blunt rod-like solenidion (bsl) **2c** Elongate, tri-pronged famulus (fam), as seen in *Dactyloscirus* **2d** Famulus (fam), as seen in the majority of cunaxids **2e** Duplex setae - microseta (mst) and attenuate solenidion **2f** Spine-like seta (spls) **2g** Simple tactile seta (sts) **2h** Trichobothrium (T).

**Abbreviations.** The following abbreviations (Fig. 2) are used: attenuate solenidion (asl), blunt rod-like solenidion (bsl), famulus (fam)(=peg organ), microseta (mst), solenidion (s) (this is used only when a description does not specify what type of solenidion and may refer to any solenidion type), spine-like seta (spls), simple tactile seta (sts), trichobothrium (T). When setal types are not specified (e.g., coxae I–IV setal formula 5-5-4-3) it is assumed all setae are simple (sts).

Illustrations were produced using the methods outlined by Fisher and Dowling (2010).

## Systematics

### Cunaxidae

Taxon classificationAnimaliaTrombidiformesCunaxidae

Thor, 1902

#### Historical review.

Linnæus (1758) described *Acarus* and included all mites therein. Hermann separated three mite species with elongated gnathosomas (i.e., Bdellidae and Cunaxidae) from *Acarus* into *Scirus*. However, Hermann died in 1794 and his papers were not published until after his death by his brother-in-law F. L. Hammer in 1804 (as Hermann 1804). Latreille (1795) had by then separated the same mites into *Bdella*. Von Heyden (1826), recognizing that *Bdella* had priority over *Scirus*, synonomised *Scirus* with *Bdella* and erected *Cyta* and *Cunaxa*. However, many authors, including Dugés (1834a), Kramer (1881, Banks (1894), and Berlese (1904, 1910), continued to describe new species in *Scirus*. Dugés (1834a) erected Bdellidae (Bdelloidea) for *Bdella* and *Scirus*, having apparently not seen Von Heyden’s synonymization of the two genera. Trouessart (1892) moved *Cunaxa* from Bdellidae to Trombidiidae and erected the subfamily Scirinae. Oudemans (1902) used Cunaxinae in the same sense that Trouessart (1892) used Scirinae, that is for those mites in the family Bdellidae (*sensu* Dugés) that have pedipalps with a curved terminal segment and movable chela only (= Cunaxidae *sensu* Thor). Thor (1902) erected Cunaxidae as a family separate from Bdellidae. Oudemans (1906) disregarded Thor’s (1902) erection of Cunaxidae and kept Cunaxinae as a subfamily within Bdellidae. Van der Hammen (1972) erected the superfamily Cunaxoidea over Bdelloidea, disregarding the priority of *Bdella* Latreille (1795) over *Cunaxa* Von Heyden (1826). Den Heyer (1977b) erected Bonziinae for *Bonzia* and *Parabonzia*. Den Heyer (1978a) preserved the name Cunaxinae, but limited its concept to those cunaxids possessing 5-segmented pedipalps which extend past the subcapitulum by at least the distal two segments. Den Heyer (1978b) erected Coleoscirinae. Den Heyer (1980c) erected the monobasic Scirulinae and recognized the priority of Bdelloidea over Cunaxoidea. Bu and Li (1987a) erected Orangescirulinae. Smiley (1992) erected Denheyernaxoidinae, Neobonzinae, and Paracunaxoidinae as monotypic subfamilies and monographed and provided keys to known species. Den Heyer and Castro (2009) moved *Denheyernaxoides* and *Paracunaxoides* to Cunaxoidinae, thus disregarding Denheyernaxoidinae and Paracunaxoidinae as valid subfamilies. Lin and Zhang (2010) provided a detailed historical review of Cunaxidae in China and a checklist of species found in that country. Den Heyer (2011) moved *Neobonzia* to Coleoscirinae, effectively disregarding Neobonzinae, and synonymized *Coleobonzia* with *Neobonzia*.

#### Diagnosis.

**Figures 3–6.**
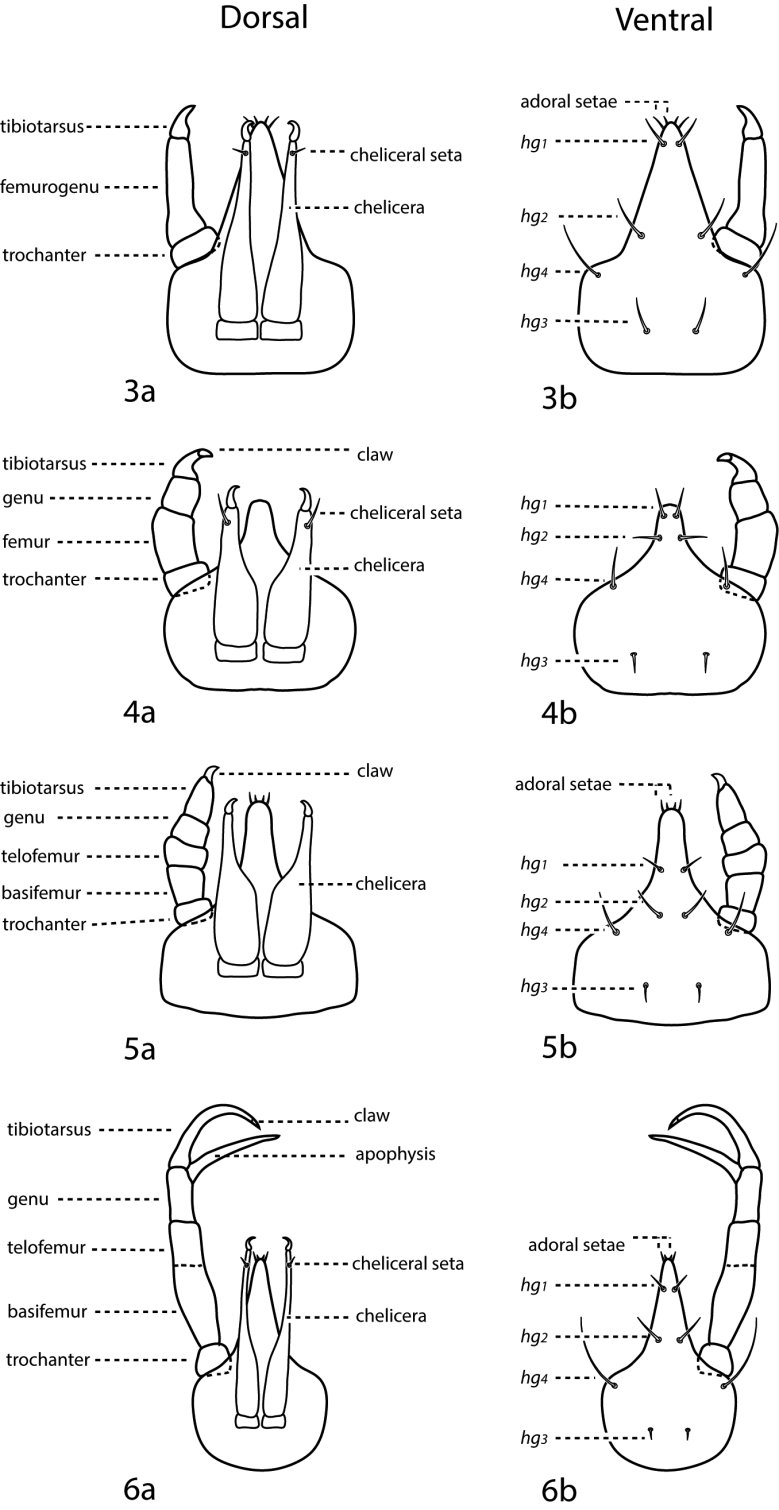
a. dorsal. b. ventral. **3** 3-segmented pedipalp (Cunaxoidinae) **4** 4-segmented pedipalp (Scirulinae) **5** 5-segmented pedipalp that does not extend beyond the subcapitulum by more than the distal half of the genua (Bonziinae, Coleoscirinae, and Orangescirulinae) **6** 5-segmented pedipalp that reaches beyond the subcapitulum by at least the distal half of the genua (Cunaxinae).

*Gnathosoma* (Figs 3–6). **Pedipalps** 3-, 4-, or 5-segmented and end in a strong claw (except in *Pseudobonzia*). They may be shorter than, equal to, or extend beyond the distal end of the subcapitulum. Femora of 5-segmented pedipalps divided into basi- and telofemora, though may be secondarily fused; a dark line often indicates the previous articulation (Fig. 5a, b illustrate a fully divided femur and Fig. 6a, b illustrate a secondarily fused femur. This is for illustration purposes only, i.e., cunaxids with long and short 5-segmented pedipalps may have either fully divided or secondarily fused femora). Telofemora and genua are uniquely fused in *Allocunaxa*, though the basifemoral/telofemoral articulation is present. Apophyses present or not on the telofemora, adjoining the genua and tibiotarsi, or on the tibiotarsi. Subcapitulum wedge-shaped and may be patterned with random dots or papillae, dots or papillae forming lines, a single row of cells on the posterior edge, or reticulations forming polygonal cells. **Subcapitulum** with up to 6 pairs of setae are present: *hg_1_*_–_*_4_* and 2 pairs of adoral setae. Seta *hg_1_* usually straight, but geniculate in Bonziinae and may be curved in *Neoscirula*; *hg_4_* often longest pair of subcapitular setae. **Chelicerae** with or without seta near the cheliceral digit.

**Figure 7.**
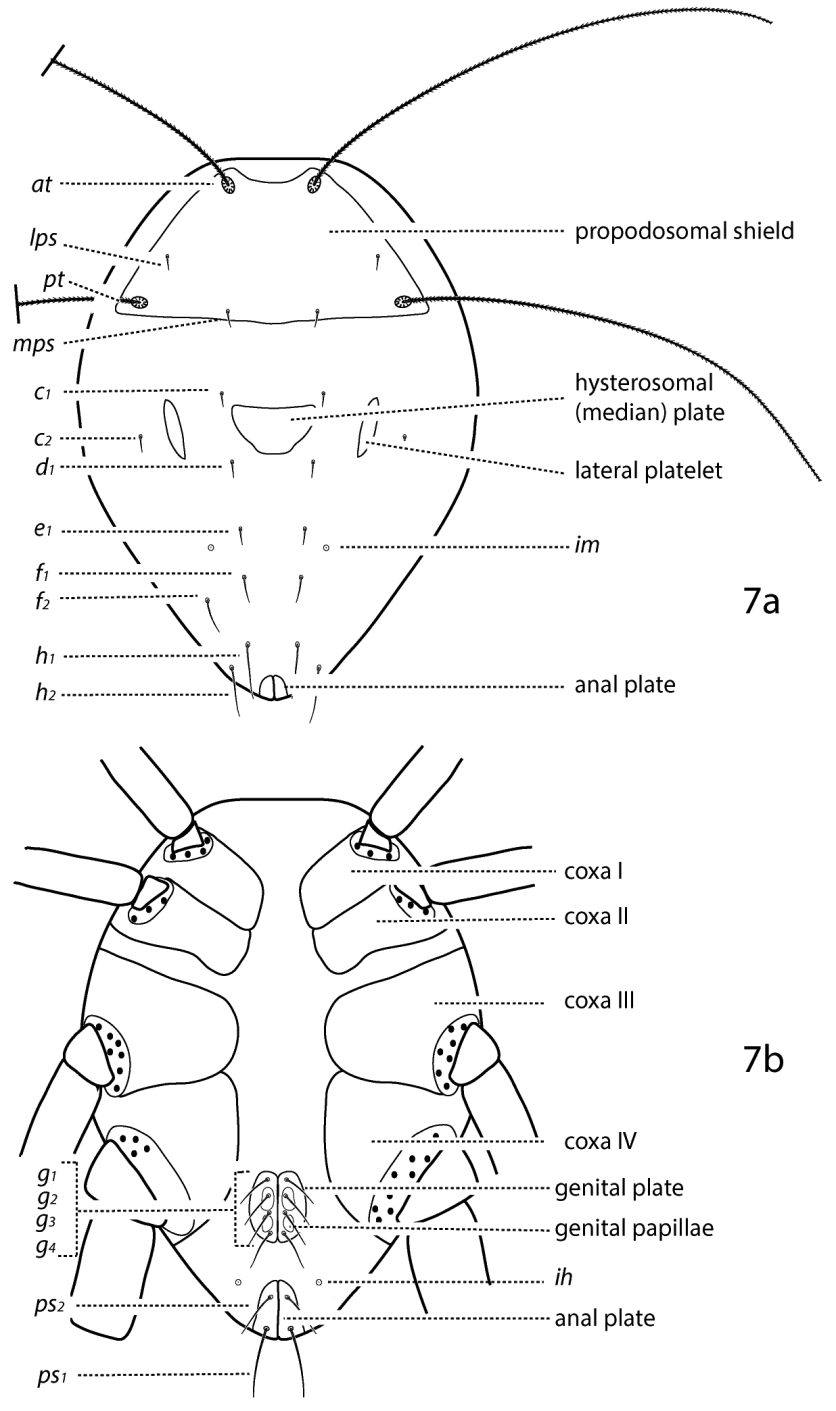
Generalized schematic of cunaxid idiosomal morphology. **7a** Dorsal. **7b** Ventral.

*Idiosoma, dorsal* (Fig. 7a). Idiosoma diamond-shaped. Dorsal proterosoma covered with a sclerotized shield that bears 2 pairs of setae (*lps* and *mps*) and 2 pairs of setose sensilla (*at* and *pt*); rarely one pair of setae or sensillae absent. Dorsal hysterosoma complemented with 0–2 large shields or plates and 0–4 pairs of platelets. These plates and platelets may capture one or more pairs of setae. Up to 8 pairs of dorsal hysterosomal setae present (*c_1_*–*h_1_*, *c_2_*, *f_2_*, and *h_2_*); *h_2_* may occur ventrally. Setae may occur on small platelets that are barely larger than the setal socket. Integument not covered in shields, plates, or platelets is striated. Cupule *im* present, usually laterad and slightly posterior to *e_1_*. Dorsal idiosomal shields and plates smooth or patterned with random dots or papillae, dots or papillae forming lines, reticulations forming polygonal cells, or cells which form rows.

*Idiosoma, ventral* (Fig. 7b) Ventral idiosoma may be complemented with 1 or a few small platelets in addition to the coxae. **Coxae** fused to body and form plates. Coxae I–II are often fused in adults and may coalesce medially to form a sternal shield. Coxae III–IV are often fused in adults and may extend caudally beyond the genital plates. Each coxa complemented with 0–4 setae; in addition, extensive coxae or sternal shields may capture setae normally on the integument and therefore have more. Coxae may be plain or patterned with random dots or papillae, dots or papillae forming lines, or reticulations forming polygonal cells. Genital plates (sometimes called anal valves) present in adults and bear 3 (rarely) or 4 (usually) setae, except in *Parabonzia* which have up to 9 pairs of setae. 2 pairs of genital papillae visible underneath the plates. Anal plates (sometimes called anal valves) bear 1–2 setae (*ps_1-2_*). Setae *ps_2_* may occur off the anal plates. **Legs** 6-segmented in larvae, 7-segmented in nymphs and adults. In adults these segments are coxa, trochanter, baifemur, telofemur, genu, tibia, and tarsus, however, the coxae are often treated separately from the other leg articles. Femora undivided in larvae. Trichobothrium present on leg tibia IV. Ambulacral claws present on either side of a 4-rayed empodium.

#### Key to Subfamilies of Cunaxidae

(modified from Smiley 1992)

**Table:** 

1	Pedipalpal telofemoral multi-branched seta present (except *Parabonzia mindanensis*) (Fig. 7a)	Bonziinae
–	Pedipalpal telofemoral multi-branched seta absent	2
2 (1)	Pedipalps 3-segmented (Figs 3a, b)	Cunaxoidinae
–	Pedipalps 4-segmented (Figs 4a, b)	Scirulinae
–	Pedipalps 5-segmented (basi-and telofemora may be partially fused) (Figs 5a, b; 6a, b)	3
3 (2)	Pedipalps extend beyond the subcapitulum by at most the distal half of the tibiae (Figs 5a, b)	4
–	Pedipalps extend beyond the subcapitulum by at least the distal half of the tibiae (Figs 6a, b)	Cunaxinae
4 (3)	Trichobothrium on tibiae IV present; setae hg1 not geniculate; cheliceral seta usually present	Coleoscirinae
–	Trichobothrium on tibiae IV absent; setae *hg_1_* geniculate; cheliceral seta absent	Orangescirulinae

### Bonzinae

Taxon classificationAnimaliaTrombidiformesCunaxidae

Oudemans, 1927

#### Historical review.

Oudemans (1927) erected *Bonzia* within Cunaxidae for *Bonzia halacaroides*. Smiley (1975) erected *Parabonzia* for *Bonzia bdelliformis*. Den Heyer (1975) erected *Cunabdella* for *Cunabdella marthae*. Den Heyer (1977b) erected Bonzinae for the two genera; he also moved *Cunabdella marthae* to *Parabonzia*, effectively synonymizing *Cunabdella* with *Parabonzia*.

#### Diagnosis.

*Gnathosoma*. **Pedipalps** 5-segmented and reach beyond the subcapitulum by at most the distal half of the tibiae. Apophyses absent. A multi-branched seta present dorsally on the telofemora. Tibiotarsi terminate in a stout claw or two strong setae. 2 pairs of adoral setae present or absent. **Subcapitulum** with 4 pairs of setae (*hg_1_*_–_*_4_*) present in *Bonzia*; up to 6 pairs of subcapitular setae (*hg_1-4_* + additional setae) present in *Parabonzia*.

*Idiosoma, dorsal*. Proterosoma bears a shield complemented with 2 pairs of setae (*at* and *pt*) and 2 pairs of setose sensillae (*lps* and *mps*). Dorsal hysterosoma may bear a shield; if a shield is present it may be complemented with a variable number of setae depending on the extent of the shield. Setae *c_1_*–*h_1_*, *c_2_*, *f_2_* and *h_2_* present and are smooth or spiculate. Cupule *im* present laterad and caudally of *e_1_*. Integument that does not bear shields or plates is striated.

*Idiosoma, ventral*. **Coxae** I–II fused or not and coxae III–IV fused or not. Genital plates bear 4–9 setae; 2 pairs of genital papillae visible underneath the plates. Up to 4 pairs of setae present on the anal plates. Up to 9 pairs of setae present on the integument between coxae II and the anal plates. **Legs.** Trichobothrium present on leg tibia IV. The ambulacral claws occur on either side of a 4-rayed empodium.

#### Key to adult female Bonziinae

(modified from Smiley 1992)

**Table:** 

1	Pedipalp tibiotarsal claw present; 2 pedipalp tibiotarsal spine-like tubercles present (Fig. 8b); genital plates with 4 pairs of setae; internal genital setae absent	*Bonzia* Oudemans, 1927
–	Pedipalp tibiotarsal claw absent; 2 pedipalp tibiotarsal spine-like tubercles absent (Fig. 8c); genital plates with 5–9 pairs of setae; internal genital setae present	*Parabonzia* Smiley, 1975

**Figure 8.**
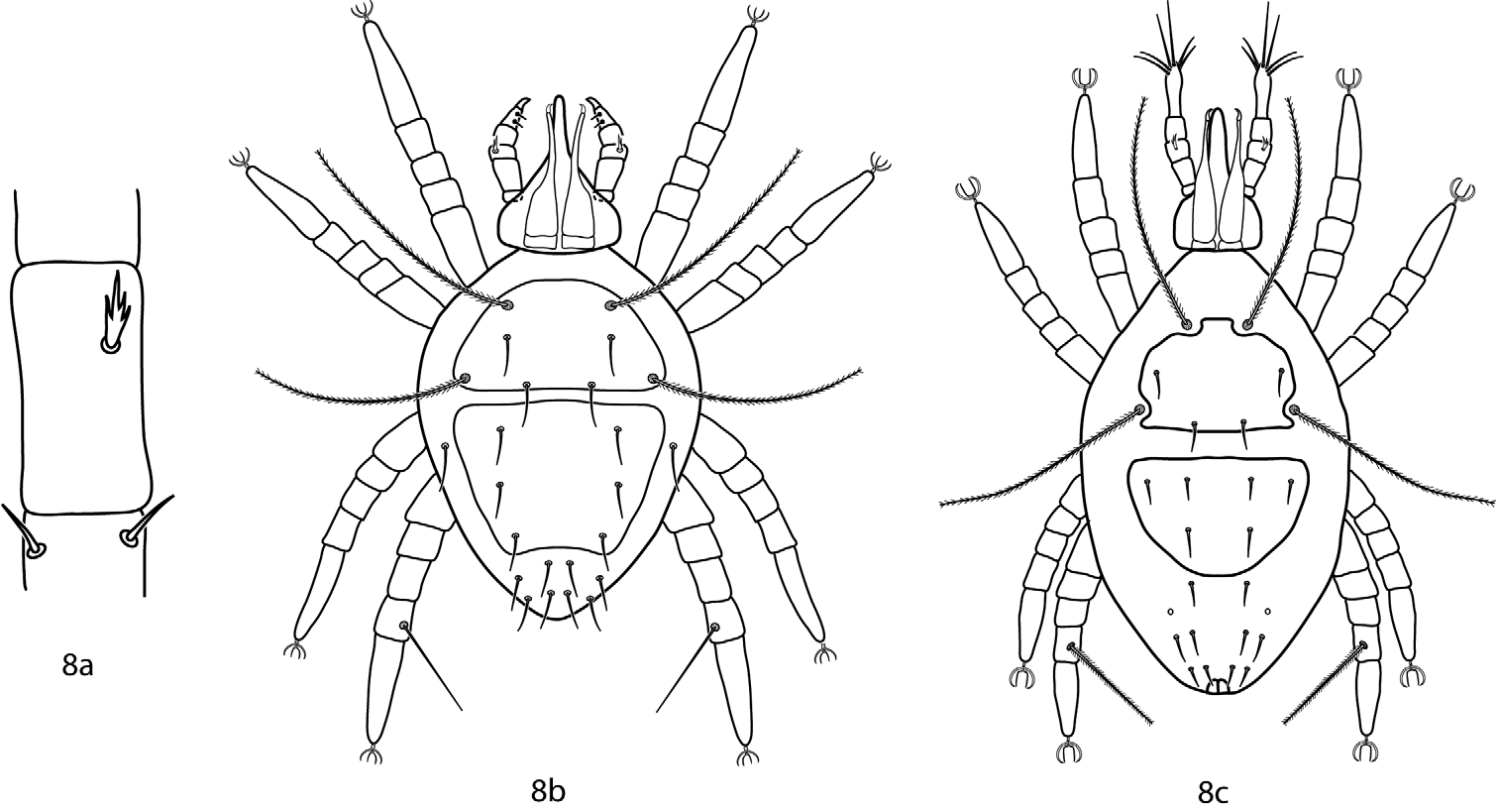
Bonziinae key illustrations. **8a** Telofemoral branched seta present in Bonziinae **8b** *Bonzia* **8c** *Parabonzia*.

### Bonzia

Taxon classificationAnimaliaTrombidiformesCunaxidae

Oudemans, 1927

#### Historical review.

Oudemans (1927) erected *Bonzia* for *Bonzia halacaroides*. Willmann (1939) described *Bonzia sphagnicola* from Germany. Willmann (1950) described *Bonzia rufofusca*. *Bonzia brownei* was described by Turk (1972). Den Heyer (1977) provided a detailed redescription of type material of this genus. Kuznetzov and Livshitz (1979) reported *Bonzia* from Russia. Michocka (1987) reported *Bonzia halacaroides* from Poland. Smiley (1992) described *Bonzia woodi* and *Bonzia yunkeri* and synonymized *Bonzia rufofusca* and *Bonzia brownei* with *Bonzia halacaroides*. Skvarla et al. reported *Bonzia yunkeri* from the Ozark Mountains in Arkansas.

#### Diagnosis.

*Gnathosoma*. **Pedipalps** 5-segmented and reach beyond the subcapitulum by at most the distal half of the tibiae. Apophyses absent. A dorsal multi-branched seta present on the telofemora. The tibiotarsi terminate in a stout claw. 2 pairs of adoral setae present or absent. **Subcapitulum** with 4 pairs of setae (*hg_1_*_–_*_4_*) present. Setae *hg_1_* are geniculate.

*Idiosoma, dorsal*. proterosoma bears a shield complemented with 2 pairs of setae (*at* and *pt*) and 2 pairs of setose sensillae (*lps* and *mps*). The dorsal hysterosoma bears a shield that may be complemented with a variable number of setae depending on the extent of the shield. Setae *c_1_*–*h_1_*, *c_2_*, *f_2_* and *h_2_* present, and are smooth or spiculate. Cupule *im* present laterad and caudally of *e_1_*. Integument that does not bear shields or plates is striated.

Coxae I–II fused and coxae III–IV fused. Genital plates bear 4 setae; 2 pairs of genital papillae visible underneath the plates. 4 pairs of setae present on the anal plates. Trichobothrium on leg tibia IV present. Ambulacral claws occur on either side of a 4-rayed empodium.

#### Key to adult female *Bonzia*

(modified from Smiley 1992)

**Table:** 

1	Tibiae IV trichobothrium setose (Fig. 9a)	2
–	Tibiae IV trichobothrium smooth (Fig. 9b)	3
2 (1)	Hysterosomal shield with 2 pairs of setae; Germany	*Bonzia sphagnicola* Willmann, 1939
–	Hysterosomal shield with 3 pairs of setae; N. America, S. America, Europe (possibly cosmopolitan)	*Bonzia halacaroides* Oudemans, 1927
3 (1)	Dorsal setae spiculate (Figs 10a, 11a); New Zealand	*Bonzia woodi* Smiley, 1992
–	Dorsal setae smooth (Figs 10b, 11b); USA: Virginia, Ozark Highlands	*Bonzia yunkeri* Smiley, 1992

**Figures 9–11.**
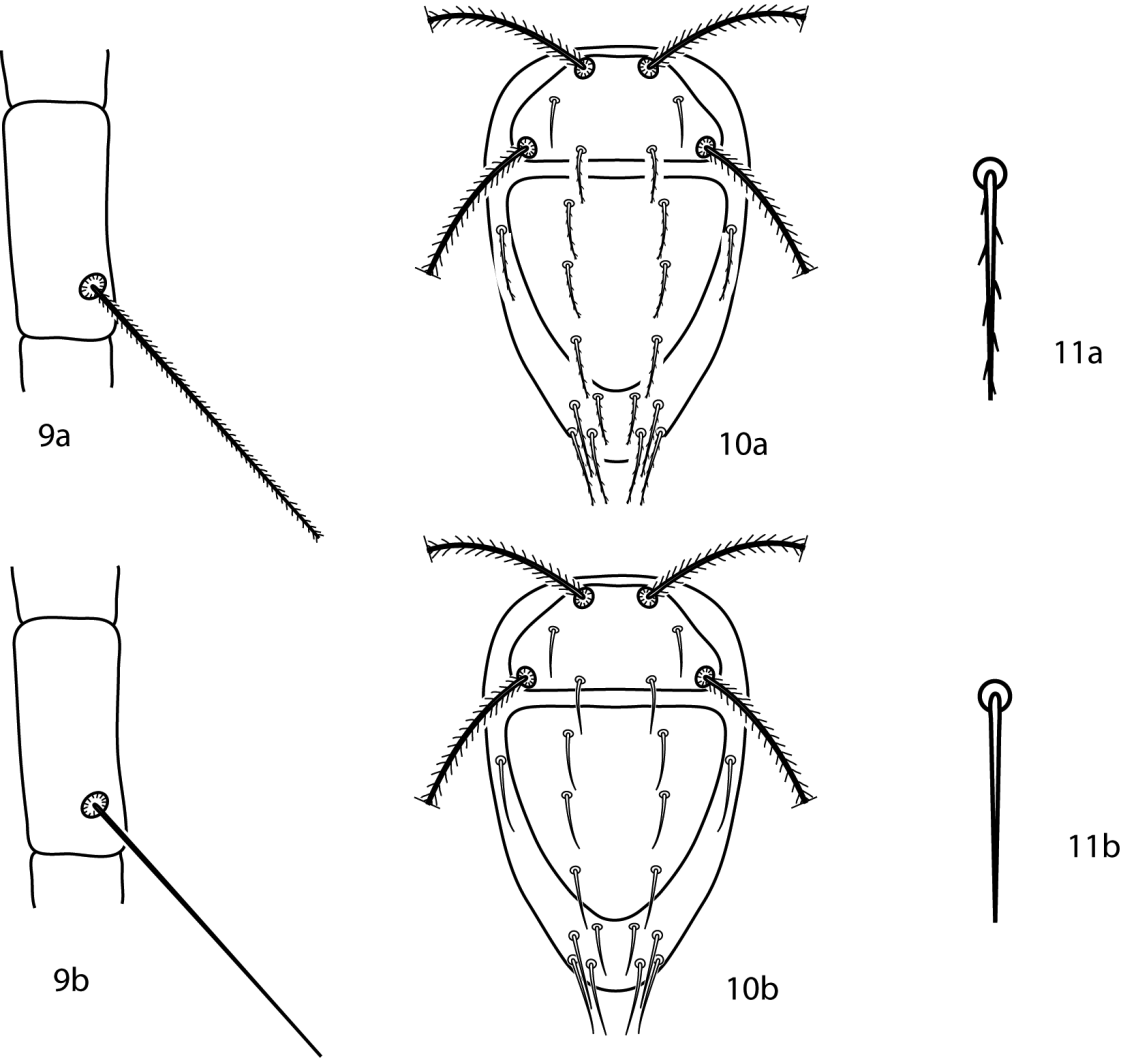
*Bonzia* key illustrations. **9a** Setose tibial trichobothrium **9b** Smooth tibial trichobothrium **10a** Spiculate dorsal setae **10b** Smooth dorsal setae **11a** Close up of a spiculate seta **11b** Close up of a smooth seta.

### Parabonzia

Taxon classificationAnimaliaTrombidiformesCunaxidae

Smiley, 1975

#### Historical review.

Atyeo (1958) described *Bonzia bdelliformis* from a tree hole in Tennessee, USA. Smiley (1975) erected *Parabonzia* for *Bonzia bdelliformis*. Den Heyer (1975) erected *Cunabdella* for *Cunabdella marthae*. Den Heyer (1977b) synonymized *Cunabdella* with *Parabonzia* and described *Parabonzia athiasae*. Kuznetzov and Livshitz (1979) reported *Parabonzia* from Russia. Smiley (1992) described *Parabonzia mumai* from Florida, USA. Corpuz-Raros (1996a) described *Parabonzia mindanensis* from the Philippines. Lin and Zhang (1998) described *Parabonzia trioxys*. Later they (Lin and Zhang 2002) described *Parabonzia zhangi*. Skvarla et al. (2013) reported *Parabonzia bdelliformis* from the Ozark Mountains in Arkansas.

#### Diagnosis.

*Gnathosoma*. **Pedipalps** 5-segmented and reach beyond the subcapitulum by at most the distal half of the tibiae. Apophyses absent. A multi-branched seta present dorsally on the telofemora. Tibiotarsi terminate in two strong setae. 2 pairs of adoral setae present or absent. **Subcapitulum** with up to 8 pairs of setae present.

*Idiosoma, dorsal*. Proterosoma bears a shield complemented with 2 pairs of setae (*at* and *pt*) and 2 pairs of setose sensillae (*lps* and *mps*). Dorsal hysterosoma may bear a shield; if a shield is present it may be complemented with a variable number of setae depending on the extent of the shield. Setae *c_1_*–*h_1_*, *c_2_*, *f_2_* and *h_2_* present and smooth. Cupule *im* is present laterad and caudally of *e_1_*. Integument that does not bear shields or plates is striated.

*Idiosoma, ventral*. **Coxae** I–II fused or not and coxae III–IV fused or not. Genital plates with up to 9 pairs of setae; 2 pairs of genital papillae visible underneath the plates. Up to 4 pairs of setae present on the anal plates. Up to 9 pairs of setae on the integument between coxae II and the anal plates. **Legs.** Trichobothrium on leg tibia IV present. The ambulacral claws occur on either side of a 4-rayed empodium.

#### Key to adult female *Parabonzia*

**Table:** 

1	8–9 genital setae present	2
–	6–7 genital setae present	3
2 (1)	Pedipalpal telofemoral seta unbranched (Fig. 12a); Philippines, Mindanao Is	*Parabonzia mindanensis* Corpuz-Raros, 1996
–	Pedipalpal telofemoral seta branched, with 4–5 tines (Fig. 12b); China: Hubei Province	*Parabonzia zhangi* Lin & Zhang, 2002
3 (1)	Hysterosomal shield with 3 pairs of setae	4
–	Hysterosomal shield with 4 pairs of setae	6
4 (3)	Pedipalpal tibiotarsal sigmoid setae lightly barbed (Fig. 13); South Africa: West Transvaal	*Parabonzia marthae* (Den Heyer, 1975)
–	Pedipalpal tibiotarsal sigmoid setae smooth	5
5 (4)	Large spur-like process present on femora III (Fig. 14); USA: Florida	*Parabonzia mumai* Smiley, 1992
–	Large spur-like process absent on femora III; Ivory Coast	*Parabonzia athiasae* Den Heyer, 1977
6 (3)	Coxae I–IV setal formula 7-5-6-7 sts; basifemora I–IV setal formula 4-7-3-2 sts; China: Fujian	*Parabonzia trioxys* Lin & Zhang, 1998
–	Coxae I–IV setal formula 6-6 (sometimes 7)-7-7 sts; basifemora I–IV setal formula 5-8-3-2 sts; USA, Russia	*Parabonzia bdelliformis* (Atyeo, 1958)

**Figures 12–14.**
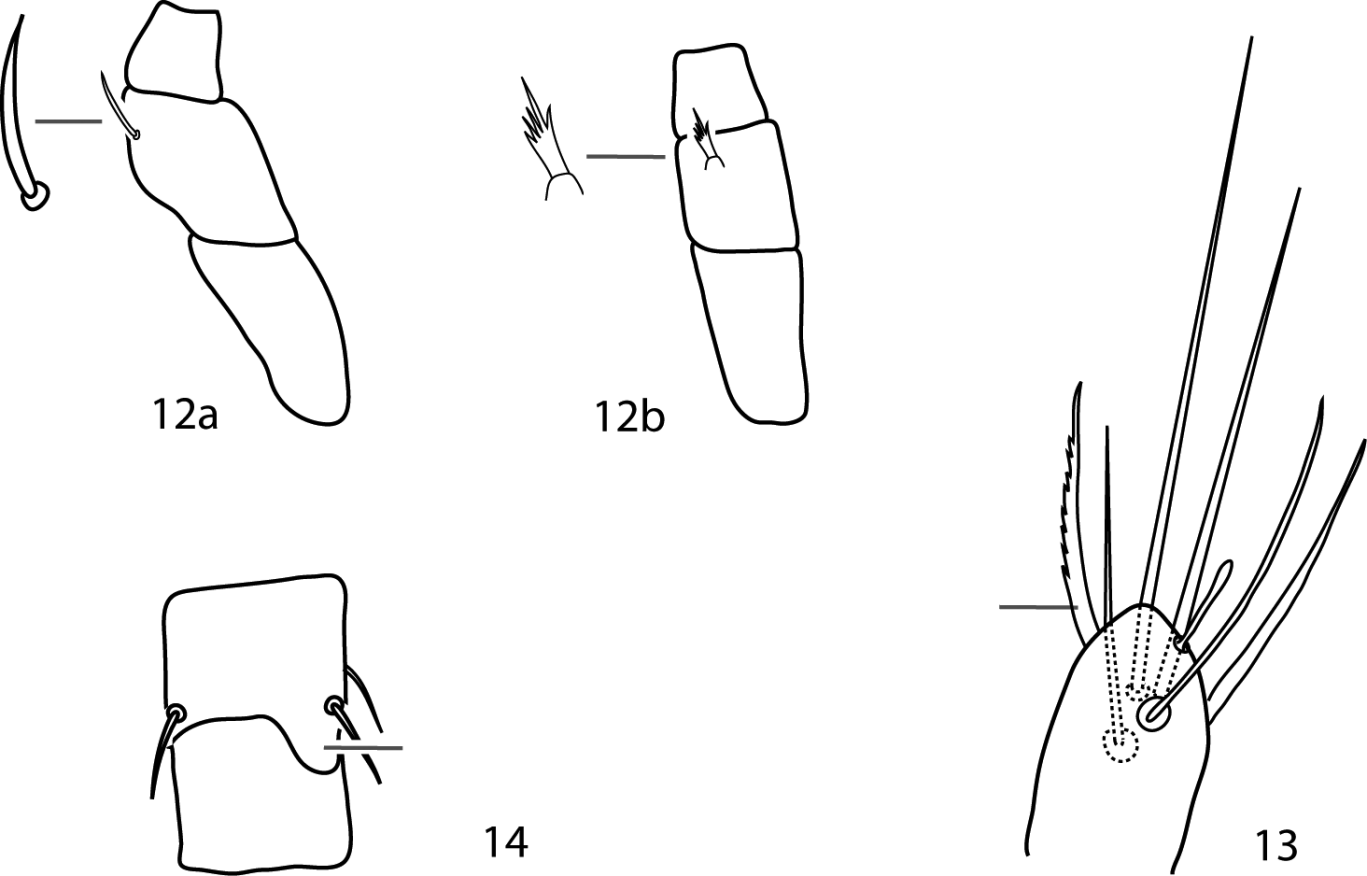
*Parabonzia* key illustrations. **12a** Unbranched pedipalp telofemoral seta **12b** Multi-branched pedipalp telofemoral seta **13** Lightly barbed pedipalp tibiotarsal sigmoid seta **14** Spur-like process on femora III.

### Cunaxoidinae

Taxon classificationAnimaliaTrombidiformesCunaxidae

Den Heyer, 1978

#### Historical review.

Koch (1838) established *Eupalus* and described the first mite belonging to Cunaxoidinae, *Eupalus croceus*. Baker and Hoffmann (1948) proposed *Cunaxoides* to replace *Eupalus* Koch as the name was preoccupied (a fact that acarologists had missed for 100 years) by *Eupalus* Gistl; they also redescribed and reillustrated a number of known species. Radford (1950) proposed *Haleupalus* to replace *Eupalus*, though this name is invalid because it is predated by *Cunaxoides*. Smiley (1975) erected *Neocunaxoides* and reviewed *Cunaxoides*. Both genera were assigned to the newly established Cunaxoidinae by Den Heyer (1978c). *Pulaeus* was established by Den Heyer (1979b); the name is an anagram and nod to *Eupalus*. Den Heyer (1979c) erected *Scutopalus* for those cunaxoidines with well-demarcated dorsal and ventral plates. Smiley (1992) synonymized *Scutopalus* with *Neocunaxoides* and *Haleupalus* with *Cunaxoides*; he also erected *Denheyernaxoides* and *Paracunaxoides* as monotypic genera in two new subfamilies, Denheyernaxoidinae and Paracunaxoidinae respectively. Castro and Den Heyer (2009) split a new genus, *Lupaeus*, from *Pulaeus* based on the number of setae on basifemora IV (1 and 2, respectively) and the number of pointed processes on the pedipalpal tibiotarsi (2 and 1, respectively). Den Heyer and Castro (2009) split *Bunaxella*, *Dunaxeus*, *Funaxopsis*, and *Qunaxella* from *Cunaxoides*; they also moved *Denheyernaxoides* and *Paracunaxoides* to Cunaxoidinae, thus disregarding Denheyernaxoidinae and Paracunaxoidinae as valid subfamilies.

#### Diagnosis.

*Gnathosoma*. **Pedipalps** 3-segmented: a trochanter which lacks setae, fused femurogenu (femur + genu) which is complemented with 5 or 6 setae, and tibiotarsus (tibia + tarsus) which is complemented with 5 or 6 setae. Tibiotarsi may be complemented with a bladder- or bulb-like apophysis. Pedipalps do not reach beyond the subcapitulum by more than the distal half of the tibiotarsi. **Chelicera** with or without seta near the cheliceral digit. **Subcapitulum** with 4 pairs of setae (*hg_1_*_–_*_4_*) are present; setae *hg_4_* is often the longest. 2 pairs of adoral setae are present or absent.

*Idiosoma, dorsal*. Female with proterosomal shield (absent in *Cunaxoides ulcerosus*) which is complemented with two pairs of setae (*lps* and *mps*) and two pairs of setose sensillae (*at* and *pt*) and may bear a hysterosomal plate complemented with a varying number of setae; when present the dorsal hysterosomal plate may be fused with the proterosomal shield. Dorsal plates well demarcated or not. Dorsal setae *c_1_*–*h_1_* are present; *c_2_*, *f_2_* and *h_2_* may also be present. If *f_2_* is present, *f_1_* and *f_2_* may be located together on a small platelet. Setae not on larger plates may be born on small platelets barely larger than the setal socket. Cupule *im* present laterad and posterior of *e_1_*. Integument that is not covered in shields or plates is striated

*Idiosoma, ventral*. **Coxae** of female vary in size, from being restricted to the trochantral bases to being extensive and nearly forming a holoventral shield. Coxae may or may not be well demarcated. Coxae I–II fused (usually) or not, coxae III–IV fused (usually) or not. Coxae I–II may coalesce medially to form a sternal shield. The genital plates each bear 4 setae (*g_1_*_–_*_4_*); 2 pairs of genital papillae visible underneath the plates. The anal plates bear one pair of setae (*ps_1_*); one pair of setae is present ventrally on the integument near the anal plates (either *ps_2_* or *pa*). Cupule *ih* is present ventrally laterad the integumental setae associated with the anal plates. The integument that is not covered in shields or plates is striated. **Legs.** Tarsi never constricted apically so as to end in lobes. Trichobothrium on leg tibia IV present. Ambulacral claws are rippled and occur on either side of a 4-rayed empodium.

#### Key to adult female Cunaxoidinae

(modified from Den Heyer and Castro 2009)

**Table:** 

1	Pedipalpal tibiotarsi with 3 sts, 1 spls; New Zealand	*Paracunaxoides* Smiley, 1992
–	Pedipalpal tibiotarsi with 5 or 6 sts, 0 spls	2
2 (1)	Pedipalpal femurogenu with 5 setae; long setae ending in terminal bulb-like knob (very small) on tarsi III and IV present; telofemoral setal formula not 5-5-4-3; usually 6 setae on pedipalp tibiotarsus Cunaxoidini	3
–	Pedipalpal femurogenu with 6 setae; long setae ending in terminal bulb-like knob (very small) on tarsi III and IV absent; telofemoral setal formula 5-5-4-3; usually 5 setae on pedipalp tibiotarsus Pulaeini	9
3 (2)	Femora I and II divided; setae *f_2_* absent; trichobothrium on tibiae IV present or absent	4
–	Femora I and II not divided; setae *f_2_* present; trichobothrium on tibiae IV absent	*Denheyernaxoides* Smiley, 1992
4 (3)	Dorsum with ill-defined weakly sclerotized dorsal plates (Fig. 15a); subterminal pointed process on pedipalp tibiotarsal claw present (Fig. 16a); small teeth (=serrated edge) on pedipalp tibiotarsal claw present (Fig. 16a); cheliceral setae absent	5
–	Dorsum with well-defined and sclerotized dorsal plates (Fig. 15b); subterminal pointed process on pedipalp tibiotarsal claw absent (Fig. 16b); small teeth on pedipalp tibiotarsal claw absent (Fig. 16b); cheliceral setae present	*Scutopalus* Den Heyer, 1979
5 (4)	Trichobothrium on tibiae IV present; famulus present, on distal portion of tarsus I	*Cunaxoides*
–	Trichobothrium on tibiae IV absent; famulus present or absent	6
6 (5)	Tibiae III with 1 bsl, 3–5 sts; tibiae IV with 2 or 4 sts	7
–	Tibiae III with 1 lts, 4 sts; tibiae IV with 1 lsts, 4 sts	*Dunaxeus* Den Heyer & Castro, 2009
7 (6)	Tibiae III with 1 bsl, 3–5 sts; tibiae IV with 1 lts, 2 sts	*Funaxopsis* Den Heyer & Castro, 2009
–	Tibiae III with 1 bsl, 5 sts; tibiae IV setal formula not as above	8
8 (7)	Tibiae IV with 1 lsts, 4 sts; famulus present	*Qunaxella* Den Heyer & Castro, 2009
–	Tibiae IV with 4 sts; famulus absent	*Bunaxella* Den Heyer & Castro, 2009
9 (2)	Setae *f_2_* present; basifemora I–IV setal formula 4-6-3-1 or 4-6-3-2	10
–	Setae *f_2_* absent; basifemora I–IV setal formula 3-5-2-0 (rarely with 3-5-2-1)	*Neocunaxoides* Smiley, 1975
10 (9)	Basifemora I–IV setal formula 4-6-3-2; pedipalp tibiotarsus with one pointed process (ventral) (Fig. 17a); famulus on proximal half of tarsus I; tibiae I–II with non-striated blunt solenidia	*Pulaeus* Den Heyer, 1979
–	Basifemora I–IV setal formula 4-6-3-1; pedipalp tibiotarsus with two pointed processes (1 ventral, 1 median) (Fig. 17b); famulus on distal half (subapical) of tarsus I; tibiae I–II with transversely striated blunt solenidia	*Lupaeus* Castro & Den Heyer, 2009

**Figures 15–17.**
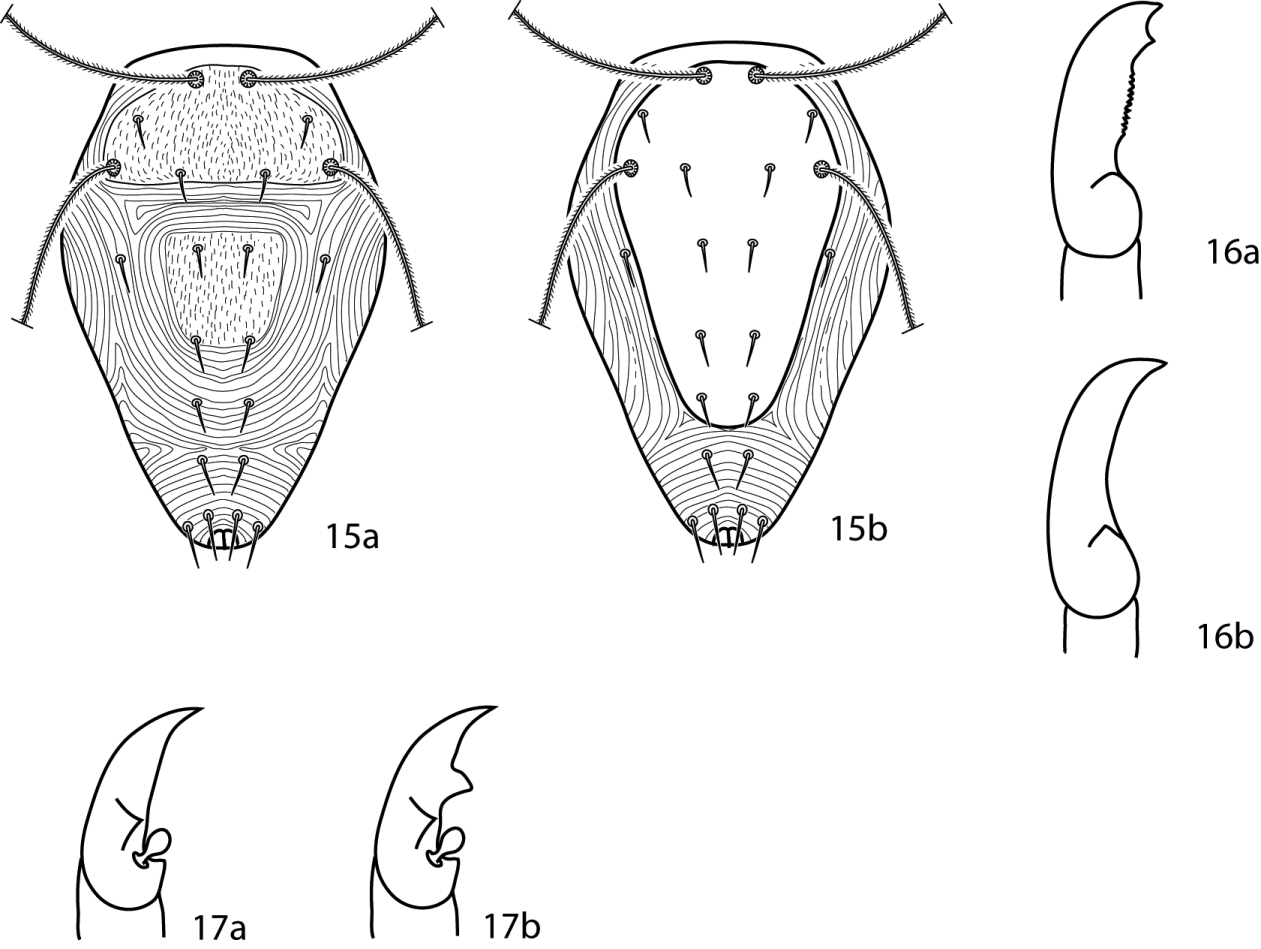
Cunaxoidinae key illustrations. Setae are removed from figures **16–17** for clarity **15a** Idiosoma with poorly demarcated dorsal plates **15b** Idiosoma with well demarcated dorsal plates **16a** Pedipalp tibiotarsus with subapical process and small teeth present **16b** Pedipalp tibiotarsus with subapical process and small teeth absent **17a** Pedipalp tibiotarsus with a single pointed process **17b** Pedipalp tibiotarsus with two pointed processes.

### Bunaxella

Taxon classificationAnimaliaTrombidiformesCunaxidae

Den Heyer & Castro, 2009

#### Historical review.

Den Heyer (1981b) described *Cunaxoides oribensis*, *Cunaxoides quini*, and *Cunaxoides zebedielensis*. Den Heyer and Castro (2009) erected *Bunaxella* and transferred *Cunaxoides oribensis*, *Cunaxoides quini*, and *Cunaxoides zebedielensis* to the new genus.

#### Diagnosis.

*Gnathosoma*. **Pedipalps** 3-segmented. Femurogenua are at least twice as long as wide and complemented with 5 setae. Tibiotarsi at least twice as long as wide and usually complemented with 6 setae. A small apophysis present basally and a pointed process occurs near the terminal tip; a ridge present between the apophysis and pointed process. **Subcapitulum** with 6 pairs of setae (*hg_1_*_–_*_4_* and 2 pairs of adoral setae) present; setae *hg_4_* is often the longest. **Chelicera** without seta.

*Idiosoma, dorsal*. Proterosoma bears an ill-defined and weakly sclerotized shield which is complemented with 2 pairs of setae (*lps* and *mps*) and 2 pairs of setose sensillae (*at* and *pt*). The dorsal hysterosoma may or may not bear a plate; if a plate is present it is ill-defined and weakly sclerotized, may be complemented with a variable number of setae, and may or may not be fused with the proterosomal shield. Setae *c_1_*–*h_1_*, *c_2_*, and *h_2_* are present. Seta *c_2_* plumose or fan-shaped. Cupule *im* is present laterad and posterior of *e_1_*. Integument that is not covered in shields or plates is striated.

*Idiosoma, ventral*. **Coxae** are weakly sclerotized and ill-defined; they can be recognized by possessing somewhat denser striations than the surrounding integument. Coxae I–II may be fused and may coalesce medially to form a sternal shield. Coxae III–IV fused or not. Each coxa complemented with 2-4 setae. Genital plates each bear 4 setae (*g_1_*_–_*_4_*); 2 pairs of genital papillae visible underneath the plates. Anal plates bear one pair of setae; one pair of setae is present ventrally on the integument near the anal plates. Up to 7 pairs of setae present on the integument between the coxal and genital plates. Cupule *ih* present ventrally laterad the integumental setae associated with the anal plates. Integument that is not covered in shields or plates is striated. **Legs.** Tarsi are never constricted apically so as to end in lobes. Depression for the famulus on tarsus I is absent. Tibia III complemented with 1 bsl, 5 sts. Tibia IV is complemented with 4 sts and lacks a trichobothrium. Ambulacral claws occur on either side of a 4-rayed empodium.

#### Key to adult female *Bunaxella*

(modified from Den Heyer and Castro 2009)

**Table:** 

1	Basifemora I–IV with 3-3-3-0 sts; telofemora IV with 1 sts; dorsal setae fan-shaped, except for smooth *f_2_*	*Bunaxella quini* (Den Heyer, 1981)
–	Basifemora I–IV with 4-4-3-1 sts; telofemora IV with 2 sts; dorsal setae plumose, except for *h_2_* which may be plumose or smooth	2
2 (1)	Setae *h_2_* plumose	*Bunaxella oribensis* (Den Heyer, 1981)
–	Setae *h_2_* smooth	*Bunaxella zebedielensis* (Den Heyer, 1981)

### Cunaxoides

Taxon classificationAnimaliaTrombidiformesCunaxidae

Baker & Hoffmann, 1948

#### Historical review.

Koch (1838) described the first two *Cunaxoides* as *Eupalus croceus* and *Eupalus minutissimus*. Koch (1841) described *Eupalus vitellinus*. Trägårdh (1910) described *Eupalus minima*. Ewing (1917) described *Eupalus parvus* and its feeding on oyster-shell scale in the USA. Thor and Willmann (1941) redescribed and figured *Eupalus croceus*, *Eupalus minutissimus*,﻿ and *Eupalus vitellinus*. Nesbitt (1946) described *Eupalus biscutum*. Garman (1948) reported *Eupalus biscutum* from apple trees in Connecticut. Baker and Hoffmann (1948) recognized that the name *Eupalus* was preoccupied and erected *Cunaxoides* to replace it; they transferred all known *Eupalus* to the new genus and figured each species. *Haleupalus oliveri* was described by Schruft (1971). Smiley (1975) synonymized *Cunaxoides vitellinus* with *Cunaxoides croceus* and provided a translation of Thor and Willmann’s (1941) description of *Cunaxoides croceus*. Den Heyer (1978c) placed *Cunaxoides* as the type genus in the newly erected Cunaxoidinae; he also redescribed the genus and redescribed and designated a neotype for *Cunaxoides croceus*. Kuznetzov and Livshitz (1979) described *Cunaxoides ulcerosus*, *Cunaxoides longistriatus*, *Cunaxoides fidus* and *Cunaxoides desertus* and reported and figured *Cunaxoides biscutum*, and *Cunaxoides parvus* from Russia. Gupta and Ghosh (1980) described *Cunaxoides nicobarensis*. *Cunaxoides kielczewskii* was described by Michocka (1982). Smiley (1992) synonymized *Haleupalus oliveri* with *Cunaxoides biscutum*, effectively synonymizing *Haleupalus* with *Cunaxoides*. Hu (1997) reported *Cunaxoides croceus* and *Cunaxoides ulcerosus* from China. Sionti and Papadoulis (2003) described *Cunaxoides paracroceus* from Greece. Bashir and Afzal (2004a) described *Cunaxoides trisetosis*. Bashir et al. (2007) described *Cunaxoides sargodhaensis* from Pakistan. Bashir and Afzal (2009) described *Cunaxoides daskaensis*, *Cunaxoides negans*, and *Cunaxoides sialkotensis* Den Heyer et al. (2013) described *Cunaxoides decastroae* and *Cunaxoides lootsi*.

#### Diagnosis.

*Gnathosoma*. **Pedipalps** 3-segmented. Femurogenua at least twice as long as wide and complemented with 5 setae. Tibiotarsi at least twice as long as wide and usually complemented with 6 setae. A small apophysis present basally and a pointed process present near the terminal tip; a ridge present between the apophysis and pointed process. **Subcapitulum** with 6 pairs of setae (*hg_1_*_–_*_4_* and 2 pairs of adoral setae) are present; setae *hg_4_* longest. **Chelicera** without seta.

*Idiosoma, dorsal*. Proterosoma bears an ill-defined and weakly sclerotized shield which is complemented with 2 pairs of setae (*lps* and *mps*) and 2 pairs of setose sensillae (*at* and *pt*). The dorsal hysterosoma may or may not bear a plate; if a plate is present it is ill-defined and weakly sclerotized, may be complemented with a variable number of setae, and may or may not be fused with the proterosomal shield. Setae *c_1_*–*h_1_*, *c_2_*, and *h_2_* are present. Cupule *im* present laterad and posterior of *e_1_*. Integument that is not covered in shields or plates is striated.

*Idiosoma, ventral*. **Coxae** weakly sclerotized and ill-defined; they can be recognized by possessing somewhat denser striations than the surrounding integument. Coxae I–II may be fused and may coalesce medially to form a sternal shield. Coxae III–IV may be fused. Each coxa is complemented with 2-4 setae. Genital plates each bear 4 setae (*g_1_*_–_*_4_*); 2 pairs of genital papillae visible underneath the plates. Anal plates bear one pair of setae; one pair of setae present ventrally on the integument near the anal plates. Up to 7 pairs of setae present on the integument between the coxal and genital plates. Cupule *ih* present ventrally laterad the integumental setae associated with the anal plates. Integument that is not covered in shields or plates is striated. **Legs.** Tarsi never constricted apically so as to end in lobes. Trichobothrium present on leg tibia IV. Ambulacral claws are rippled and occur on either side of a 4-rayed empodium.

#### Key to adult female *Cunaxoides*

The following species have not been included because the original descriptions and subsequent papers describing them (Thor and Willmann 1941; Baker and Hoffmann 1948) are not in English; known illustrations do not contain enough detail; and the types were not examined: *Cunaxoides minima* (Trägårdh, 1910), *Cunaxoides minutissimus* (Koch, 1938), *Cunaxoides vitellinus* (Koch, 1941).

**Table:** 

1	Dorsal hysterosomal median plate present (may be fused with proterosomal shield or only suggested by cuticular pattern) (Figs 18a–c, 19a–d, 20)	2
–	Dorsal hysterosomal median plate absent (Figs 21a, b, 22)	9
2 (1)	Hysterosomal median plate obvious, sclerotized (Figs 18a–d, 19a–c)	3
–	Hysterosomal median plate not be obvious or sclerotized, may only be suggested by cuticular pattern (Fig. 20)	8
3 (2)	Hysterosomal median plate not complemented with setae; USA	*Cunaxoides parvus* (Ewing, 1917)
–	Hysterosomal median plate complemented with setae	4
4 (3)	Hysterosomal median plate and proterosomal shield separate (Figs 18a–c)	5
–	Hysterosomal median plate and proterosomal shield fused (Figs 19a–d)	6
5 (4)	Hysterosomal median plate complemented with *c_1_*, *d_1_* (Fig. 18a); Canada, USA	*Cunaxoides biscutum* (Nesbitt, 1946)
–	Hysterosomal median plate complemented with *c_1_*, *d_1_*, *c_2_* (Fig. 18b); Russia	*Cunaxoides fidus* Kuznetzov & Livshitz, 1979
–	Hysterosomal median plate complemented with *c_1_*–*e_1_*, *c_2_* (Fig. 18c); Russia	*Cunaxoides longistriatus* Kuznetzov & Livshitz (1979
6 (4)	Hysterosomal shield complemented with setae *c_1_*, *d_1_*, *c_2_*; (Figs 19a, b)	7
–	Hysterosomal shield complemented with setae *c_1_- e_1_*, *c_2_*; (Figs 19c)	*Cunaxoides decastroae* Den Heyer, 2013
7 (6)	Genua IV with 1 asl, 5 sts; striae between *sci* and *c_1_* U-shaped (Fig. 19a); Greece	*Cunaxoides paracroceus* Sionti & Papadoulis, 2013
–	Genua IV with 2 asl, 5 sts; striae between *sci* and *c_1_* parallel (Fig. 19b); Europe	*Cunaxoides croceus* (Koch, 1838)
8 (2)	Dorsal striae form one “shield-like” area, similar to fused proterosomal and hysterosomal shield (Fig. 23a); Poland	*Cunaxoides kielczewskii* Gupta & Ghosh, 1980
–	Dorsal striae form two “shield-like” areas, similar to separate proterosomal and hysterosomal shields (Fig. 23b); Iran	*Cunaxoides lootsi* Den Heyer, 2013
9 (1)	Proterosomal shield present (Figs 21a, b)	10
–	Proterosomal shield absent (Fig. 22); Russia	*Cunaxoides ulcerosus* Kuznetzov & Livshitz (1979)
10 (9)	Dorsal shield reticulated (Fig. 21a); Russia	*Cunaxoides desertus* Kuznetzov & Livshitz (1979)
–	Dorsal shield striated (Fig. 21b)	11
11 (10)	Telofemora I–III setal formula 4-3-3; India	*Cunaxoides nicobarensis* Gupta & Ghosh, 1980
–	Telofemora I–III setal formula 5-5-4 or 5-5-6	12
12 (11)	Telofemur III with 3 sts; Pakistan	*Cunaxoides sialkotensis* Bashir & Afzal, 2009
–	Telofemur III with 4 sts	13
–	Telofemur III with 6 sts; Pakistan	*Cunaxoides negans* Bashir & Afzal, 2009
13 (12)	Basifemur I with 1 sts	14
–	Basifemur I with 2 sts; Pakistan	*Cunaxoides daskaensis* Bashir & Afzal, 2009
14 (13)	Basifemora II–IV setal formula 1-1-0; Pakistan	*Cunaxoides trisetosis* Bashir & Afzal, 2004
–	Basifemora II–IV setal formula 4-2-0; Pakistan	*Cunaxoides sargodhaensis* Bashir, Afzal & Raza, 2007

**Figures 18–23.**
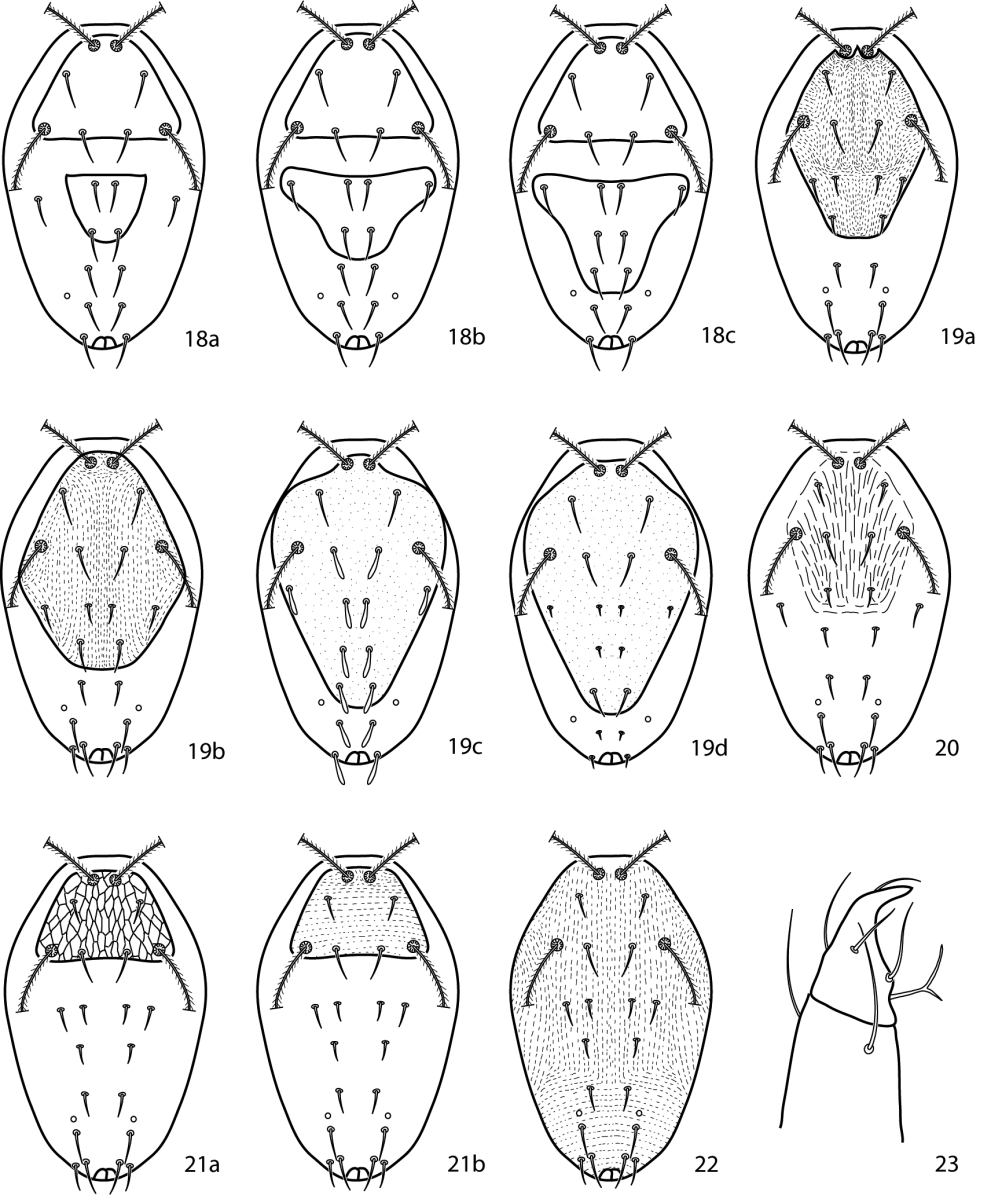
*Cunaxoides* key illustrations. See key for explanations.

### Denheyernaxoides

Taxon classificationAnimaliaTrombidiformesCunaxidae

Smiley, 1992

#### Historical review.

Canestrini (1885) described *Eupalus brevirostris*. Berlese (1894, 1897) redescribed *Eupalus brevirostris* and provided illustrations of the dorsal idiosoma, chelicera, and palp. Baker and Hoffmann (1948) proposed *Cunaxoides* as *nomen novum* as *Eupalus* was preoccupied. Smiley (1992) erected Denheyernaxoidinae and *Denheyernaxoides* for *Denheyernaxoides martini*. Lin (2001) moved transferred *Cunaxoides brevirostris* to *Denheyernaxoides* and redescribed the species based on specimens from China. Den Heyer (2009) considered Denheyernaxoidinae as a junior synonym of Cunaxoidinae. Den Heyer and Castro (2009) considered *Denheyernaxoides* to belong to Cunaxoidini. Sergeyenko (2011) reported *Denheyernaxoides brevirostris* from Ukraine and erected Denheyernaxoidini for the genus.

#### Diagnosis.

*Gnathosoma*. **Pedipalps** 3-segmented. Femurogenua at least twice as long as wide, complemented with 5 setae. Tibiotarsi at least twice as long as wide, usually complemented with 6 setae. A small apophysis occurs basally and a pointed process occurs near the terminal tip; a ridge runs between the apophysis and pointed process. **Subcapitulum** with 4 pairs of setae (*hg_1_*_–_*_4_*); setae *hg_4_* often the longest. Adoral setae absent. **Chelicera** without seta.

*Idiosoma, dorsal*. Proterosoma lacks a shield, complemented with 2 pairs of setae (*lps* and *mps*) and 2 pairs of setose sensillae (*at* and *pt*). Dorsal hysterosoma lacks a plate. Setae *c_1_*–*h_1_*, *c_2_*, and *f_2_*, *h_2_* present. Cupule *im* present laterad and posterior of *e_1_*. Integument not covered in shields or plates is striated.

*Idiosoma, ventral*. **Coxae** I–II connected by small apodemes. Coxae III–IV fused. Each coxa complemented with 2–4 setae. Genital plates each bear 4 setae (*g_1_*_–_*_4_*); 2 pairs of genital papillae visible underneath the plates. Anal plates bear 1 pair of setae; 1 pair of setae present ventrally on the integument near the anal plates. 5 pairs of setae present on the integument between the coxal and genital plates. Cupule *ih* present ventrally laterad the integumental setae associated with the anal plates. Integument not covered in shields or plates is striated. **Legs.** Femora I and II not divided. Trichobothrium on tibia IV absent. Tarsi never constricted apically so as to end in lobes. Ambulacral claws on either side of a 4-rayed empodium present.

#### Key to adult female *Denheyernaxoides*

**Table:** 

1	Coxa I with 1 sts; trochanters I–IV setal count 1-1-1-1; femora I-II setal count 2–2; gnathosoma with deep indention posterioventrally	*Denheyernaxoides martini* Smiley, 1992
–	Coxa I with 3 sts; trochanters I–IV setal count 0-0-1-0; femora I-II setal count 4–5; gnathosoma with slight indention posterioventrally	*Denheyernaxoides brevirostris* (Canestrini 1885)

### Dunaxeus

Taxon classificationAnimaliaTrombidiformesCunaxidae

Den Heyer & Castro, 2009

#### Historical review.

Den Heyer (1981b) described *Cunaxoides capensis* and *Cunaxoides elongatus*. Den Heyer and Castro (2009) erected *Dunaxeus*, transferred *Dunaxeus capensis* and *Dunaxeus elongatus* to the genus, and described *Dunaxeus duosetosus*.

#### Diagnosis.

*Gnathosoma*. **Pedipalps** 3-segmented. Femurogenua at least twice as long as wide, complemented with 5 setae. Tibiotarsi at least twice as long as wide, usually complemented with 6 setae. A small apophysis occurs basally and a pointed process occurs near the terminal tip; a ridge runs between the apophysis and pointed process. **Subcapitulum** with 4 pairs of setae (*hg_1_*_–_*_4_* and 2 pairs of adoral setae); setae *hg_4_* is often the longest. **Chelicera** without seta.

*Idiosoma, dorsal*. Proterosoma bears an ill-defined and weakly sclerotized shield which is complemented with 2 pairs of setae (*lps* and *mps*) and 2 pairs of setose sensillae (*at* and *pt*). Dorsal hysterosoma may or may not bear a plate; if a plate is present it is ill-defined and weakly sclerotized, may be complemented with a variable number of setae, and may or may not be fused with the proterosomal shield. Setae *c_1_*–*h_1_*, *c_2_*, and *h_2_* are present. Cupule *im* is present laterad and posterior of *e_1_*. The integument that is not covered in shields or plates is striated.

*Idiosoma, ventral*. **Coxae** weakly sclerotized and ill-defined; they can be recognized by possessing somewhat denser striations than the surrounding integument. Coxae I–II may be fused and may coalesce medially to form a sternal shield. Coxae III–IV fused. Each coxa complemented with 2–4 setae. Genital plates each bear 4 setae (*g_1_*_–_*_4_*); 2 pairs of genital papillae visible underneath plates. Anal plates bear 1 pair of setae; 1 pair of setae present ventrally on the integument near the anal plates. Up to 7 pairs of setae present on the integument between the coxal and genital plates. Cupule *ih* present ventrally laterad the integumental setae associated with the anal plates. Integument not covered in shields or plates is striated. **Legs.** Tarsi never constricted apically so as to end in lobes. Tibia III complemented with 5 sts (4 short, 1 long). Tibia IV complemented with 5 sts (4 short, 1 long), and lacks a trichobothrium. Ambulacral claws on either side of a 4-rayed empodium present.

#### Key to adult female *Dunaxeus*

**Table:** 

1	Basifemora IV with 1 sts	*Dunaxeus elongatus* (Den Heyer, 1981)
–	Basifemora IV with 2 sts	2
2 (1)	Famulus on tarsus I present	*Dunaxeus capensis* (Den Heyer, 1981)
–	Famulus on tarsus I absent	*Dunaxeus duosetosus* Den Heyer & Castro, 2009

### Funaxopsis

Taxon classificationAnimaliaTrombidiformesCunaxidae

Den Heyer & Castro, 2009

#### Historical review.

Den Heyer (1981b) described *Cunaxoides passerinae*, *Cunaxoides vaneedeni*, and *Cunaxoides visci*. Den Heyer and Castro (2009) erected *Funaxopsis* and transferred *Funaxopsis passerinae*, *Funaxopsis vaneedeni*, and *Funaxopsis visci* to the genus.

#### Diagnosis.

*Gnathosoma*. **Pedipalps** 3-segmented. Femurogenua at least twice as long as wide, complemented with 5 setae. Tibiotarsi at least twice as long as wide, usually complemented with 6 setae. A small apophysis occurs basally and a pointed process occurs near the terminal tip; a ridge runs between the apophysis and pointed process. **Subcapitulum** with 6 pairs of setae (*hg_1_*_–_*_4_* and 2 pairs of adoral setae); setae *hg_4_* is often longest. **Chelicera** without seta.

*Idiosoma, dorsal*. Proterosoma bears an ill-defined and weakly sclerotized shield complemented with 2 pairs of setae (*lps* and *mps*) and 2 pairs of setose sensillae (*at* and *pt*). Dorsal hysterosoma may or may not bear a plate; if plate present, it is ill-defined and weakly sclerotized, may be complemented with a variable number of setae, and may or may not be fused with the proterosomal shield. Setae *c_1_*–*h_1_*, *c_2_*, and *h_2_* present. Cupule *im* present laterad and posterior *e_1_*. Integument not covered in shields or plates striated.

*Idiosoma, ventral*. **Coxae** weakly sclerotized and ill-defined; they can be recognized by possessing somewhat denser striations than the surrounding integument. Coxae I–II may be fused and may coalesce medially to form a sternal shield. Coxae III–IV may be fused. Each coxa complemented with 2–4 setae. Genital plates each bear 4 setae (*g_1_*_–_*_4_*); 2 pairs of genital papillae visible underneath the plates. Anal plates bear 1 pair of setae; 1 pair of setae present ventrally on the integument near the anal plates. Up to 7 pairs of setae present on the integument between the coxal and genital plates. Cupule *ih* present ventrally laterad integumental setae associated with the anal plates. Integument not covered in shields or plates striated. **Legs.** Tibia III complemented with 1 bsl and 3, 4, or 5 sts. Tibia IV complemented with 3 sts (2 short, 1 long) and lacks a trichobothrium. Tarsi never constricted apically so as to end in lobes. Ambulacral claws on either side of a 4-rayed empodium present.

#### Key to adult female *Funaxopsis*

(modified from Den Heyer and Castro 2009)

**Table:** 

1	Basifemora I–IV setal formula 3-3-3-1 sts; *sci* smooth	*Funaxopsis visci* (Den Heyer, 1981)
–	Basifemora I–IV setal formula 2-2-2-0 sts; *sci* finely setose	2
2 (1)	Telofemora I–IV setal formula 4-3-1-1 sts; *h_1_* smooth	*Funaxopsis passerinae* (Den Heyer, 1981)
–	Telofemora I–IV setal formula 4-4-3-1 sts; *h_1_* finely setose	*Funaxopsis vaneedeni* (Den Heyer, 1981)

### Lupaeus

Taxon classificationAnimaliaTrombidiformesCunaxidae

Castro & Den Heyer, 2009

#### Historical review.

Berlese (1916) described *Eupalus subterraneus*. Thor and Willmann (1941) redescribed *Eupalus subterraneus*. Baker and Hoffmann (1948) erected *Cunaxoides* in place of *Eupalus* as *Eupalus* was preoccupied; they also described *Cunaxoides minutus* and redescribed and illustrated *Cunaxoides subterraneus*. Den Heyer (1979b) erected *Pulaeus*, moving those species with *f_2_* present and setae present on basifemora IV to the new genus from *Cunaxoides*; he also described *Pulaeus martini* and *Pulaeus clarae* and placed *Pulaeus* into the subfamily Cunaxoidinae. *Pulaeus platygnathus* was described by Bu and Li (1991). Corpuz-Raros (1996b) described *Pulaeus dentatus*, *Pulaeus lenis*, *Pulaeus longisetus*, *Pulaeus villacarlosae*, and *Pulaeus filipinus* from the Philippines. Hu (1997) reported *Pulaeus platygnathus* from China. Lin and Zhang (2000) reported *Pulaeus platygnathus* from China. Lin and Zhang (2003) reported *Pulaeus minutus* from China. Corpuz-Raros (2007) described *Pulaeus polilloensis* and *Pulaeus philippinensis* from the Philippines. Castro and Den Heyer (2009) erected *Lupaeus* and moved into it those species of *Pulaeus* that possess two pointed processes on the pedipalp tibiotarsus and 1 simple seta on basifemora IV; they also described *Lupaeus lectus* and *Lupaeus lobidorsalis* and provided a key to the Brazilian and South African species. Sergeyenko (2011b) described *Lupaeus valentinae*. Den Heyer et al. (2013) described *Lupaeus iranensis* and *Lupaeus sativae*.

#### Diagnosis.

**Figures 24–27.**
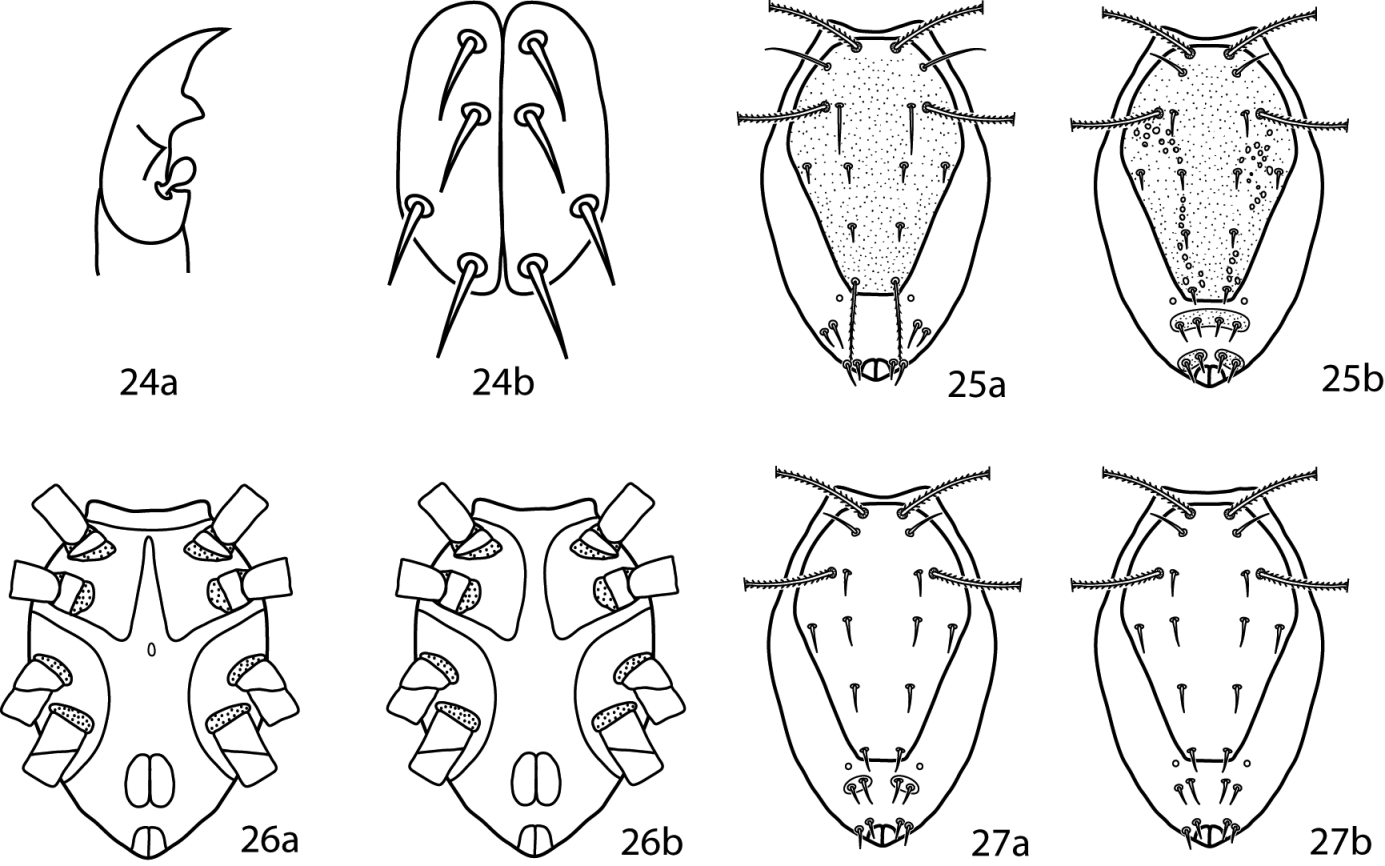
*Lupaeus* illustrations. **24a** Pedipalp tibiotarsus **24b** Genital setae not in a row, *g_3_* out of line **25–27** *Lupaeus* key illustrations. Setae and cupules removed from figures **25a, b** to increase clairity **25a** *Lupaeus longisetus*, dorsal **25b** *Lupaeus polilloensis*, dorsal **26a** Ventral, small platelet present **26b** Ventral, small platelet absent **27a** Setae *f_1_*, *f_2_* born on small platelets **27b** Setae *f_1_*, *f_2_* born on integument.

*Gnathosoma*. **Pedipalps** 3-segmented. Femurogenua at least twice as long as wide, complemented with 6 setae. Tibiotarsi at least twice as long as wide, usually complemented with 6 setae; they possess 2 or 3 pointed processes and may possess a bladder- or knob-like apophysis (Fig. 24a). **Subcapitulum** with 6 pairs of setae (*hg_1_*_–_*_4_* and 2 pairs of adoral setae); setae *hg_4_* often the longest. **Chelicera** with seta present.

*Idiosoma, dorsal*. Proterosoma bears a well-sclerotized shield complemented with 2 pairs of setae (*lps* and *mps*) and 2 pairs of setose sensillae (*at* and *pt*). Dorsal hysterosoma bears a sclerotized plate that is variable in size and fused with the proterosomal shield; it may be complemented with a variable number of setae depending on the size of the plate. Setae *c_1_*–*h_1_*, *c_2_*, *f_2_*, and *h_2_* present. Cupule *im* present laterad and posterior of *e_1_*. Integument not covered in shields or plates is striated.

*Idiosoma, ventral*. **Coxae** sclerotized and well-defined. Coxae I–II may be fused and may coalesce medially to form a sternal shield. Coxae III–IV may be fused. Each coxa complemented with 2–4 setae. Genital plates each bear 4 setae (*g_1_*_–_*_4_*). Setae *g_1,2,4_* usually occur in a straight line near the midline and setae *g_3_* occur near the edge of the genital plates (Fig. 24b). 2 pairs of genital papillae visible underneath the plates. Anal plates bear 1 pair of setae; 1 pair of setae present ventrally on the integument near the anal plates. Cupule *ih* present ventrally laterad; the integumental setae associated with the anal plates. Integument not covered in shields or plates striated. **Legs.** Tarsi never constricted apically so as to end in lobes. Trichobothrium on leg tibia IV present. Basifemora setal formula 4-6-3-1. Depression of the famulus occurs on distal half of tarsus I. Tibiae I–II possess striated blunt solenidia. Ambulacral claws rippled and occur on either side of a 4-rayed empodium.

#### Key to adult female *Lupaeus*

*Lupaeus longisetus* is known only from the male and is not included in the key. It can be recognized by the following characters: small platelet between the edges of a divided sternal shield absent, basifemora I with 3 sts, and setae *e_1_* elongate and barbed (Fig. 25a).

*Lupaeus polilloensis* is only known from the male and is not included in the key. It can be recoginized by the following characters: small platelet between the edges of a divided sternal shield absent; basifemora I–II setal formula 4-6; platelets complemented with setae *f_1_*, *f_2_* with fused medially into one plate; and the dorsal shield densely granulate (Fig. 25b).

As suggested by Den Heyer (2011b) the following species are moved to *Lupaeus* from *Pulaeus*: *Lupaeus minutus* (Baker and Hoffmann) and *Lupaeus subterraneus* (Berlese).

**Table:** 

1	Small platelet ventromedially between edges of divided sternal plate present (Fig. 26a); South Africa, Brazil	*Lupaeus martini* (Den Heyer, 1979)
–	Small platelet ventromedially between edges of divided sternal plate absent (Fig. 26b)	2
2 (1)	Basifemora I with 4 sts	3
–	Basifemora I with 5 sts; Philippines	*Lupaeus filipinus* (Corpuz-Raros, 1996)
3 (2)	Basifemora II with 4 sts; USA	*Lupaeus minutus* (Baker & Hoffmann, 1948)
–	Basifemora II with 5 sts	4
–	Basifemora II with 6 sts	7
4 (3)	Setae *f_1_* shorter than *c_1_*; Philippines	*Lupaeus lenis* (Corpuz-Raros, 1996)
–	Setae *f_1_* the same length as *c_1_*	*Lupaeus lectus* Castro & Den Heyer, 2009
–	Setae *f_1_* longer than *c_1_*, usually by at least 1.5 times	5
5 (4)	Genua I with 9 total simple setae and solenidia; Philippines	*Lupaeus dentatus* (Corpuz-Raros, 1996)
–	Genua I with 7 total simple setae and solenidia	6
6 (5)	Setae *c_1_*–*e_1_* equal in length; Brazil	*Lupaeus lobidorsalis* Castro & Den Heyer, 2009
–	Setae *e_1_* one-fourth longer than *c_1_*, *d_1_*; Italy, USA	*Lupaeus subterraneus* (Berlese, 1916)
7 (3)	Setae *f_1_*, *f_2_* on platelets, which may be separate or fused medially (Fig. 27a)	8
–	Setae *f_1_*, *f_2_* on integeument (Fig. 27b)	11
8 (7)	Tibia II with 1 s, 5 sts	9
–	Tibia II with 2 s (1 asl, 1 bsl), 5 sts; Ukraine	*Lupaeus valentinae* Sergeyenko, 2011
9 (8)	Pedipalp tibiotarsus with 4 sts; Philippines	*Lupaeus villacarlosae* (Corpuz-Raros, 1996)
–	Pedipalp tibiotarsus with 5 sts	10
10 (9)	Tarsus I with 3 asl, 2 terminal solenidion, 1 fam, 20 or 21 sts; tarsus IV with 14 sts	*Lupaeus iranensis* Den Heyer, 2013
–	Tarsus I with 3 asl, 1 dorsodistal solenidion, 1 terminal solenidion, 1 fam, 22 sts; tarsus IV with 16 sts	*Lupaeus sativae* Den Heyer, 2013
11 (7)	Cheliceral seta not as long as width of cheliceral digit; China	*Lupaeus platygnathus* (Bu & Li, 1991)
–	Cheliceral seta longer than width of cheliceral digit; South Africa, Brazil	*Lupaeus clarae* (Den Heyer, 1979)

### Neocunaxoides

Taxon classificationAnimaliaTrombidiformesCunaxidae

Smiley, 1975

#### Historical review.

Baker and Hoffmann (1948) described *Cunaxoides andrei*. Smiley (1975) erected *Neocunaxoides* and moved *Neocunaxoides andrei* to the genus. Gupta and Chattopadhyay (1978) described *Neocunaxoides biswasi* from bird nests in Bengal, India. Den Heyer (1978c) placed *Neocunaxoides* in the subfamily Cunaxoidinae. Kuznetzov and Livshitz (1979) reported *Cunaxoides andrei* from Russia, having either disagreed with or been unaware of Smiley’s 1975 publication. Den Heyer (1980a) described *Neocunaxoides lajumensis*, *Neocunaxoides rykei*, and *Neocunaxoides zuluensis* from South Africa. Tseng (1980) reported and figured *Neocunaxoides andrei* and *Neocunaxoides whartoni* from Taiwan. Michocka (1987) reported *Neocunaxoides andrei* from Poland. Inayatullah and Shahid (1989) described *Neocunaxoides dilato* and *Neocunaxoides kalamiensis*. *Neocunaxoides cerasoides* was described by Gupta (1991). Smiley (1992) synonymized *Scutopalus* with *Neocunaxoides* and moved *Cunaxoides trepidus* to *Neocunaxoides*. Corpuz-Raros (1996c) described *Neocunaxoides grandis* and *Neocunaxoides mahabaeus*. Hu (1997) reported *Neocunaxoides andrei* from China. Lin, Zhang, and Ji (2001) described *Neocunaxoides boltoides* and *Neocunaxoides fani* and later (2003) described *Neocunaxoides ovatus*. Fawzy (2007) described *Neocunaxoides metwallyi*. Corpuz-Raros and Gruèzo (2007) described *Neocunaxoides ornatus*. Castro and Den Heyer (2009) moved *Pulaeus trepidus* (=*Neocunaxoides trepidus*) to *Scutopalus*.

#### Diagnosis.

*Gnathosoma*. **Pedipalps** 3-segmented. Femurogenua at least twice as long as wide, complemented with 6 setae. Tibiotarsi at least twice as long as wide and usually complemented with 6 setae. Tibiotarsi possess two or three knob-like apophyses, a single spur, or sometimes a flange-like seta. **Subcapitulum** with 6 pairs of setae (*hg_1_*_–_*_4_* and 2 pairs of adoral setae); setae *hg_4_* often the longest. **Chelicera** with seta present.

*Idiosoma, dorsal*. Proterosoma bears a well-sclerotized shield which is complemented with 2 pairs of setae (*lps* and *mps*) and 2 pairs of setose sensillae (*at* and *pt*). Dorsal hysterosoma bears a sclerotized plate which is variable in size and fused with the proterosomal shield; it may be complemented with a variable number of setae depending on the size of the plate. Setae *c_1_*–*h_1_*, *c_2_*, and *h_2_* present. Setae *f_2_* absent. Cupule *im* present laterad and posterior of *e_1_*. The integument not covered in shields or plates is striated.

*Idiosoma, ventral*. **Coxae** sclerotized and well-defined. Coxae I–II may be fused and may coalesce medially for form a sternal shield. Coxae III–IV may be fused. Each coxa complemented with 2–4 setae. Genital plates each bear 4 setae (*g_1_*_–_*_4_*), which are usually in a straight now; 2 pairs of genital papillae visible underneath the plates. Anal plates bear one pair of setae; one pair of setae is present ventrally on the integument near the anal plates. Cupule *ih* present ventrally laterad the integumental setae associated with the anal plates. Integument not covered in shields or plates is striated. **Legs.** Tarsi never constricted apically so as to end in lobes. Trichobothrium on leg tibia IV present. Basifemora setal formula 3-5-2-0. Ambulacral claws rippled and occur on either side of a 4-rayed empodium.

#### Key to adult female *Neocunaxoides*

*Cunaxoides philippinensis* (Corpuz-Raros, 2007) is regarded as belonging to *Neocunaxoides* because it has 6 seatae on the femurogenu and lacks setae *f_2_*. *Neocunaxoides makapalus*, *Neocunaxoides philippinensis* (Corpuz-Raros, 1996c), *Neocunaxoides unguianalis*, and *Neocunaxoides rugosus* are regarded as belonging to *Scutopalus* as they possess 5 sts on pedipalp femurogenu and extensive dorsal shields. They have therefore not been included in the following key.

*Neocunaxoides biramus* is not included in the key because it is only known from the male. It can be distinguished from all other *Neocunaxoides*, and indeed all described cunaxids, by the presence of a branched *sci* and 4 teeth on the lateral lips of the hypostome.

*Neocunaxoides metwallyi* is not included in the key as, despite the best efforts of the authors and the University of Arkansas Interlibrary Loan Department, the description could not be obtained.

We agree with and follow Castro and Den Heyer (2009) and Den Heyer and Castro (2009) in regarding *Scutopalus* as a valid and separate genus.

**Table:** 

1	Coxae I–II fused medially to form sternal shield (Figs 28a–d)	2
–	Coxae I–II not fused medially (may be connected anteromedially) (Figs 29a–d)	6
2 (1)	Posterior edge of coxae IV extending beyond anterior edge of genital plates (Figs 28a, b)	3
–	Posterior edge of coxae IV not extending beyond anterior edge of genital plates (Figs 28c, d)	5
3 (2)	Small platelet anteriomedially of genital plates present (Fig. 28a)	*Neocunaxoides fani* Lin, Zhang & Ji, 2001
–	Small platelet anteriomedially of genital plates absent (Fig. 28b)	4
4 (3)	Solid or broken band of papillae on ventral subcapitulum present (Fig. 30a); subcapitulum longer, length: width 1.75:1	*Neocunaxoides zuluensis* Den Heyer, 1980
–	Solid or broken band of papillae on ventral subcapitulum absent (Fig. 30b); subcapitulum shorter, length: width 1.25:1	*Neocunaxoides lajumensis* Den Heyer, 1980
5 (2)	Hysterosomal plate present, fused with proterosomal shield, and bearing *c_1_*–*e_1_*, *c_2_*; small platelet anteriomedially of genital plates present (Fig. 28c)	*Neocunaxoides boltoides* Lin, Zhang & Ji, 2001
–	Hysterosomal plate absent; small platelet anteriomedially of genital plates absent (Fig. 28d)	*Neocunaxoides philippinensis* (Corpuz-Raros, 2007)
6 (1)	Median platelet between coxae II present (Figs 29a–c)	7
–	Median platelet between coxae II absent (Fig. 29d)	13
7 (6)	Basifemora V with 1 sts	8
–	Basifemora V with 0 sts	11
8 (7)	Basifemora I with 2 sts	*Neocunaxoides biswasi* Gupta & Chattopadhyay, 1978
–	Basifemora I with 3 sts	9
9 (8)	All setae on pedipalp of normal length, none extremely long	10
–	2 setae on pedipalp femurogenu extremely long, nearly as long as segment; 1 distal pedipalp tibiotarsal setalong, longer than segment (Fig. 31)	*Neocunaxoides mahabaeus* Corpuz-Raros, 1996
10 (9)	Basal subcapitular polygonal pattern elongate (Fig. 32a); foveolae on dorsal shield present (Fig. 33a)	*Neocunaxoides ornatus* Corpuz-Raros & Gruèzo, 2007
–	Basal subcapitular polygonal pattern not elongate (Fig. 32b); foveolae on dorsal shield absent (Fig. 33b)	*Neocunaxoides grandis* Corpuz-Raros, 1996
11 (7)	Small platelet anteriomedially of genital plates present (Fig. 29a)	*Neocunaxoides ovatus* Lin, Zhang & Ji, 2003
–	Small platelet anteriomedially of genital plates absent (Fig. 29b, c)	12
12 (11)	Coxae I connected anteromedially (Fig. 29b); mushroom-shaped seta on pedipalp tibiotarsi absent	*Neocunaxoides rykei* Den Heyer, 1980
–	Coxae I not connected anteromedially (Fig. 29c); mushroom-shaped seta on pedipalp tibiotarsi present (Fig. 34)	*Neocunaxoides andrei* (Baker & Hoffmann, 1948)
13 (6)	Femora I (basifemora I + telofemora I) with 6 setae	*Neocunaxoides cerasoides* Inayatullah & Shahid, 1989
–	Femora I (basifemora I + telofemora I) with 9 setae	15
14 (13)	Coxae I-IV setal formula 2-3-3-1; combined femora (basifemora + telofemora) II-IV setal formula 11-7-5	*Neocunaxoides dilato* Inayatullah & Shahid, 1989
–	Coxae I-IV setal formula 2-2-3-2; combined femora (basifemora + telofemora) II-IV setal formula 10-7-4	*Neocunaxoides kalamiensis* Inayatullah & Shahid, 1989

**Figures 28–34.**
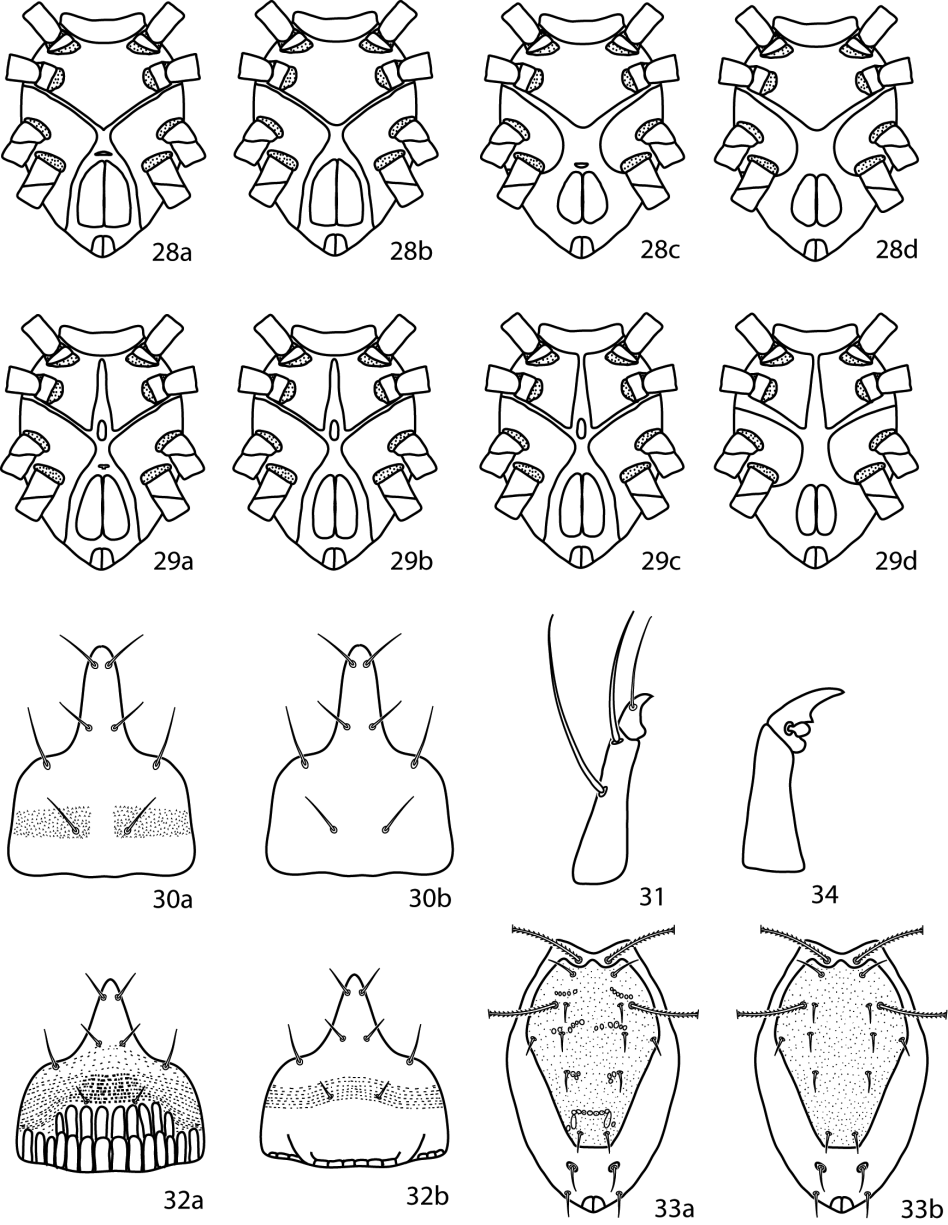
*Neocunaxoides* key illustrations. See key for explanations of figures.

### Paracunaxoides

Taxon classificationAnimaliaTrombidiformesCunaxidae

Smiley, 1992

#### Historical review.

Smiley (1992) erected *Paracunaxoides* for a single species, *Paracunaxoides newzealandicus*. Den Heyer and Castro (2009) state that *Paracunaxoides* could be synonomyous with *Cunaxoides* but refrained from sinking the genus as they had not examined the type material.

#### Diagnosis.

*Gnathosoma*. **Pedipalps** 3-segmented. Femerogenu complimented with 5 setae. Tibiotarsi at least twice as long as wide and complemented with 3 setae. Tibiotarsi possess a stout, spine-like apophysis. **Subcapitulum** with 4 pairs of setae (*hg_1_*_–_*_4_*); setae *hg_2–4_* subequal. Adoral setae absent.

*Idiosoma, dorsal*. Proterosoma complemented with 2 pairs of setae (*lps* and *mps*) and 2 pairs of setose sensillae (*at* and *pt*). A pair of oval shields formed by flat, bacillus-like striae present between the sensillae. Setae *c_1_*–*h_1_*, *c_2_*, and *h_2_* present. Setae *f_2_* absent. Integument not covered in shields or plates is striated.

*Idiosoma, ventral*. **Coxae** sclerotized and well-defined. Coxae I–II thinly connected. Coxae III–IV more broadly connected. Genital plates each bear 4 setae (*g_1_*_–_*_4_*); 2 pairs of genital papillae visible underneath the plates. Anal plates bear 1 pair of setae; 1 pair of setae present ventrally on the integument near the anal plates. Integument not covered in shields or plates is striated. **Legs.** Trichobothrium on tibia IV present.

### Pulaeus

Taxon classificationAnimaliaTrombidiformesCunaxidae

Den Heyer, 1978

#### Historical review.

Ewing (1909) described the first species of *Pulaeus* as *Eupalus pectinatus*. Berlese (1916) described *Eupalus sternalis*. Baker and Hoffmann (1948) proposed *Cunaxoides* to replace *Eupalus* as the name was preoccupied; described *Cunaxoides patzcuarensis*, *Cunaxoides whartoni*, and *Cunaxoides americanus*; and synonymized *Cunaxoides sternalis* with *Cunaxoides pectinatus*. They also redescribed and illustrated *Cunaxoides pectinatus*. Muma (1960) described *Cunaxoides pectinellus*. Shiba (1978) described *Cunaxoides neopectinatus*, *Cunaxoides parapatzuarensis*, and *Cunaxoides pseudominutus*. Chaudhri, Akbar, and Rasool (1979) described *Neocunaxoides krama*. Kuznetzov and Livshitz (1979) reported *Cunaxoides pectinatus* and *Cunaxoides americanus* from Russia. Den Heyer (1979b) erected *Pulaeus* and moved the previously mentioned species into the new genus; he also redescribed *Pulaeus pectinatus* and described *Pulaeus glebulentus*. *Neocunaxoides cinctus* was described by Chaudhri (1980). Den Heyer (1981c) confirmed the synonymy of *Pulaeus sternalis* with *Pulaeus pectinatus*, and synonymized *Cunaxoides pectinellus* with *Pulaeus pectinatus*; he also described *Pulaeus franciscae* and placed *Pulaeus* within Cunaxoidinae, tribe Pulaeini. El-Bishlawy and Rakha (1983) described *Pulaeus zaherii* from Egypt. Liang (1983) reported *Pulaeus pseudominutus* from China. *Pulaeus musci* was described by Liang (1985). Zaher and El-Bishlawy (1986) described *Pulaeus niloticus*. Bu and Li (1987b) described *Pulaeus longignathos* and *Pulaeus chongqingensis*. Muhammad and Chaudhri (1990) described *Pulaeus desitis*, *Pulaeus ferventis*, *Pulaeus osculum*, and *Pulaeus verno* from Pakistan. *Pulaeus ardeola* was described by Barilo (1991). Muhammad and Chaudhri (1991a) described *Pulaeus camar*, *Pulaeus erinaceus*, *Pulaeus galumma*, *Pulaeus haurio*, *Pulaeus silicula*, and *Pulaeus stultus* from Pakistan. Smiley (1992) synonymized *Pulaeus niloticus* with *Pulaeus subterraneus* and provided a key to known world species; he also transferred *Cunaxoides neopectinatus* to *Neocunaxoides*. Li et al. (1992) recorded *Pulaeus glebulentus* from Chongqing, China. Corpuz-Raros (1996b) described two species, *Pulaeus payatopalpus* and *Pulaeus rimandoi*, from the Philippines. Lin and Zhang (2000) reported *Neocunaxoides neopectinatus*,﻿ *Pulaeus longignathos*, *Pulaeus musci*, and *Pulaeus pseudominutus* from China. Lin et al. (2003) reported *Pulaeus minutus* from China. Bashir, Afzal, and Akbar (2005) described *Pulaeus punctatus*. Bashir and Afzal (2006b) described *Pulaeus anjumi*. Corpuz-Raros (2007) also described *Pulaeus cebuensis*, *Pulaeus palawanensis*, and *Pulaeus samarensis*. Castro and Den Heyer (2009) split *Lupaeus* from *Pulaeus* and described two new species: *Pulaeus myrtaceus* and *Pulaeus quadrisolenidius*; they also synonymized *Pulaeus longignathos* with *Neocunaxoides krama* and transferred *Neocunaxoides krama* to *Pulaeus*. Bashir and Afzal (2009) described *Pulaeus akbari*, *Pulaeus banksi*, and *Pulaeus walii*. Lin and Zhang (2010) argue that the “original species name *longignathos* [as in *Pulaeus longignathos*] is the correct form in Greek. Some authors emended it to the Latinized form *longignathus* (e.g. Castro and Den Heyer, 2009: 2).” The spelling *longignathos* is followed here. Sergeyenko (2011b) described *Pulaeus leonidi*, *Pulaeus maslovi*, and *Pulaeus semistriatus* and synonymized *Pulaeus longignathos* and *Pulaeus chongqingensis* with *Pulaeus krama* as he considered them to be male and female of that species, respectively. Den Heyer et al. (2013) described *Pulaeus razanensis*.

#### Diagnosis.

*Gnathosoma*. **Pedipalps** 3-segmented. Femurogenua at least twice as long as wide, complemented with 6 setae. Tibiotarsi at least twice as long as wide, usually complemented with 6 setae, 1 pointed process, and may possess a bladder- or knob-like apophysis (Fig. 39a–c). **Subcapitulum** with 6 pairs of setae (*hg_1_*_–_*_4_* and 2 pairs of adoral setae); setae *hg_4_* often the longest. **Chelicera** with seta present.

**Figures 35–39.**
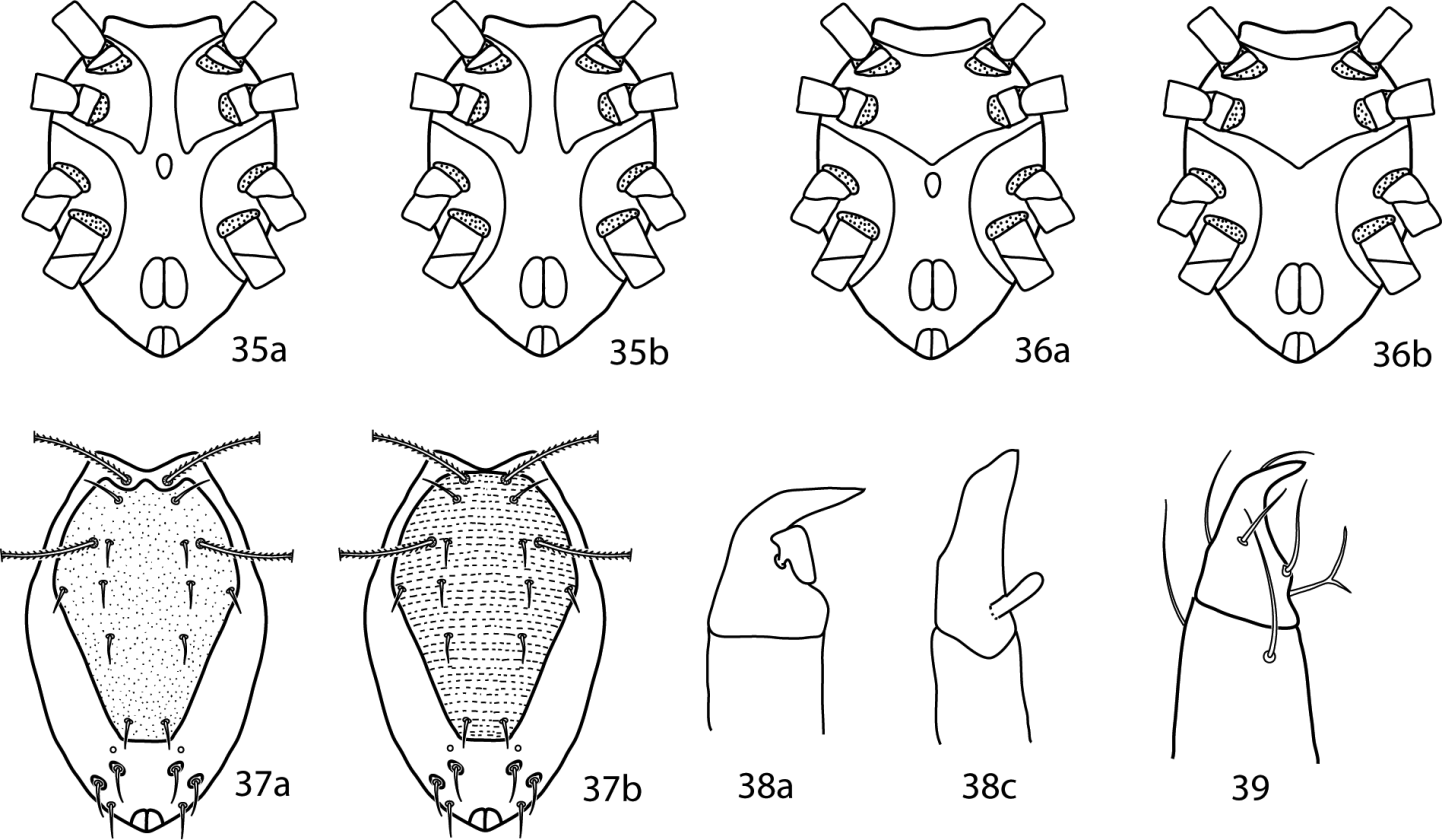
*Pulaeus* illustrations. **35** Genital setae in a row **36–39** *Pulaeus* key illustrations **36, 37** Venter, setae removed for clairity **36a** Coxae I–II not coalesced medially, median platelet present **36b** Coxae I–II not coalesced medially, median platelet absent **37a** Coxae I–II coalesced medially, median platelet present **37b** Coxae I–II coalesced medially, median platelet absent **38a** Dorsal shield with punctures **38b** Dorsal shield with broken striae **39a–c** Pedipalp tibiotarsus **39a** Tibiotarsus with elongate apophysis **39b** Tibiotarsus with flat apophysis **39c** Tibiotarsus with flange-like apophysis.

*Idiosoma, dorsal*. Proterosoma bears a well-sclerotized shield, complemented with 2 pairs of setae (*lps* and *mps*) and 2 pairs of setose sensillae (*at* and *pt*). Dorsal hysterosoma bears a sclerotized plate which is variable in size and fused with the proterosomal shield; it may be complemented with a variable number of setae depending on the size of the plate. Setae *c_1_*–*h_1_*, *c_2_*, *f_2_*, and *h_2_* and present. Cupule *im* present laterad and posterior of *e_1_*. Integument not covered in shields or plates striated.

*Idiosoma, ventral*. **Coxae** sclerotized and well-defined. Coxae I–II may be fused and may coalesce medially to form a sternal shield. Coxae III–IV may be fused. Each coxa complemented with 2–4 setae. Genital plates each bear 4 setae (*g_1_*_–_*_4_*), which are usually in a straight row; 2 pairs of genital papillae visible underneath the plates. Anal plates bear one pair of setae; 1 pair of setae present ventrally on the integument near the anal plates. Cupule *ih* present ventrally laterad the integumental setae associated with the anal plates. The integument not covered in shields or plates striated. **Legs.** Tarsi never constricted apically so as to end in lobes. Trichobothrium on leg tibia IV present. Depression of the famulus occurs on proximal half of tarsus I. Tibiae I–II possess non-striated blunt solenidia. Ambulacral claws rippled and occur on either side of a 4-rayed empodium.

#### Key to adult female *Pulaeus*

*Pulaeus ardeola* was not included in the key because the original text is in Cyrillic script and the illustrations do not provide enough characters to differentiate it from other species. *Neocunaxoides cinctus* is moved from *Neocunaxoides* to *Pulaeus* based on features given in the original description, namely that *f_2_* is present and basifemora IV are complemented with 2 sts.

The following were species assigned to *Pulaeus* before *Lupaeus* was erected. The characters that divide the two genera are not given in the original species descriptions and types have not been viewed. These indeterminable species are therefore not included in either generic key, but instead characters are given for each species that will serve to identify them.

*Pulaeus parapatzuarensis* (Shiba, 1978) – This species has a divided sternal plate, lacks a sclerotized area anterior to the genital plates, and does not have *f_1,2_* located on platelets. In addition it has 6 pairs of setae on the integument between coxal and genital plates.

*Pulaeus patzcuarensis* (Baker & Hoffmann, 1948) – This species can be recognized by the sternal plates being connected anteriorly and divided in a v-shape posteriorly.

*Pulaeus pseudominutus* (Shiba, 1978) – Setae *e_1_* being 3 times the length of *c_1_* and *d_1_* distinguishes this species.

*Pulaeus payatopalpus* (Corpuz-Raros, 1996) – The hypostome is 2/3 the length of the gnathosoma and the pedipalps are extremely long and slender, at least 8 times longer than wide. In addition the tibiotarsus is complemented with a seta that is longer than the segment.

*Pulaeus zaherii* (El-Bishlawy & Rakha, 1983) – This species can be recognized by the divided sternal plates, *f_1_* being 4/5 the length of *e_1_*, and *f_1_* being ½ the length of *f_2_*.

**Table:** 

1	Sternal plate divided medially (Fig. 35a, b)	2
–	Sternal plate not divided medially (Fig. 36a, b)	23
2 (1)	Median platelet between coxae II–III present (Fig. 35a)	3
–	Median platelet between coxae II–III absent (Fig. 35b)	7
3 (2)	Dorsal shield with surface smooth anteriorly and broken striae or lobes posteriorly; Ukraine	*Pulaeus semistriatus* Sergeyenko, 2011
–	Dorsal shield with surface patterned (broken striae/lobes or dotted) on entire surface	4
4 (3)	Dorsal shield patterend with dots; Pakistan	*Pulaeus punctatus* Bashir, Afzal & Akbar, 2005
–	Dorsal shield patterned with broken striae/lobes	5
5 (4)	Genua II with solenidia present	6
–	Genua II with solenidia absent; Pakistan	*Pulaeus banksi* Bashir & Afzal, 2009
6 (5)	Genua II with 1 asl, 5 sts; genua III with 2 asl, 5 sts; South Africa	*Pulaeus glebulentus* Den Heyer, 1979
–	Genua II with 2 asl, 4 sts; genua III with 1 asl, 5 sts; Iran	*Pulaeus razanensis* Den Heyer, 2013
7 (2)	Setae *f_1_* and *f_2_* located on sclerotized platelets or shields	8
–	Setae *f_1_* and *f_2_* not located on sclerotized platelets or shields	20
8 (7)	Pedipalp femurogenu at least 6 times as long as wide; Philippines	*Pulaeus rimandoi* Corpuz-Raros, 1996
–	Pedipalp femurogenu at most 4 times as long as wide	9
9 (8)	Genua II with 0 solenidia; Pakistan	10
–	Genua II with 1 solenidion	12
–	Genua II with 2 solenidia; Philippines	*Pulaeus samarensis* Corpuz-Raros, 2007
–	Genua II with 3 solenidia	17
–	Genua II with 4 solenidia	19
10 (9)	Genua I wth 2 bsl, 6 sts; tibia I with 1 bsl, 6 sts; Pakistan	*Pulaeus ferventis* Muhammad & Chaudhri, 1990
–	Genua I with 2 asl, 3 bsl, 3 sts; tibia I with 1 bsl, 7 sts; Pakistan	*Pulaeus erinaceus* Muhammad & Chaudhri, 1991
–	Genua I with 3 bsl, 6 sts; tibia I with 1 asl, 1 bsl, 6 sts; Pakistan	*Pulaeus galumma* Muhammad & Chaudhri, 1991
–	Genua I with 4 asl, 4 sts; tibia I with 1 asl, 6 sts; Pakistan	*Pulaeus walii* Bashir & Afzal, 2009
–	Genua I with 5 bsl, 4 sts; tibia I with 1 bsl, 6 sts; Pakistan	11
11 (10)	Basifemora I–IV setal formula 5-5-4-3; Pakistan	P. silicula Muhammad & Chaudhri, 1991
–	Basifemora I–IV setal formula 4-6-3-1; Pakistan	*Pulaeus stultus* Muhammad & Chaudhri, 1991
12 (9)	Basifemora I with solenidion present; telofemora I–IV setal formula 5-5-3-2; Pakistan	*Pulaeus camar* Muhammad & Chaudhri, 1991
–	Basifemora I with solenidion absent; telofemora I–IV setal formula not as above	13
13 (12)	Basifemora II with 5 (rarely 4) sts; Ukraine	*Pulaeus leonidi* Sergeyenko, 2011
–	Basifemora II with 6 sts	14
14 (13)	Genua II with solenidia present	15
–	Genua II with solenidia absent; Pakistan	*Pulaeus akbari* Bashir & Afzal, 2009
15 (14)	Genua II with 1 asl, 5 sts; Ukraine	*Pulaeus maslovi* Sergeyenko, 2011
–	Genua II with 1 bsl, 6 sts	16
16(15)	Genua III–IV setal formula 5 sts–5 sts; Pakistan	*Pulaeus osculum* Muhammad & Chaudhri, 1990
–	Genua III–IV setal formula 5 sts–6 sts; Pakistan	*Pulaeus haurio* Muhammad & Chaudhri, 1991
–	Genua III–IV setal formula 1 bsl, 4 sts–2 bsl, 4 sts; Pakistan	*Pulaeus verno* Muhammad & Chaudhri, 1990
17 (9)	Setae *f_1_* and *h_1_* approximately equal in length	18
–	Setae *f_1_* approximately half the length as *h_1_*; China	*Pulaeus musci* Liang, 1985
18 (17)	Coxa IV with 2 sts; basifemora IV with 2 sts; Brazil	*Pulaeus myrtaceus* Castro & Den Heyer, 2009
–	Coxa IV with 3 sts; basifemora IV with 1 sts; Pakistan	*Pulaeus anjumi* Bashir & Afzal, 2006
19 (9)	Dorsal shield with punctuations (Fig. 37a); Brazil	*Pulaeus quadrisolenidius* Castro & Den Heyer, 2009
–	Dorsal shield with flat broken striae (Fig. 37b); USA	*Pulaeus whartoni* (Baker & Hoffmann, 1948
20 (7)	4 pairs of setae on integument between coxal and genital plates	*Pulaeus cinctus* (Chaudhri, 1980)
–	5 pairs of setae on integument between coxal and genital plates	21
–	6 pairs of setae on integument between coxal and genital plates	22
21 (20)	Coxae II with 2 sts; telofemora II with 5 sts; Pakistan	*Pulaeus desitis* Muhammad & Chaudhri, 1990
–	Coxae II with 2 sts; telofemora II with 4 sts; Philippines	*Pulaeus palawanensis* Corpuz-Raros, 2007
22 (20)	Sensillum *at* approximately as long as *sce*; setae *f_1_* approximately equal in length to *h_1_*	*Pulaeus cebuensis* Corpuz-Raros, 2007
–	Sensillum *at* longer than *sce*; setae *f_1_* approximately 1.25 the length of *h_1_*	*Pulaeus franciscae* Den Heyer, 1981
23 (1)	Ventral medial platelet present (Fig. 36a); dorsum punctuate (Fig. 37a); pedipalpal tibiotarsus with truncate, flange-like apophysis (Fig. 38a); USA	*Pulaeus pectinatus* Den Heyer, 1979
–	Ventral medial platelet absent (Fig. 36b); dorsum striated (Fig. 37b); pedipalpal tibiotarsus with elongate apophysis (Fig. 38b)	24
24 (23)	Posterior pedipalpal tibiotarsal seta bifurcate (Fig. 39)	*Pulaeus neopectinatus* (Shiba, 1978)
–	Posterior pedipalpal tibiotarsal seta not bifurcate	25
25 (24)	Pedipalp femurogenua at most 4 times as long as wide; setae *f_1_* and *f_2_* approximately equal in length; USA	*Pulaeus americanus* (Baker & Hoffmann, 1948)
–	Pedipalp femurogenua at least 6 times as long as wide; setae *f_1_* ¼ longer than *f_2_*; Pakistan	*Pulaeus krama* (Chaudhri, Akbar & Rasool 1979)

### Qunaxella

Taxon classificationAnimaliaTrombidiformesCunaxidae

Den Heyer & Castro, 2009

#### Historical review.

Den Heyer and Castro (2009) erected *Qunaxella* for a single species, *Qunaxella triasetosa*.

#### Diagnosis.

*Gnathosoma*. **Pedipalps** 3-segmented. Femurogenu complimented with 5 sts. Tibiotarsi at least twice as long as wide and complemented with 5 sts, 1 asl. **Subcapitulum** with 6 pairs of setae (*hg_1_*_–_*_4_* and 2 pairs of adoral setae).

*Idiosoma, dorsal*. Proterosoma with weakly defined shield present which is complemented with 2 pairs of setae (*lps* and *mps*) and 2 pairs of setose sensillae (*at* and *pt*). Dorsal hysterosoma lacks a plate. Setae *c_1_*–*h_1_*, *c_2_*, and *h_2_* present. Setae *c_1_*–*f_1_* finely setose and *c_2_*, *h_1_*, and *h_2_* smooth. Setae *f_2_* absent. Integument not covered in shields or plates striated.

*Idiosoma, ventral*. **Coxae** weakly sclerotized and ill-defined. Coxae I–II fused. Coxae III–IV fused. Genital plates each bear 4 setae (*g_1_*_–_*_4_*); 2 pairs of genital papillae visible underneath the plates. Integument not covered in shields or plates striated. **Legs.** Basifemora I–IV setal formula 3-4-2-0 sts. Telofemora I–IV setal formula 4-4-3-3. Tibiae III with 1 bsl, 5 sts. Tibiae IV with 5 sts (4 short, 1 long).

### Scutopalus

Taxon classificationAnimaliaTrombidiformesCunaxidae

Den Heyer, 1979

#### Historical review.

Den Heyer (1979c) erected *Scutopalus* for *Scutopalus arboreus* and *Scutopalus latisetosus*. Shiba (1978) described *Cunaxoides clavatus*. Kuznetzov and Livshitz (1979) described *Cunaxoides trepidus*. Tseng (1980) described *Neocunaxoides osseus* and *Neocunaxoides unguianalis*. Gupta and Ghosh (1980) described *Neocunaxoides pradhani*. Smiley (1992) synonymized *Scutopalus* with *Neocunaxoides* and transferred *Cunaxoides trepidus* to *Neocunaxoides*. Corpuz-Raros (1996c) described *Neocunaxoides makapalus*, *Neocunaxoides philippinensis*, and *Neocunaxoides rugosus*. Lin and Zhang (2000) recorded *Neocunaxoides clavatus* from tea in China. Sionti and Papadoulis (2003) described *Neocunaxoides abiesae* and *Neocunaxoides smolikensis*. Bashir and Afzal (2004b) described *Neocunaxoides gilbertoi*. Castro and Den Heyer (2009) transferred *Pulaeus trepidus* (=*Neounaxoides trepidus*) to *Scutopalus*. Rocha et al. (2013) described *Scutopalus tomentosus* and transferred *Neocunaxoides makapalus*, *Neocunaxoides philippinensis*, *Neocunaxoides rugosus*, and *Neocunaxoides unguianalis* to *Scutopalus*.

#### Diagnosis.

*Gnathosoma*. **Pedipalps** 3-segmented. Femurogenu complimented with 5 sts. Tibiotarsi at least twice as long as wide and complemented with 5 sts, 1 asl. Subterminal pointed process on pedipalp tibiotarsal claw absent; small teeth on pedipalp tibiotarsal claw absent. **Subcapitulum** with 6 pairs of setae (*hg_1_*_–_*_4_* and 2 pairs of adoral setae). **Chelicera** without seta.

*Idiosoma, dorsal*. Proterosoma with a well-defined shield present, complemented with 2 pairs of setae (*lps* and *mps*) and 2 pairs of setose sensillae (*at* and *pt*). Dorsal hysterosoma with a well-defined plate fused to the proterosomal plate. Small platelets may be present laterad and posterior to the dorsal shield. Setae *c_1_*–*h_1_*, *c_2_*, and *h_2_* present. Setae *f_2_* absent. Integument not covered in shields or plates striated.

*Idiosoma, ventral*. **Coxae** well-sclerotized. Coxae I–II fused medially. Coxae III–IV fused. Genital plates each bear 4 setae (*g_1_*_–_*_4_*); 2 pairs of genital papillae visible underneath the plates. A small platelet may be present laterad the genital plate. Integument not covered in shields or plates striated. **Legs.** Basifemora I–IV setal formula 3-4-2-0 sts. Telofemora I–IV setal formula 5-5-4-3. Tibiae III with 1 bsl, 5 sts. Tibiae IV with 5 sts (4 short, 1 long).

#### Key to female *Scutopalus*

(modified from Rocha et al. 2013).

As suggested by Den Heyer (2011b) *Neocunaxoides pradhani* (Gupta and Ghosh 1980) and *Neocunaxoides gilbertoi* (Bashir and Afzal 2004) are transferred to *Scutopalus* as they posses 5 setae on the femurogenu instead of 6 as in *Neocunaxoides* and have well-demarcated plates.

**Table:** 

1	Coxae I–II faintly or totally divided (Fig. 40a, b)	2
–	Coxae I–II fused medially (Fig. 40c)	7
2 (1)	Coxae I–II faintly divided (Fig. 40a)	3
–	Coxae I–II totally divided (Fig. 40b)	4
3 (2)	Sternal shield bearing 6 pairs of setae; setae *c_2_* and *mps* simple; coxae II with 2 setae; basifemora I–IV setal formula 3-3-2-0; Greece	*Scutopalus abiesae* Sionti & Papadoulis, 2003
–	Sternal shield bearing 5 pairs of setae; setae *c_2_* and *mps* setose; coxae II with 1 setae; basifemora I–IV setal formula 2-2-2-1; South Africa	*Scutopalus arboreus* Den Heyer, 1979
4 (2)	At least 2 pairs of thick rod-like setae on the dorsum (Fig. 41); India	*Scutopalus pradhani* (Gupta & Ghosh, 1980)
–	Rod-like setae on dorsal shield absent	5
5 (4)	Coxae II with 2 sts	6
–	Coxae II with 3 sts; Pakistan	*Scutopalus gilbertoi* (Bashir & Afzal, 2004)
6 (4)	Setae *f_1_* and *h_1_* on small platelets; ratio *c_1_*: *c_2_* 2:1; genua I with 4 asl, 5 sts; genua II with 2 asl, 5 sts; South Africa	*Scutopalus latisetosus* Den Heyer, 1979
–	Setae *f_1_* and *h_1_* on integument; ratio *c_1_*: *c_2_* 1:1; genua I with 3 asl, 5 sts; genua II with 1 asl, 5 sts; Greece	*Scutopalus smolikensis* Sionti & Papadoulis, 2003
7 (1)	Dorsal shield smooth and/or punctate (Fig. 42a)	8
–	Dorsal shield sparse granulate, rugose, or reticulate (Fig. 42b–d)	12
8 (7)	Coxae II and IV with 2 setae	9
–	Coxae II and IV with 3 setae	11
9 (8)	Setae *mps*, *c_1_*, *c_2_*, *d_1_*, *e_1_*, *f_1_* clavate (Fig. 43); a small subscutum situated posterior to the dorsal shield present; Malaysia	*Scutopalus clavatus* (Shiba, 1978)
–	Setae *mps*, *c_1_*, *c_2_*, *d_1_*, *e_1_*, *f_1_* setiform; a small subscutum situated posterior to the dorsal shield absent	10
10 (9)	Setae *f_1_* on dorsal shield; setae *lps*, *mps*, *c_1_*, *c_2_*, *d_1_*, *e_1_*, *f_1_* set on tubercles (Fig. 44); area between *pt* more heavily sclerotized, forming ridges; Taiwan	*Scutopalus osseus* (Tseng, 1980)
–	Setae *f_1_* on integument; setae *lps*, *mps*, *c_1_*, *c_2_*, *d_1_*, *e_1_*, *f_1_* set normally; area between *pt* normally sclerotized, not forming ridges; Ukraine	*Scutopalus trepidus* (Kuznetzov & Livshitz, 1979)
11 (8)	4 pairs of hysterosomal setae around genital shield; long slender platelet laterad genital shield present; with a narrow transverse sclertie behind main shield; Philippines	*Scutopalus philippinensis* (Corpuz-Raros, 1996)
–	3 pairs of hystersomal setae around genital shield; long slender platelet laterad genital shield absent; dorsal sclerites absent; Philippines	*Scutopalus makapalus* (Corpuz-Raros, 1996)
12 (7)	1 or more dorsal sclerites present (behind or laterad dorsal shield); dorsal shield rugose or reticulate (Fig. 42b, c); basifemora IV with 1 seta; pedipalpal tibiotarsus with 6 setae present and apophysis absent	13
–	Dorsal sclerites absent; dorsal shield sparsely granulate; basifemora IV with 2 setae; pedipalpal tibiotarsus with 5 setae and a rod-shaped dorsal apophysis present; Taiwan	*Scutopalus unguianalis* (Tseng, 1980)
13 (12)	Dorsal shield rugose (Fig. 42b); setae *f_1_* and *h_1_* on integument; dorsal setae (except *c_2_* and *h_2_*) distally rod-like (slightly clavate), with minute barbs; narrow transverse shield behind main dorsal shield present; Philippines	*Scutopalus rugosus* (Corpuz-Raros, 1996)
–	Dorsal shield reticulate (Fig. 42c); setae *f_1_* and *h_1_* on small platelets; dorsal setae (except *c_2_* and *h_2_*) broad and serrate; sclerites laterad and behind dorsal shield present; Brazil	*Scutopalus tomentosus* Rocha, Skvarla & Ferla, 2013

**Figures 40–44.**
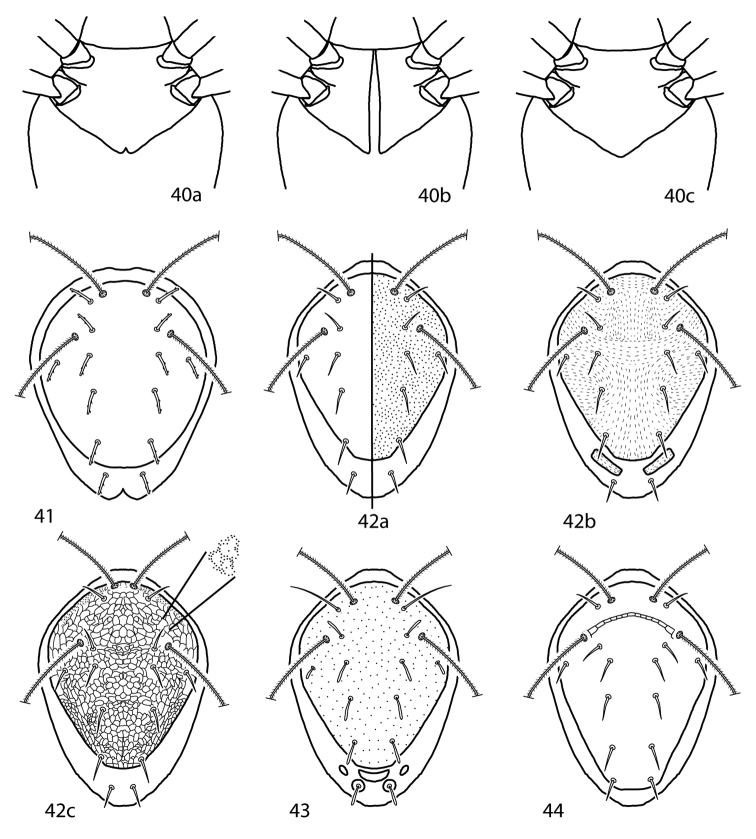
*Scutopalus* key illustrations. **40a** Coxae I–II faintly divided **40b** Coxae I–II totally divided **41** Coxae I–II fused medially **42** Dorsal shield with thick, rod-like setae present **43** Dorsal shield smooth or punctate **44a** Dorsal shield rugose **44b** Dorsal shield reticulate **44c** Dorsal shield sparsely granulate **45a** Setae *mps*, *c_1_*, *c_2_*, *d_1_*, *e_1_*, *f_1_* clavate **45b** Setae *mps*, *c_1_*, *c_2_*, *d_1_*, *e_1_*, *f_1_* setiform **46** Setae *lps*, *mps*, *c_1_*, *c_2_*, *d_1_*, *e_1_*, *f_1_* set on tubercles.

### Scirulinae Den Heyer, 1980

#### Scirula

Taxon classificationAnimaliaTrombidiformesCunaxidae

Berlese, 1887

##### Remarks.

This is a monobasic subfamily, with the single genus containing two described and one undescribed species. The subfamily and genus are therefore treated together.

##### Historical review.

Berlese (1887) erected *Scirula* for *Scirula impressa*. Thor and Willmann (1941) and Baker and Hoffmann (1948) redescribed and illustrated *Scirula impressa*. Den Heyer (1980c) erected Scirulinae for the then monotypic genus. Michocka (1987) reported *Scirula impressa* from Poland. Smiley (1992) redescribed and illustrated *Scirula impressa*. Lin (1997) described *Scirula papillata* from China.

##### Diagnosis.

*Gnathosoma*. **Pedipalps** 4-segmented and do not reach beyond the subcapitulum. A flange-like apophysis present on either the genua or tibiotarsi. Pedipalps end in a stout claw. **Subcapitulum** with 4 pairs of r setae (*hg_1-4_*).

*Idiosoma, dorsal*. Proterosoma covered in a plate which bears 4 pairs of setae: 2 pairs of simple setae (*lps* and *mps*) and 2 pairs of setose sensilla (*at* and *pt*). Dorsal hysterosoma may or may not be complemented with a plate. 6 dorsal setae, *c_1_–h_1_*, *c_2_* present. Cupule *im* present.

*Idiosoma, ventral*. **Coxae** I– IV fused, resulting in a complete shield covering the ventral idiosoma. Genital plates each bear 4 setae; 2 pairs of genital papillae visible underneath the plates. Cupule *ih* present. Anal plates bear 2 pairs of setae (*ps_1_* and *ps_2_*); 1 pair of setae born on integument next to anal plates.

##### Key to adult female *Scirula*

**Table:** 

1	Hysterosomal shield present (Fig. 45a); Japan, USA, Denmark, Italy	*Scirula impressa* Berlese, 1887
–	Hysterosomal shield absent (Fig. 45b); China, USA	*Scirula papillata* Lin, 1997

**Figures 45.**
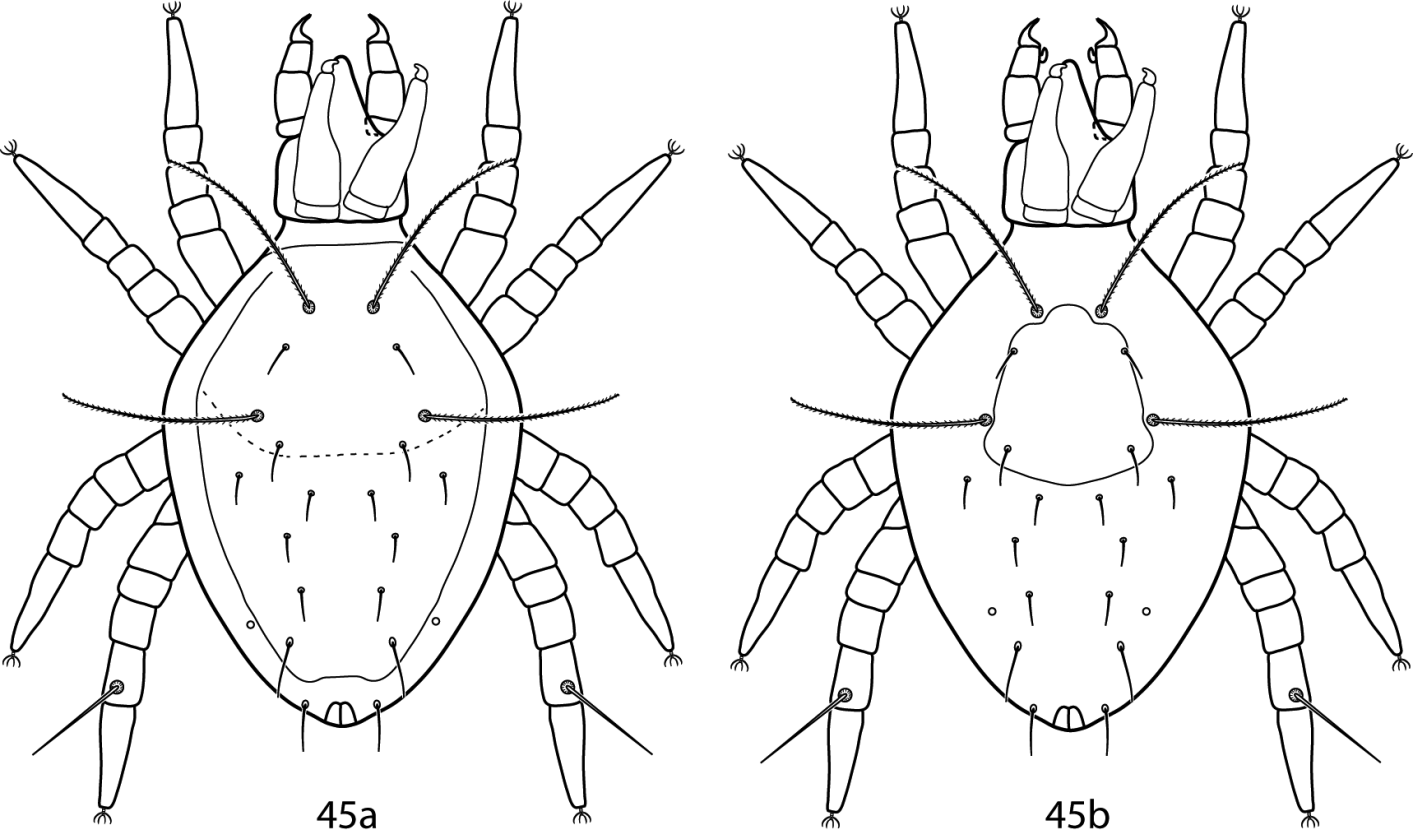
*Scirula* key illustrations. **45a** *Scirula impressa* **45b** *Scirula papillata*.

### Cunaxinae

Taxon classificationAnimaliaTrombidiformesCunaxidae

Den Heyer, 1978

#### Historical review.

Von Heyden (1826) erected *Cunaxa* for *Scirus setirostris*. Oudemans (1902) used Cunaxinae in the same sense that Trouessart (1892) used Scirinae, that is for those mites in the family Bdellidae (*sensu* Koch) that have pedipalps with a curved terminal segment and movable chela only (= Cunaxidae *sensu* Thor). Oudemans (1906) substituted Cunaxinae for Cunaxidae. Berlese (1916) erected *Dactyloscirus* as a subgenus of *Scirus* to accommodate *Scirus (Dactyloscirus) eupaloides*. Oudemans (1922) erected *Rosenhofia* to accommodate *Rosenhofia machairodus*. Vitzthum (1931) raised *Dactyloscirus* to full generic status but later (1940–43) treated it as a subgenus. Thor and Willmann (1941) again elevated *Dactyloscirus* to generic status and designated *Dactyloscirus eupaloides* as the type specimen. Baker and Hoffmann (1948) regarded *Dactyloscirus* as a senior synonym of *Cunaxa*. Smiley (1975) synonymized *Rosenhofia* with *Dactyloscirus*. Den Heyer (1978a) preserved the name Cunaxinae, but limited its concept to those cunaxids possessing 5-segmented pedipalps that extend past the subcapitulum by at least the distal two segments; he also erected *Armascirus*. Den Heyer (1979d) erected *Rubroscirus* for *Rubroscirus africanus*. Gupta and Ghosh (1980) erected *Indocunaxa*. Smiley (1992) synonymized *Rubroscirus* with *Cunaxa* but failed to give his reasoning for doing so. Den Heyer (2006) erected *Riscus* for a species known only from Thailand. Castro and Den Heyer (2008) erected *Cunaxatricha* and provided a key to the genera of Cunaxinae. Den Heyer and Castro (2008) erected *Allocunaxa* for a Neotropical species, synonymized *Indocunaxa* with *Armascirus*, and provided the most up-to-date key to world genera of Cunaxinae.

#### Diagnosis.

*Gnathosoma*. **Pedipalps** 5-segmented and extend beyond the subcapitulum by at least the distal half of the tibiae. Basifemora and telofemora fused but often dark line remains to indicate the division between the segments; telofemora and genua also fused in this manner in *Allocunaxa*. Apophyses may be present on the telofemora and between the genua and tibiotarsi. Tibiotarsi end in a strong claw. **Chelicera** with or without seta. **Subcapitulum** with up to 6 pairs of setae; setae *hg_1_*_–_*_4_* always present, 2 pairs of adoral setae present or absent. Setae *hg_4_* longest. In species with pedipalpal apophyses, the apophyses of the males shorter.

*Idiosoma, dorsal*. Female proterosoma bears a shield complemented with 2 pairs of setae (*lps* and *mps*) and 2 pairs of setose sensillae (*at* and *pt*). Dorsal hysterosoma may bear any combination of a median plate and lateral platelets (i.e., median plate and platelets absent, only median plate present, only lateral platelets present, or both median plate and lateral platelets present). Median plate, if present, may be complemented with 0–6 pairs of dorsal setae; lateral platelets, if present, may bear setae *c_2_*. Setae not born on plates or platelets may be born on tiny platelets barely larger than the setal socket. Integument that does not bear plates or platelets striated. Males differ in that the dorsal shields often more extensive and may be holodorsal.

*Idiosoma, ventral*. **Coxae** I–II fused or divided and may coalesce medially to form a sternal shield; coxae III–IV fused or divided and may extend caudally past the genital plates. Coxae each complemented 0–3 setae. Genital plates each bear 4 setae (*g_1_*_–_*_4_*); 2 pairs of genital papillae visible underneath the plates. Anal plates complemented with at least one pair of setae, *ps_1_*. Setae *ps_2_* present or absent, either on the anal plates or on the integument adjacent to the anal plates. Setae *h_2_* present ventrally on the integument adjacent to the anal plates. Cupule *ih* present laterad of *h_2_*. Integument that does not bear plates striated. **Legs.** Tarsi constricted apically so as to end in lobes. A trichobothrium on tibia IV present or absent.

#### Key to adult female Cunaxinae

(modified from Den Heyer and Castro 2008a)

**Table:** 

1	Anal seta *ps_2_* absent; pedipalp telofemora with dorsal simple seta (Figs 46a–e); tarsal lobes small to medium size (Fig. 47a); dorsal plates reticulated or not (Figs 48a–c) Cunaxini	2
–	Anal seta *ps_2_* present; pedipalp telofemora with dorsal spine-like seta (Figs 46f, g); tarsal lobes medium to large size (Fig. 47b); dorsal plates always reticulated (Fig. 48c) Armascirini	6
2 (1)	Dorsal plates never reticulated (Figs 48a, b); integumental striae smooth or lobed; coxae II–IV setal formula usually 1-3-2 (rarely 2-3-1)	*Cunaxa* Von Heyden, 1826
–	Dorsal plates usually reticulated (Fig. 48c); integumental striae usually papillated; coxae II–IV setal formula usually 1-3-1	3
3 (2)	Pedipalpal telofemora with one or more apophyses (Fig. 46a); sensillae *at* and *pt* not densely pilose	*Rubroscirus* Den Heyer, 1979
–	Pedipalpal telofemora without apophyses (Figs 46b–e); sensillae *at* and *pt* densely pilose	4
4 (3)	Tibiae IV trichobothrium present	5
–	Tibiae IV trichobothrium absent	*Cunaxatricha* Castro & Den Heyer, 2008
5	Articulation joint between pedipalpal telofemora and genua functional (Fig. 46b)	*Riscus* Den Heyer, 2006
–	Articulation joint between pedipalpal telofemora and genua fused/non-functional (Fig. 46c)	*Allocunaxa* Den Heyer & Castro, 2008
6 (1)	Pedipalpal basifemora with simple seta (Fig. 46f); coxae II–IV setal formula usually 1-3-3 (male) or 2-3-3 (female); famulus normal; pedipalpal apophyses (when present) usually long in females and short in males, and with pointed apices (Fig. 46f)	*Armascirus* Den Heyer, 1978
–	Pedipalpal basifemora with spine-like seta (Fig. 46g); coxae II–IV setal formula usually 3-3-3; famulus large, broad based with tri-pronged tip; pedipalpal apophyses (when present) usually equal length in females and males, and with bulbous apices (Fig. 46g)	*Dactyloscirus* Berlese, 1916

**Figures 46–48.**
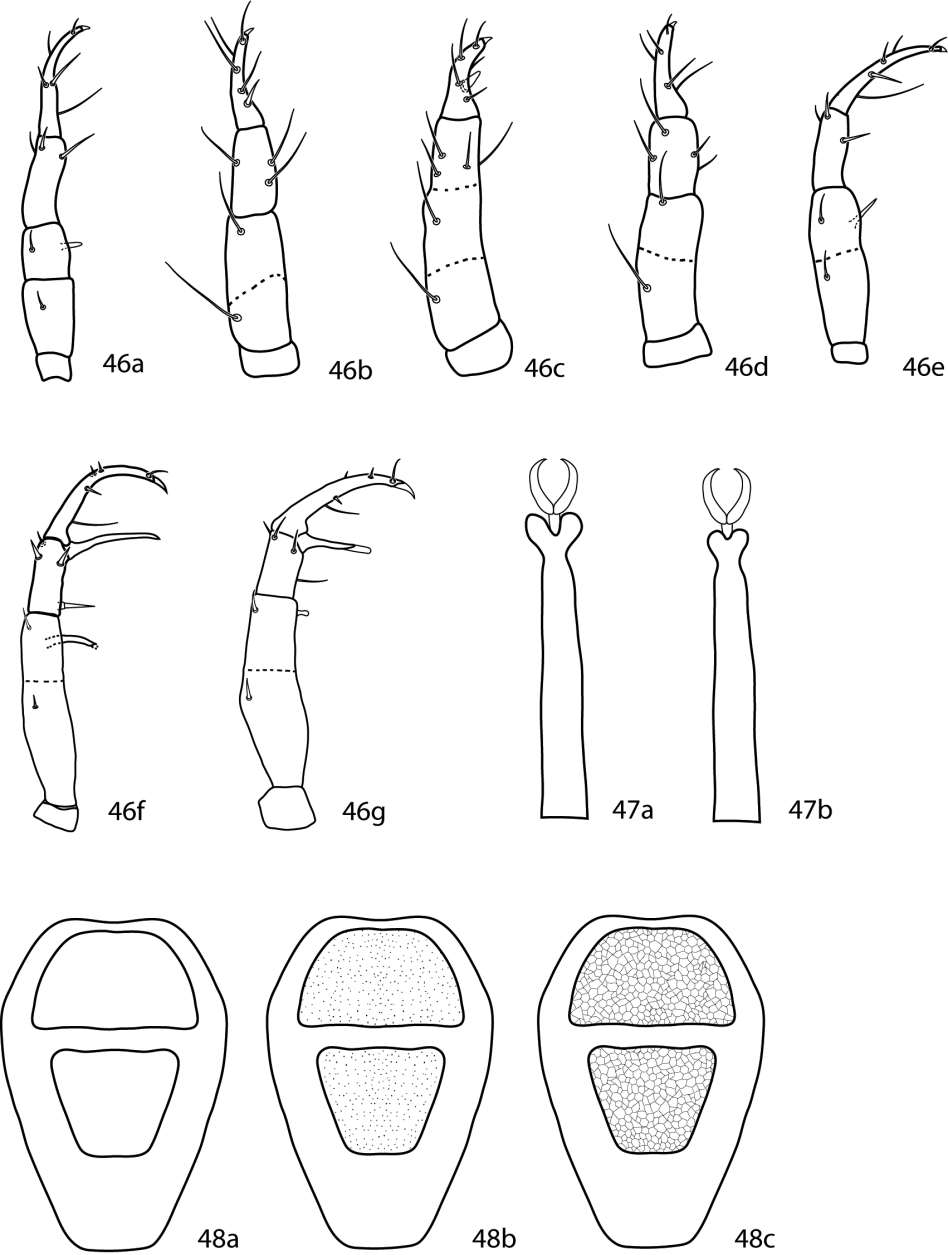
Cunaxinae key illustrations. **46** Pedipalps, dorsal **46a** *Rubroscirus* **46b** *Riscus* **46c** *Allocunaxa* **46d** *Cunaxatricha* **46e** *Cunaxa* **46f** *Armascirus* **46g** *Dactyloscirus*. 47a, b. Distal end of tarsus **47a** *Armascirini*, showing large tarsal lobes **47b** *Cunaxini*, showing small to medium tarsal lobes **48a–c** Idiosoma, dorsal. Setae and cupules have been removed for clairity. Shape of proterosomal plate and presence or absence, shape, and extent of hysterosomal plate(s) will differ between species **48a** Plates smooth **48b** Plates with dot-like pattern **48c** Plates with reticulated pattern.

### Allocunaxa

Taxon classificationAnimaliaTrombidiformesCunaxidae

Den Heyer & Castro, 2008

#### Historical review.

Den Heyer and Castro (2008a) erected *Allocunaxa* for *Allocunaxa heveae*.

#### Diagnosis.

*Gnathosoma*. **Pedipalps** 5-segmented, end in a strong claw, and extend beyond the subcapitulum by at least the last segment. Pedipalpal apophyses absent. Basifemora complemented with a long simple seta and telofemora with a short simple seta; these two segments fused, although a line remains visible and they can thus be differentiated. Telofemora and genu nearly fused, although a line remains visible and they can thus be differentiated. **Subcapitulum** complemented with 6 pairs of setae (*hg_1–4_* and 2 pairs of adoral setae) and covered by integumental papillae.

*Idiosoma, dorsal*. Proterosoma with an ill-defined, weakly sclerotized shield that bears 2 pairs of setose sensillae (*at* and *pt*) and 2 pairs of simple setae (*lps* and *mps*). 7 pairs of setae, *c_1–2_*, *d_1_–h_1_*, present. Cupule *im* present, usually posteriolaterad of *e_1_*. Integument striated.

*Idiosoma, ventral*. **Coxae** I and II fused. Coxae III and IV fused. Genital plates each bear 4 setae; 2 pairs of genital papillae visible underneath the plates. Integument between plates striated and bears 4 pairs of additional setae. **Legs** shorter than the body. Leg 4 longest. Famulus on tarsi I normally shaped. Tarsi constricted apically, resulting in large tarsal lobes. Trichobothrium on leg tibia IV present. Ambulacral claws on either side of a 4-rayed empodium present.

### Armascirus

Taxon classificationAnimaliaTrombidiformesCunaxidae

Den Heyer, 1978

#### Historical review.

The first *Armascirus* was described by Kramer (1881) as *Scirus taurus*. Berlese (1888) described *Scirus taurus* var. *bison*. Banks (1894) described *Scirus quadripilis*. Thor (1902) transferred *Scirus taurus* to *Cunaxa*. Banks (1914) described *Cunaxa armata*. Miller (1925) reported *Scirus quadripilis* from Ohio. Womersley (1933) reported *Cunaxa taurus* from Australia. Thor and Willmann (1941) transferred *Scirus taurus* var. *bison* to *Cunaxa* and raised it to full species status, viz. *Cunaxa bison* and transferred *Scirus quadripilis* to *Cunaxa*; they also redescribed and figured *Cunaxa armata*, *Cunaxa bison*, *Cunaxa quadripilis*, and *Cunaxa taurus*. Baker and Hoffmann (1948) synonymized *Scirus quadripilis* and *Cunaxa armata* with *Cunaxa taurus*; they followed Thor and Willmann (1941) in placing *Cunaxa taurus* var. *bison* in *Cunaxa* but declined to recognize it as a species and instead kept it as a variety or subspecies of *Cunaxa taurus*. Zaher et al. (1975b) collected *Cunaxa taurus* in Egypt. Chaudhri (1977) described *Dactyloscirus ebrius* and *Dactyloscirus fuscus* from Pakistan. Den Heyer (1978a) split *Armascirus* from *Dactyloscirus* and *Cunaxa* and raised the subfamily Cunaxinae to accommodate them, thus refining the definitions of all three genera; he transferred *Cunaxa taurus* and *Cunaxa bison* to the new genus *Armascirus*; and described *Armascirus huyssteeni*, *Armascirus lebowensis*, *Armascirus limpopoensis*, and *Armascirus albiziae*. Kuznetzov and Livshitz (1979) redescribed and figured *Cunaxa taurus* and *Cunaxa bison* from Russia, either disagreeing with or being unaware of Den Heyer’s 1978 publication. Tseng (1980) reported *Armascirus taurus* from Taiwan. Chaudhri (1980) described *Dactyloscirus fixus* from Pakistan. Den Heyer (1980c) erected the tribe Armascirini and made *Dactyloscirus* and *Armascirus* the sole representatives. Gupta and Ghosh (1980) erected *Indocunaxa*, a monotypic genus with *Indocunaxa smileyi* as the type species. Liang (1983) reported *Armascirus taurus* from China. Shiba (1986) described *Armascirus hastus* and *Armascirus multioculus*. Michocka (1987) described *Dactyloscirus rafalskii* from Poland. *Armascirus mactator* and *Armascirus pluri* were described by Muhammad and Chaudhri (1991b). Smiley (1992) described *Armascirus gimplei*, *Armascirus anastosi*, *Armascirus harrisoni*, *Armascirus heryfordi*, *Armascirus virginiensis*, *Dactyloscirus bakeri*, and *Dactyloscirus campbelli*; he also transferred *Armascirus bison* to *Dactyloscirus* (which was later returned to *Armascirus* by Den Heyer and Castro 2008a). Corpuz-Raros (1995) described *Armascirus garciai* and *Armascirus makilingensis* from the Philippines. Hu (1997) reported *Armascirus bison* and *Armascirus taurus* from China. Bashir and Afzal (2005) described *Armascirus satianaensis* and *Armascirus asghari*. Corpuz-Raros and Gruèzo (2007) described *Armascirus javanus*. Corpuz-Raros (2008) described *Dactyloscirus bifidus*. Bashir, Afzal, and Khan (2008) described four species from Pakistan: *Armascirus akhtari*, *Armascirus jasmina*, *Armascirus sabrii*, and *Armascirus gojraensis*. Den Heyer and Castro (2008a) synonymized *Indocunaxa* with *Armascirus* and transferred *Dactyloscirus bison*, *Dactyloscirus campbelli*, *Dactyloscirus ebrius*, *Dactyloscirus fixus*, *Dactyloscirus fuscus*, and *Dactyloscirus rafalskii* to *Armascirus javanus*. Corpuz-Raros (2008) described *Armascirus apoensis*. Kalúz (2009) described *Armascirus cyaneus* and *Armascirus cerris* from Central Europe Skvarla and Dowling (2012) described *Armascirus ozarkensis*, *Armascirus pennsylvanicus*, and *Armascirus primigenius*. Den Heyer and Castro (2012) described *Armascirus brasiliensis* and *Armascirus bahiaensis*. Kalúz and Vrabec (2013) described *Armascirus fendai* and *Armascirus masani*.

#### Diagnosis.

*Gnathosoma*. **Pedipalps** 5-segmented, end in a strong claw, and extend beyond the subcapitulum by at least the last segment. Apophysis between the genua and tibiotarsi, which tapers to a point, usually present; this apophysis shorter in males than in females. Basifemora complemented with a simple seta; telofemora with a spine-like seta. These two segments fused, although a line remains visible and they can thus be differentiated. **Subcapitulum** complemented with 6 pairs of setae (*hg_1–4_* and 2 pairs of adoral setae). It can be covered by integumental papillae which are either randomly distributed or form a polygonal, reticulated pattern.

*Idiosoma, dorsal*. Female dorsal idiosoma with at least one sclerotized plate that bears 2 pairs of setose sensillae (*at* and *pt*) and 2 pairs of simple setae (*lps* and *mps*). 0–4 other major plates and platelets may also be present. All plates, if present, covered by integumental papillae that form a reticulated pattern. Integument between the plates is striated. 7 pairs of setae, *c_1–2_*, *d_1_–h_1_*, present. Each seta, when not on a major plate or platelet, surrounded by a minute platelet that is only slightly larger than the setal socket. Cupule *im* present, usually laterad or in the proximity of *e_1_*. Dorsal idiosoma of males is similar except a single large plate complemented with *c_1–2_*, *d_1_–e_1_* present.

*Idiosoma, ventral*. **Coxae** reticulated in the same manner as the dorsal plates. Coxae I–II often fused; Coxae III–IV often fused. Setal formula of coxae I–IV in males 3-1-3-3 (including the paracoxal seta), in females 3-2-3-3 (including the paracoxal seta). Genital plates each bear 4 setae; 2 pairs of genital papillae visible underneath the plates. Anal plates bear 1 pair of setae (*ps_1_*). 2 pairs of setae (*ps_2_* and *h_2_*) associated with but do not occur on the anal plates. Cupule *ih* present in close proximity to *h_2_*. Integument between plates striated and bears 5–7 pairs of additional setae. The ventral idiosoma of males similar except the coxae are much more extensive. A sclerotized aedeagus is often visible in association with the genital plates. **Legs** comparatively long, at least ¾ the length, and often longer than the body. Famulus on tarsi I normally shaped. Tarsi are constricted apically, resulting in large tarsal lobes. Trichobothrium on leg tibia IV present. Ambulacral claws occur on either side of a 4-rayed empodium.

#### Key to adult female *Armascirus*

(modified from Kalúz and Vrabec 2013)

*Dactyloscirus bifidus* Corpuz-Raros, 2008 is transferred to *Armascirus* as it posessess a spine-like seta on the pedipalpal basifemora.

*Armascirus gojraensis* and *Armascirus sabrii* appear to be nymphs based on the leg setal counts given in the original descriptions. Having not seen the type material, however, they are retained within the key. Caution should be exercised if these species are reached.

**Table:** 

1	Hysterosomal median shield present (Figs 49a–h, 50a–d)	2
–	Hysterosomal median shield absent (Figs 51a–c)	30
2 (1)	Median shield complemented with setae, small or large (Figs 49a–h)	3
–	Median shield not complemented with setae, small (Figs 50a–d)	22
3 (2)	One pair of setae (*d_1_*) on hysterosomal median shield (Figs 49a–f)	4
–	Two or more pairs of setae on hysterosomal median shield (Figs 49g–h)	18
4 (3)	Lateral hysterosomal platelets present (Figs 49a–d)	5
–	Lateral hysterosomal platelets absent (Figs 49e, f)	15
5 (4)	Setae *c_1_* very short, the distance between the bases of *c_1_–c_1_* 20 times the length of *c_1_*; venter caudally from coxae II with 5 pairs of simple setae (excluding genital, coxal, and anal setae); Poland	*Armascirus rafalskii* (Michocka, 1987)
–	Setae *c_1_* longer, the distance between the bases of *c_1_–c_1_* less than 10 times the length of *c_1_*; venter caudally from coxae II with 6 or more pairs of simple setae (excluding genital, coxal, and anal setae)	6
6 (5)	The distance between caudal parts of hysterosomal lateral platelets wider than the distance between their frontal parts (Figs 49a, b)	7
–	The distance between caudal parts of hysterosomal lateral platelets shorter than the distance between their frontal parts (Figs 49c, d)	9
7 (6)	Lateral hysterosomal platelets equal to or longer than hysterosomal median shield (Fig. 49a); venter caudally from coxae II with 6 pairs of simple setae (excluding genital, coxal, and anal setae); Pakistan	*Armascirus jasmina* Bashir, Afzal & Khan, 2008
–	Lateral hysterosomal platelets shorter than hysterosomal median shield (Fig. 49b); venter caudally from coxae II with 7 pairs of simple setae (excluding genital, coxal, and anal setae)	8
8 (7)	Pedipalpal genua with 3 spls, 1 sts; important leg I–IV sts chaetotaxy: coxae 3-1-3-2, basifemora 4-5-3-1, genua 8-8-6-5, tibiae 5-6-6-6, tarsi 15-12-8-9; Pakistan	*Armascirus akhtari* Bashir, Afzal & Khan, 2008
–	Pedipalpal genua with 3 spls; important leg I–IV sts chaetotaxy: coxae 3-2-3-3, basifemora 4-4-3-3, genua 8-4-6-7, tibiae 6-5-6-5, tarsi 11-10-9-7; Pakistan	*Armascirus satianaensis* Bashir & Afzal, 2005
9 (6)	Venter caudally from coxae II with 4 pairs of simple setae (excluding genital, coxal, and anal setae); Brazil	*Armascirus bahiaensis* Den Heyer & Castro, 2012
–	Venter caudally from coxae II with 6 pairs of simple setae (excluding genital, coxal, and anal setae)	10
–	Venter caudally from coxae II with 7 pairs of simple setae (excluding genital, coxal, and anal setae)	14
–	Venter caudally from coxae II with 8 pairs of simple setae (excluding genital, coxal, and anal setae); South Africa	*Armascirus albiziae* Den Heyer, 1978
10 (9)	Tarsus I with more than 27 setae; tarsus II with at least 24 setae	11
–	Tarsus I with less than 25 setae; tarsus II with less than 23 setae	12
11 (10)	Leg genua I with 4 bsl, 4 sts; genital valve with random dot-like lobes; tarsal sts chaetotaxy I–IV 29-25-23-22; Pakistan	*Armascirus pluri* Muhammad & Chaudhri, 1991
–	Leg genua I with 2 asl, 4 bsl, 3 sts; genital valve longitudinal rows of dot-like lobes; tarsal sts chaetotaxy I–IV 29-24-22-21; Pakistan	*Armascirus mactator* Muhammad & Chaudhri, 1991
12 (10)	Pedipalpal telofemora with 1 apophysis, 2 spls; pedipalpal genua with 1 ap, 2 spls, 2 sts; South Africa	*Armascirus huyssteeni* Den Heyer, 1978
–	Pedipalpal telofemora with 1 apophysis, 1 spls; pedipalpal genua with 1 ap, 3 spls, 1 sts	13
13(12)	Genua II with 1 asl, 5 sts; genua IV with 2 asl, 5 sts; cosmopolitan	*Armascirus taurus* (Kramer, 1881)
–	Genua II with 1 asl, 6 sts; genua IV with 1 asl, 4 or 5 sts; USA	*Armascirus primigenius* Skvarla & Dowling, 2012
14 (9)	Median shield pointed caudally (Fig. 49c); Pakistan	*Armascirus asghari* Bashir & Afzal, 2005
–	Median shield truncated caudally (Fig. 49d); Brazil	*Armascirus brasiliensis* Den Heyer & Castro, 2012
15 (4)	Hysterosomal median shield with a straight or concave frontal margin and with very acute anterior lateral corners (angle less than 45°) (Fig. 49e)	16
–	Hysterosomal median shield with convex frontal margin and with rounded anterior lateral corners (Fig. 49f)	17
16 (15)	Pedipalpal genua with 1 ap, 2 spls, 1 sts; legs I–IV sts formulae (excluding solenidia): basifemora 1-2-1-0; telofemora 4-4-4-4; genua 6-7-5-6; *h_1_* 4 times the length of *c_1_*;hysterosomal shield width: length = 2.2:1; Pakistan	*Armascirus sabrii* Bashir, Afzal & Khan, 2008
–	Pedipalpal genua with 1 ap, 3 spls, 1 sts; legs I–IV sts formulae (excluding solenidia): basifemora 2-2-1-1; telofemora 4-4-4-3; genua 8-6-6-6; *h_1_* 3 times the length of *c_1_*;hysterosomal shield width: length 1.5:1; Pakistan	*Armascirus gojraensis* Bashir, Afzal & Khan, 2008
17 (15)	Apophysis adjoining genu and tibiotarsus shorter than pedipalpal tibiotarsus; pedipalpal telofemoral apophyses three times longer than spine-like seta; distance between the bases of *sci–sci* 9 times the length of *sci*; Brazil, Mexico	*Armascirus bison* (Berlese, 1988)
–	Apophysis adjoining genu and tibiotarsus longer than pedipalpal tibiotarsus; pedipalpal telofemoral apophyses three times longer than spine-like seta; distance between the bases of *sci–sci* 5 times the length of *sci*; Pakistan	*Armascirus fixus* (Chaudhri, 1980)
18 (3)	Hysterosomal median shield with 2 pairs of setae (*c_1_*, *d_1_*) (Fig. 49g)	19
–	Hysterosomal median shield with more than 3 pairs of setae (Fig. 49h)	20
19 (18)	Pedipalpal telofemora with 2 ap, 1 spls; pedipalpal genua with 2 spls, 2 sts; venter caudally from coxae II with 6 pairs of simple setae (excluding genital, coxal, and anal setae); tarsi I–IV with 21-20-15-13 sts (excluding solenidia); the distance between bases of *c_1_–c_1_* 4 times the distance of *h_1_–h_1_*; distance between *c_1_–c_1_* 5 times the length of *c_1_*	*Armascirus anastosi* Smiley, 1992
–	Pedipalpal telofemora with 1 ap, 1 spls; pedipalpal genua with 3 spls, 1 sts; venter caudally from coxae II with 5 pairs of simple setae (excluding genital, coxal, and anal setae); tarsi I–IV with 19-13-13-13 sts (excluding solenidia); the distance between *c_1_–c_1_* 2 times the distance between *h_1_–h_1_*; the distance between *c_1_–c_1_* 4 times the length of *c_1_*	*Armascirus heryfordi* Smiley, 1992
20 (18)	Apophysis adjacent to pedipalpal genua and tibiotarsi present	*Armascirus multioculus* Shiba, 1986
–	Apophysis adjacent to pedipalpal genua and tibiotarsi absent	21
21 (20)	5 pairs of genital setae; pedipalp claw bifid (Fig. 52a); hysterosomal setae not serrate; Philippines	*Armascirus apoensis* Corpuz-Raros, 2008
–	4 pairs of genital setae; pedipalp claw entire, not bifid (Fig. 52b); hysterosomal setae serrate; Pakistan	*Armascirus fuscus* (Chaudhri, 1977)
22 (2)	Lateral hysterosomal platelets present (Figs 50a–c)	23
–	Lateral hysterosomal platelets absent (Fig. 50d)	27
23 (22)	Hysterosomal median shield width: length 1:1; venter caudally from coxae II with 6 or 7 pairs of sts (excluding genital and anal setae)	24
–	Hysterosomal median shield width: length 2:1; venter caudally from coxae II with 5 or 6 pairs of sts (excluding genital and anal setae)	25
24 (23)	Hysterosomal platelets large, as long as median shield (Fig. 50a); venter caudally from coxae II with 7 sts; pedipalp telofemur with 1 apophysis	*Armascirus cerris* Kalúz, 2009
–	Hysterosomal platelets about 1/3 the length of median shield; venter caudally from coxae II with 6 sts; pedipalp telofemur with 2 apophysis	*Armascirus fendai* Kalúz & Vrabec, 2013
25 (23)	Hysterosomal platelets as long as median shield (Fig. 50b)	26
–	Hysterosomal platelets ½ as long as median shield (Fig. 50c); Mexico, USA	*Armascirus gimplei*
26 (25)	Hysterosomal plate concave on lateral edges (Fig. 53a); USA	*Armascirus ozarkensis* Skvarla & Dowling, 2012
–	Hysterosomal plate not concave on lateral edges (Fig. 53b); Japan	*Armascirus hastus* Shiba, 1986
27 (22)	Apophysis on pedipalp telofemur extends to distal margin of segment; 2 pairs of ventral pregenital setae thickened and spiculate; *f_1_* 1/3 length of *h_1_*; Philippines	*Armascirus makilingensis* Corpuz-Raros, 1995
–	Apophysis on pedipalp telofemur extends well beyond distal margin of segment; ventral pregenital setae not thickened and spiculate; *f_1_* subequal to *h_1_*	28
28 (27)	Pedipalpal telofemora with 2 ap, 1 spls; the distance between the bases of *c_1_–c_1_* two times the distance of *d_1_–d_1_*; South Africa	*Armascirus limpopoensis* Den Heyer, 1978
–	Pedipalp telofemora with 1 ap, 1 spls; the distances between the bases of *c_1_–c_1_* = *d_1_–d_1_*	29
29 (28)	Pedipalp tibiotarsus with 1 spls, 4 sts; USA	*Armascirus harrisoni* Smiley, 1992
–	Pedipalp tibiotarsus with 1 spls, 3 sts; Canada	*Armascirus bakeri* (Smiley, 1992)
30 (1)	Pedipalpal telofemoral apophyses long, reaching apical apophysis on pedipalpal genu; lateral platelets present	31
–	Pedipalpal telofemoral apophyses short, not reaching apical apophysis on pedipalpal genu; lateral platelets present or absent	32
31 (30)	Pedipalpal basifemora with 1 subrectangular apophysis; pedipalp tibiotarsal spls 3 times the length of terminal claw; hysterosomal platelets small, equal in length to *c_2_* (Fig. 51a); coxal chaetotaxy I–IV 3-2-3-3; South Africa	*Armascirus lebowensis* Den Heyer, 1978
–	Pedipalpal basifemora without subrectangular apophysis; pedipalp tibiotarsal spls equal in length to terminal claw; hysterosomal platelets long, 2–3 times the length of *c_2_* (Fig. 51b); coxal chaetotaxy I–V 3-1-3-1; USA	*Armascirus campbelli* (Smiley, 1992)
32 (30)	Coxal setal count I–IV 3-2-3-3	33
–	Coxal setal count I–IV 3-2-3-2	35
–	Coxal setal count I–IV 3-3-3-3	*Armascirus bifidus* (Corpuz-Raros, 2008)
33 (32)	Pedipalpal telofemora with 1 apophysis, 2 spls, 1 sts; the distance between *d_1_–d_1_* 9 times the length of *d_1_*; pedipalpal genua with 2 spls, 1 sts; Slovakia	*Armascirus cyaneus* Kalúz, 2009
–	Pedipalpal telofemora with 1 apophysis, 2 spls; the distance between *d_1_–d_1_* 4 times the length of *d_1_*; pedipalpal genua chaetotaxy not as above	34
34 (33)	Hysterosomal platelets present (Fig. 51b); pedipalpal genua with 2 spls, 2 sts; basifemora with 5-5-4-2 sts; USA	*Armascirus virginiensis* Smiley, 1992
–	Hysterosomal platelets absent (Fig. 51c); pedipalpal genua with 1 spls, 1 sts; basifemora with 6-6-4-2 sts; Philippines	*Armascirus javanus* Corpuz-Raros & Gruèzo, 2007
35 (32)	Pedipalpal telofemoral apophyses as long as width of telofemora; pedipalpal genu with 1 apophysis, 2 spls, 2 sts; USA	*Armascirus pennsylvanicus* Skvarla & Dowling, 2012
–	Pedipalpal telofemoral apophyses only 1/3 width of telofemora; pedipalpal genu with 1 apophysis, 3 spls, 1 sts; Philippines	*Armascirus garciai* Corpuz-Raros, 1995

**Figures 49–53.**
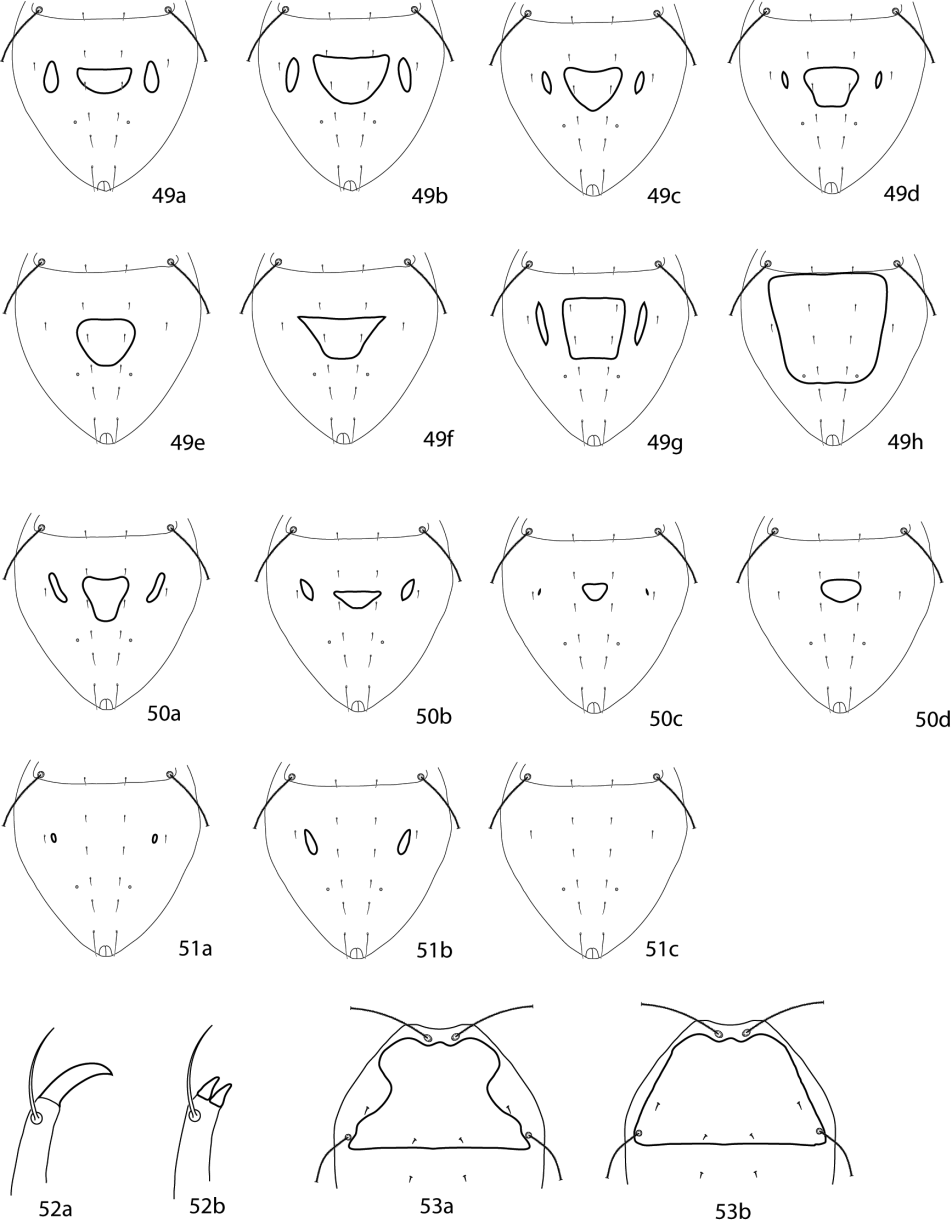
*Armascirus* key illustrations. **49–51** Dorsal idiosoma **49a–e** Hysterosomal shield complemented with setae **50a–d** Hysterosomal shield small, not complemented with setae **51a–c** Hysterosomal shield absent **52a, b** Pedipalp tibiotarsal claw **52a** Single claw **52b** Bifid claw **53a** Hysterosomal plate concave on lateral edges **53b** Hysterosomal plate not concave on lateral edges.

#### Key to adult male *Armascirus*

(modified from Kalúz and Vrabec 2013)

**Table:** 

1	Venter with 5 or fewer pairs of setae, excluding genital, anal, and adanal setae; setal formula of coxae I–IV not as below; setal formula of basifemora I–IV not as below	2
–	Venter with 6 pairs of setae, excluding genital, anal, and adanal setae; setal formula of coxae I–IV 3-2-3-3; setal formula of basifemora I–IV 5-5-4-2; cosmopolitan	*Armascirus taurus* (Kramer, 1881)
2 (1)	Setal formula of basifemora I–IV 5-5-4-1; Pakistan	*Armascirus ebrius* (Chaudhri, 1977)
–	Setal formula of basifemora I–IV not as above	3
3 (2)	Coxae I–IV setal formula 3-1-3-3; papillae on circular region anterior to setae *pt* present; South Africa	*Armascirus huyssteeni* Den Heyer, 1978
–	Coxae I–IV setal formula 3-2-3-3; papillae on circular region anterior to setae *pt* present or absent	4
4 (3)	Setal formula of basifemora I–IV 5-4-3-0; papillae on circular region anterior to setae *pt* present; South Africa	*Armascirus limpopoensis* Den Heyer, 1978
–	Setal formula of basifemora I–IV not as above; papillae on circular region anterior to setae *pt* absent; South africa	5
5 (4)	Genua I with 3 asl, 5 sts; South Africa	*Armascirus lebowensis* Den Heyer, 1978
–	Genua I with 2 asl, 1 mst, 5 sts; Ukraine	*Armascirus masani* Kalúz & Vrabec, 2013

### Cunaxa

Taxon classificationAnimaliaTrombidiformesCunaxidae

Von Heyden, 1826

#### Historical review.

Hermann (1804) erected *Scirus* for *Scirus setirostris* and placed it with two mites that are now considered to belong to the family Bdellidae. Von Heyden (1826) erected *Cunaxa* for *Scirus setirostris*. Dugés (1834a) described *Scirus elaphus*. Dugés (1834b) described *Scirus tenuirostris*. Koch (1836) described *Scirus stabulicola* and *Scirus sagax* and later (1838) *Scirus paludicola*. Gervais (1841) described *Scirus obisium*. Berlese (1887) described *Scirus capreolus*. Berlese (1888) synonymized *Scirus elaphus*, *Scirus stabulicola*, *Scirus sagax*, and *Scirus paludicola* with *Scirus setirostris*. Thor (1902) erected Cunaxidae and split *Cunaxa* from Bdellidae. Ewing (1913) described *Scirus laricis*. *Scirus setirostris* var. *gazella* was described by Berlese (1916). Thor and Willmann (1941) redescribed and figured *Scirus laricis* after transferring it to *Cunaxa*; they also transferred *Scirus setirostris* var. *gazella* to *Cunaxa*, though kept it as a subspecies of *Cunaxa setirostris* and synonymized *Scirus tenuirostris* and *Scirus obisium* with *Cunaxa setirostris*. Baker and Hoffmann (1948) redescribed and figured *Cunaxa setirostris* var. *gazella* and *Cunaxa capreolus* and described *Cunaxa womersleyi* and *Cunaxa veracruzana*. Zaher et al. (1975b) reported *Cunaxa setirostris* and *Cunaxa capreolus* from Egypt. Den Heyer (1978a) erected Cunaxinae and assigned *Cunaxa* to the subfamily. Den Heyer (1979e) elevated *Cunaxa setirostris* var. *gazella* to full species status, viz. *Cunaxa gazella*; described *Cunaxa carina*, *Cunaxa terrula*, *Cunaxa lamberti*, *Cunaxa meiringi*, and *Cunaxa grobleri* and redescribed and figured *Cunaxa capreola* and *Cunaxa gazella*. He then (Den Heyer 1979f) described five more species from South Africa: *Cunaxa hermanni*, *Cunaxa sordwanaensis*, *Cunaxa potchensis*, *Cunaxa brevicrura*, and *Cunaxa magoebaensis*. Kuznetzov and Livshitz (1979) redescribed and figured *Cunaxa capreolus* and *Cunaxa setirostris* from Russia. Chaudhri (1980) described *Cunaxa doxa*. Tseng (1980) reported *Cunaxa womersleyi* and *Cunaxa setirostris* from Taiwan. Gupta and Ghosh (1980) described *Cunaxa myabunderensis*. Gupta and Paul (1985) described *Cunaxa prinia*. Bu and Li (1987c) reported *Cunaxa capreola* from China. Michocka (1987) reported *Cunaxa setirostris* from Poland. Muhammad et al. described *Rubroscirus valentis* from Pakistan. Smiley (1992) described *Cunaxa mageei*, *Cunaxa thailandicus*, *Cunaxa evansi*, and *Cunaxa neogazella*; he also synonymized *Rubroscirus* with *Cunaxa*, though failed to include his evidence for doing so. Gupta (1992) described *Cunaxa anacardae* and *Cunaxa magniferae*. Muhammad and Chaudhri (1993) described *Rubroscirus rasile* and *Rubroscirus otiosus* from Pakistan. Corpuz-Raros and Garcia (1995) described five species from the Philippines: *Cunaxa luzonica*, *Cunaxa romblonensis*, *Cunaxa pantabanganensis*, *Cunaxa cogonae*, and *Cunaxa mercedesae*. Hu (1997) reported 28 species of Cunaxidae from China. Khaustov and Kuznetzov (1998) described *Cunaxa heterostriata*, *Cunaxa anomala*, *Cunaxa sudakensis* and *Cunaxa bochkovi*. Chinniah and Mohanasundaram (2001) described *Cunaxa eupatoriae*. Sergeyenko (2003) described *Cunaxa dentata*. Sionti and Papadoulis (2003) described *Cunaxa thessalica* from Greece. Bei et al. recorded *Cunaxa mageei* from China. Bashir, Afzal, and Ali (2005) described *Cunaxa reticulatus* and moved *Rubroscirus valentis*, *Rubroscirus rasile*, and *Rubroscirus otiosus* to *Cunaxa*. Bashir and Afzal (2006) described *Cunaxa jatoiensis*. Sergeyenko (2009) described *Cunaxa gordeevae*, *Cunaxa guanotoleranta*, *Cunaxa maculata*, *Cunaxa papuliphora*, *Cunaxa violaphila* and *Cunaxa yaylensis*. Den Heyer and Sergeyenko (2009) redescribed *Cunaxa setirostris* and designated a neotype for the species. Bashir and Afzal (2009) described *Cunaxa bashiri*, *Cunaxa clusus*, *Cunaxa dotos*, *Cunaxa lodhranensis*, *Cunaxa mahmoodi*, *Cunaxa nankanaensis*, *Cunaxa okaraensis*, *Cunaxa pakpatanensis*. Bashir et al. (2010) described *Cunaxa rafiqi* and *Cunaxa leuros*. Bashir et al. (2011) “described” *Cunaxa nankanaensis* as a new species using the same illustrations Bashir and Afzal (2009) used to describe the species originally. Den Heyer et al. (2011a) described the male of *Cunaxa capreolus*.

#### Diagnosis.

*Gnathosoma*. **Pedipalps**–5-segmented and reach beyond the subcapitulum by at most the distal half of the tibiae. An apophysis on the telofemora present or absent. Dorsolateral setae on the basi- and telofemora simple. Stout spine-like setae on the genua and tibiotarsi present or absent. Tibiotarsi end in a strong claw. **Subcapitulum** with 6 pairs of setae: 2 pairs of adoral setae and 4 pairs of subcapitular setae (*hg_1–4_*). Subcapitulum smooth or patterned with random dots, but never reticulated.

*Idiosoma, dorsal*. Proterosoma bears a shield that is complemented with 2 pairs of setae (*at* and *pt*) and 2 pairs of setose sensillae (*lps* and *mps*). Dorsal hysterosoma may bear a shield; if a shield is present, it may bear up to 4 pairs of setae. Dorsal shields may be smooth or patterned with random dots, but never reticulated. Lateral platelets (as in *Armascirus* and *Dactyloscirus*) absent. Setae *c_1_*–*h_1_*, and *c_2_* present. Setae not born on the median plate may be born on small platelets that are barely larger than the setal socket. Cupule *im* present laterad and caudally of *e_1_*. Integument not bearing the proterosomal shield and median plate (if present) striated. These striations smooth or lobed but never papillated.

*Idiosoma, ventral*. **Coxae** I–II may be fused and coxae III–IV may be fused. Coxae II–IV setal formula 1-3-2. Genital plates each bear 4 setae; 2 pairs of genital papillae visible underneath the plates. Anal plates bear 1 pair of setae (*ps_1_*). 1 pair of setae (*h_2_*) associated with, but do not occur on, the anal plates. Cupule *ih* present in close proximity to *h_2_*. Integument between plates striated and bears up to 7 pairs of additional setae. **Legs.** Tarsi long and slender. Tarsi constricted distally but the tarsal lobes are small and not conspicuous as in *Armascirus* and *Dactyloscirus*. A trichobothrium on tibia IV present. Ambulacral claws on either side of a 4-rayed empodium present.

#### Key to adult female *Cunaxa*

*Cunaxa bochkovi* is not included in the key because the original description is in Cyrillic and the illustration does not contain enough detail or diagnostic characteristics. Den Heyer (pers. comm., Jan. 13, 2014) indicated that *Cunaxa setirostris* var. *plurisetosa* and *Cunaxa setirostris* var. *diversa* were described in “Mihelčič, F. 1958” but did not have the entire citation and had not seen the original description. The authors have also not been able to locate such a publication after extensive searching and so have not included the taxa here.

As suggested by Den Heyer (2011b), *Cunaxa boneti*, *Cunaxa denmarki*, *Cunaxa exoterica*, *Cunaxa floridanus*, *Cunaxa lehmanae*, *Cunaxa lukoschusi*, *Cunaxa metzi*, *Cunaxa myabunderensis*, *Cunaxa newyorkensis*, *Cunaxa rackae*, *Cunaxa reevesi*, and *Cunaxa reticulatus* are moved to *Rubroscirus* and *Cunaxa otiosus*,﻿ *Cunaxa valentis*, and *Cunaxa rasile* returned to *Rubroscirus* as they possess dorsal plates that are reticulated instead of smooth as in *Cunaxa*.

*Cunaxa nankanaensis* Bashir, Afzal, Ashfaq, Raza, Kamran, 2011 is considered a junior synonym and junior homonym of *Cunaxa nankanaensis* Bashir & Afzal, 2009.

**Table:** 

1	Setae *lps* present (Figs 54a–d)	2
–	Setae *lps* absent (Fig. 54e)	*Cunaxa anomala* Khaustov & Kuznetzov, 1998
2 (1)	Setae *at* normal, nearly as long as *pt*	3
–	Setae *at* short and stubby, less than half the length of *pt*	*Cunaxa anacardae* Gupta, 1992
3 (2)	Basifemora I with 1 sts	4
–	Basifemora I with 2 sts	5
–	Basifemora I with 3 sts	7
–	Basifemora I with 4 sts	14
–	Basifemora I with 5 sts	43
4 (3)	Basifemora I–IV setal formula 1-2-3-0; telofemora I–IV setal formula 2-2-4-3; India	*Cunaxa prinia* Gupta & Paul, 1985
–	Basifemora I–IV setal formula 1-1-1-2; telofemora I–IV setal formula 2-2-1-1; India	*Cunaxa magniferae* Gupta, 1992
5 (3)	Basifemora II-IV setal formula 2-1-0	6
–	Basifemora II-IV setal formula 3-3-1	*Cunaxa dotos* Bashir & Afzal, 2009
6 (5)	Tibia II with 5 sts; Pakistan	*Cunaxa mahmoodi* Bashir & Afzal, 2009
–	Tibia II with 7 sts; Pakistan	*Cunaxa okaraensis*
7 (4)	Genua I with 3 solenidia	8
–	Genua I with 4 solenidia	9
8 (7)	Genua II with 1 solenidion; setae *f_1_*, *h_1_* smooth (Fig. 55a)	*Cunaxa setirostris* (Hermann, 1804)
–	Genua II with 2 solenidia; setae *f_1_*, *h_1_* spiculate (Fig. 55b)	*Cunaxa magoebaensis* Den Heyer, 1979
9 (7)	Coxae I–IV setal formula 3-1-3-2 sts	10
–	Coxae I–IV setal formula 3-2-3-1 sts	*Cunaxa eupatoriae* Chinniah & Mohanasundaram, 2001
10 (9)	Dorsal setae short (*c_1_*–*f_1_*, *c_2_*: 7-10, *h_1_*: 17)	*Cunaxa mercedesae* Corpuz-Raros & Garcia, 1995
–	Dorsal setae longer (19-40)	11
11 (10)	Oval area formed by broken striae around setae *sci* present (Fig. 54a)	*Cunaxa maculata* Sergeyenko, 2009
–	Oval area formed by broken striae around setae *sci* absent (Fig. 54b)	12
12 (11)	Genua II proximal solenidion extremely short, its length subequal to the diameter of its alveolus; ventral surface of the coxal region of hypognathum smooth	*Cunaxa guanotoleranta* Sergeyenko, 2009
–	Genua II proximal solenidion long, its length several times longer than the diameter of its alveolus; ventral surface of the coxal region of the hypognathum with numerous papillae	13
13 (12)	Length of setae *sci* longer than half the distance between their bases; dorsal hysterosomal striae distinctly lobed (= with festoons) (Fig. 56a)	*Cunaxa papuliphora* Sergeyenko, 2009
–	Length of setae *sci* shorter or equal to half the distance between their bases; dorsal hysterosomal striae smooth (Fig. 56b)	*Cunaxa gordeevae* Sergeyenko, 2009
14 (3)	Basifemora III with 2 sts	15
–	Basifemora III with 3 sts	17
–	Basifemora III with 4 sts	41
15 (14)	Telofemoral apophysis uncinated (e.g., bent, hook-shaped) (Fig. 59a)	*Cunaxa jatoiensis* Bashir & Afzal, 2006
–	Telofemoral apophysis straight, not uncinated	16
16 (14)	Basifemora IV with 1 sts; cheliceral longitudinal striations present (Fig. 57a)	*Cunaxa heterostriata* Khaustov & Kuznetzov, 1998
–	Basifemora IV with 0 sts; cheliceral longitudinal striations absent (Fig. 57b)	*Cunaxa yaylensis* Sergeyenko, 2009
17 (14)	Basifemora IV with 0 sts	*Cunaxa violaphila* Sergeyenko, 2009
–	Basifemora IV with 1 sts	18
–	Basifemora IV with 2 sts	*Cunaxa brevicrura* Den Heyer, 1979
–	Basifemora IV with 5 sts	*Cunaxa meiringi* Den Heyer, 1979
18 (17)	Median plate present (may be indistinctly defined) (Figs 58a–e)	19
–	Median plate absent (Fig. 58f)	36
19 (18)	Telofemoral apophysis uncinated (e.g., bent, hook-shaped) (Fig. 59a)	20
–	Telofemoral apophysis present or absent; if present, not uncinated (Figs 59b–e)	25
20 (19)	Setae *c_1_* not on hysterosomal shield, on integument	21
–	Setae *c_1_* on hysterosomal shield	22
21 (20)	Tibiae I with 3 asl, 4 sts; Pakistan	*Cunaxa clusus* Bashir & Afzal, 2009
–	Tibiae I with 2 asl, 4 sts; Pakistan	*Cunaxa nankanaensis*
22 (20)	Setae *f_1_* on hysterosomal shield	23
–	Setae *f_1_* not on hysterosmal shield, on integument	24
23 (22)	Tibia III with 5 sts	*Cunaxa leuros* Bashir, Afzal, Ashfaq, Akbar & Ali 2010
–	Tibia III with 6 sts	*Cunaxa rafiqi* Bashir, Afzal, Ashfaq, Akbar & Ali 2010
24 (22)	Genua I with 2 asl, 5 sts	*Cunaxa capreolus* (Berlese, 1887)
–	Genua I with 3 asl, 3 sts; tibia I with 2 asl, 4 sts; Pakistan	*Cunaxa pakpatanensis*
–	Genua I with 3 asl, 4 sts; tibia I with 2 asl, 4 sts; Pakistan	*Cunaxa bashiri* Bashir & Afzal, 2009
25 (19)	Telofemoral apophysis truncated (Fig. 59b)	*Cunaxa carina* Den Heyer, 1979
–	Telofemoral apophysis not truncated (Figs 59c–e)	26
26 (25)	Line of small sharp spines on pedipalp tibiotarsi present (Fig. 60a)	*Cunaxa dentata* Sergeyenko, 2003
–	Line of small sharp spines on pedipalp tibiotarsi absent (Fig. 60b)	27
27 (26)	Median plate complemented with *c_2_* (Figs 58a–d)	28
–	Median plate not complemented with *c_2_* (Fig. 58e)	*Cunaxa terrula* Den Heyer, 1979
28 (27)	Median plate indistinctly defined (Fig. 58a)	29
–	Median plate distinctly defined (Fig. 58b–d)	30
29 (28)	Setae *f_1_*, *h_1_* smooth	*Cunaxa romblonensis* Corpuz-Raros & Garcia, 1995
–	Setae *f_1_*, *h_1_* finely setose	*Cunaxa sordwanaensis* Den Heyer, 1979
30 (28)	Median shield complemented with *c_1_*, *d_1_*, *c_2_* (Fig. 58b)	*Cunaxa sudakensis* Khaustov & Kuznetzov, 1998
–	Median shield complemented with *c_1_*–*e_1_*, *c_2_* (Fig. 58c, d)	31
31 (30)	Coxae IV with 1 sts	32
–	Coxae IV with 2 sts	33
32 (31)	Broken striae that form cell-like structures on median shield present (Fig. 58c)	*Cunaxa thailandicus* Smiley, 1992
–	Broken striae that form cell-like structures on median shield absent (Fig. 58d)	*Cunaxa veracruzana* Baker & Hoffmann, 1948
33 (31)	Setae *c_1_* longer than all other dorsal setae	*Cunaxa womersleyi* Baker & Hoffmann, 1948
–	Setae *c_1_* not longer than all other dorsal setae	34
34 (33)	Genua I–IV with 4-2-1-1 solenidia	*Cunaxa lamberti* Den Heyer, 1979
–	Genua I–IV with 3-1-1-1 solenidia	35
35 (34)	Setae *c_1_*–*h_1_* approximately equal in length	*Cunaxa hermanni* Den Heyer, 1979
–	Setae *c_1_*–*e_1_* half as long as *f_1_*, *h_1_*	*Cunaxa thessalica* Sionti & Papadoulis, 2003
36 (18)	Telofemoral apophysis uncinated (Fig. 59a)	37
–	Telofemoral apophysis not uncinated (Fig. 59b-e)	38
37 (36)	Genua I–IV setal formula 1 asl, 6 sts-7-6-6; Philippines	*Cunaxa pantabanganensis* Corpuz-Raros & Garcia, 1995
–	Genua I–IV setal formula 1 asl, 4 sts-5-6-6; Pakistan	*Cunaxa lodhranensis* Bashir & Afzal, 2009
38 (36)	Proterosomal shield striated (Fig. 54c)	39
–	Proterosomal shield smooth (Fig. 54d)	*Cunaxa potchensis* Den Heyer, 1979
39 (38)	Setae *f_1_*, *h_1_* smooth (Fig. 55a)	40
–	Setae *f_1_*, *h_1_* spiculate (Fig. 55b)	*Cunaxa gazella* (Ewing, 1913)
40 (39)	Pedipalp telofemoral apophysis short and cone-like (Fig. 59c)	*Cunaxa mageei* Smiley, 1992
–	Pedipalp telofemoral apophysis short and finger-like (Fig. 59d)	*Cunaxa neogazella*, Smiley, 1992
41 (14)	Median plate present (Fig. 58d); basifemora IV with 1 sts	*Cunaxa luzonica* Corpuz-Raros & Garcia, 1995
–	Median plate absent (Fig. 58f); basifemora IV with 1 or 2 sts	42
42 (41)	Basifemora IV with 1 sts	*Cunaxa cogonae* Corpuz-Raros & Garcia, 1995
–	Basifemora IV with 2 sts	*Cunaxa doxa* Chaudhri, 1980
43 (3)	Basifemora III with 4 sts	*Cunaxa evansi* Smiley, 1992
–	Basifemora III with 6 sts	*Cunaxa grobleri* Den Heyer, 1979

**Figures 54, 55.**
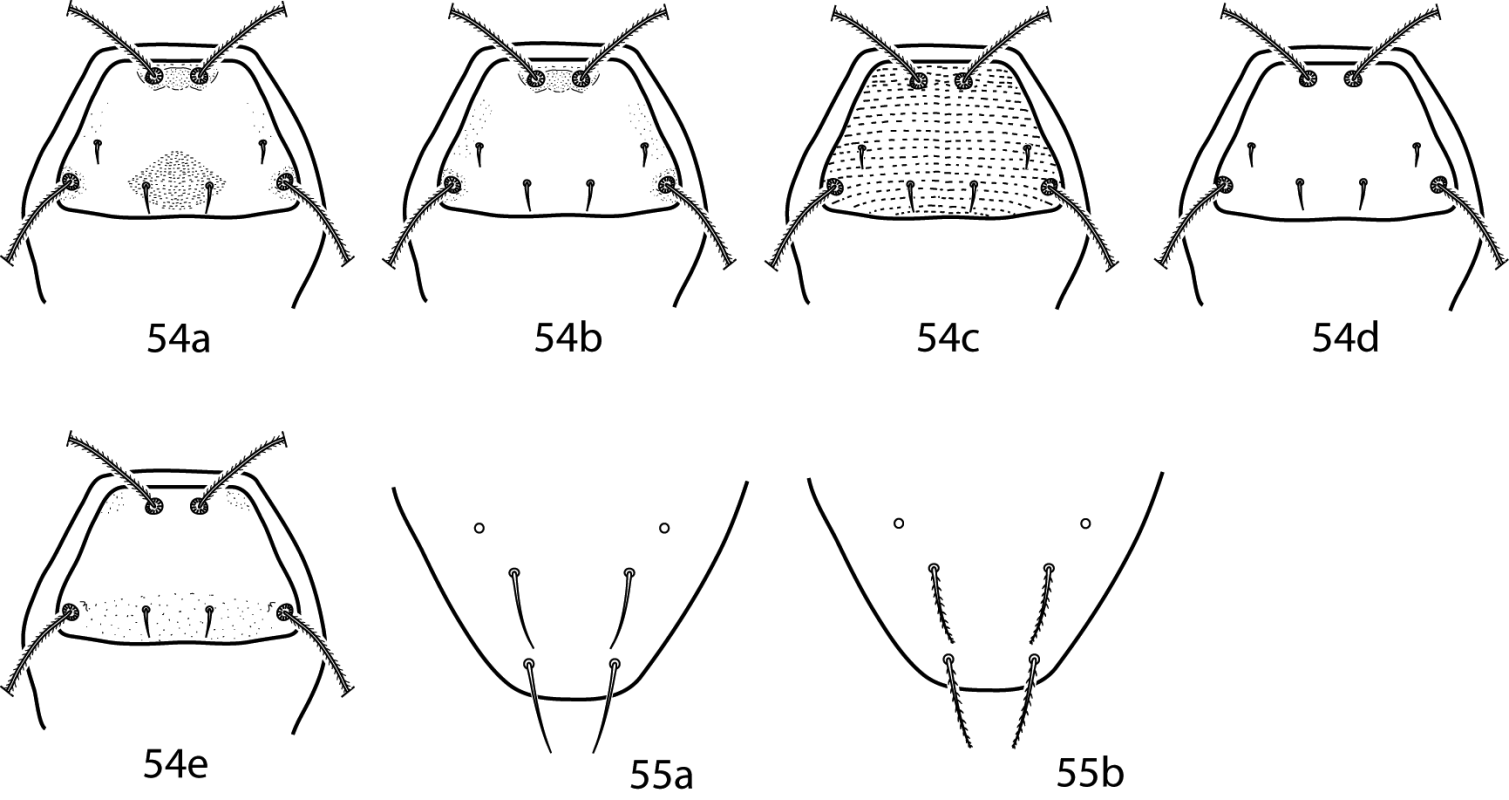
*Cunaxa* key illustrations. **54a–e** Proterosomal shield, dorsal **54a** Proterosomal shield with oval area formed by broken striae around *pt* present, *mps* present **54b** Proterosomal shield with oval area formed by broken striae around *pt* absent, *mps* present **54c** Proterosomal shield striated, *mps* present **54d** Proterosomal shield smooth, *mps* present **54e** Proterosomal shield with *lps* absent **55a** Smooth *f_1_*, *h_1_* **55b** Spiculate *f_1_*, *h_1_*.

**Figures 56–60.**
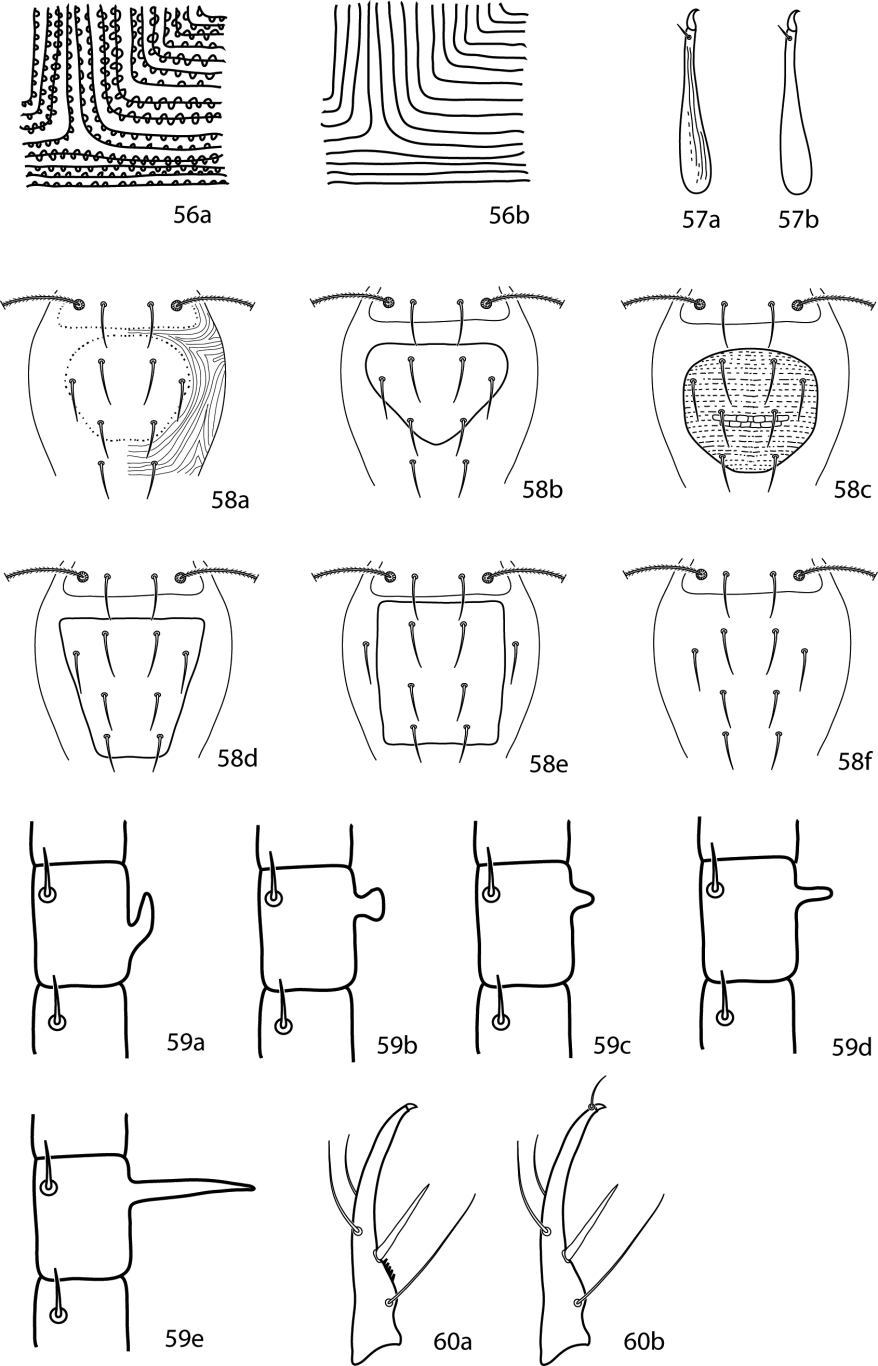
*Cunaxa* key illustrations. **56a, b** Integumental striations **57a** Chelicera with longitudinal striations present **57a** Chelicera with longitudinal striations absent **58a–f** Examples of variation in the hysterosomal median plate **59a** Pedipalp telofemoral apophysis uncinated **59b** Pedipalp telofemoral apophysis truncated **59c** Pedipalp telofemoral apophysis short and cone-like **59d** Pedipalp telofemoral apophysis short and finger-like **59e** Pedipalp telofemoral femoral apophysis long **60a** Pedipalp tibiotarsus with small teeth present **60b** Pedipalp tibiotarsus with small teeth absent.

### Cunaxatricha

Taxon classificationAnimaliaTrombidiformesCunaxidae

Castro & Den Heyer, 2008

#### Historical review.

Castro and Den Heyer (2008) erected *Cunaxatricha* for *Cunaxatricha tarsospinosa*.

#### Diagnosis.

*Gnathosoma*. **Pedipalps** 5-segmented and end in a strong claw. They extend beyond the subcapitulum by at least the last segment; apophyses absent. Basifemora complemented with a long simple seta; telofemora complemented with a short simple seta. These two segments fused, although a line remains visible and they can thus be differentiated. Subcapitulum complemented with 6 pairs of setae (*hg_1–4_* and 2 pairs of adoral setae). Setae *hg_4_* located between *hg_2–3_* instead of in the coxal region. **Chelicera** with seta present.

*Idiosoma, dorsal*. Female dorsal idiosoma bears a sclerotized shield that bears 2 pairs of setose sensillae (*at* and *pt*) and 2 pairs of simple setae (*lps* and *mps*). Idiosomal shield reticulated. 7 pairs of setae, *c_1–2_*, *d_1_–h_1_*, present. Cupule *im* present, usually posteriolaterad of *e_1_*. Integument striated.

*Idiosoma, ventral*. **Coxae** I and II fused, as are coxae III and IV. 6 pairs of setae present between and posterior to the coxae. Genital plates each bear 4 setae; 2 pairs of genital papillae not visible underneath the plates. Integument between plates striated and bears 4 pairs of additional setae. **Legs** shorter than the body. Leg 4 longest. Famulus on tarsi I normally shaped and set in a deep depression. Tarsi slightly constricted apically, resulting in small tarsal lobes. Basifemora and telofemora of legs I and II partially fused. A trichobothrium on leg tibia IV absent. Ambulacral claws on either side of a 4-rayed empodium present.

### Dactyloscirus

Taxon classificationAnimaliaTrombidiformesCunaxidae

Den Heyer, 1978

#### Historical review.

Trägårdh (1905) described *Scirus inermis*. Berlese (1916) erected *Dactyloscirus* as a subgenus of *Scirus* to accommodate *Scirus (Dactyloscirus) eupaloides*. He also described *Scirus dorcas* but failed to recognize that they were congeneric. Oudemans (1922) described *Rosenhofia machairodus*. Halbert (1923) redescribed and figured *Scirus inermis* from Ireland. Sellnick (1926) transferred *Scirus inermis* to *Cunaxa*. Vitzthum (1931) raised *Dactyloscirus* to full generic status but later (1940-43) treated it as a subgenus. Thor and Willmann (1941) again elevated *Dactyloscirus* to generic status and designated *Dactyloscirus eupaloides* as the type specimen; they also transferred *Cunaxa inermis* and *Scirus dorcas* to *Dactyloscirus*. Baker and Hoffmann (1948) regarded *Dactyloscirus* as a senior synonym of *Cunaxa*. Smiley (1975) synonymized *Rosenhofia* with *Dactyloscirus*. Zaher et al. (1975b) reported *Dactyloscirus inermis* from Egypt (though they called it *Cunaxa inermis*). Den Heyer (1978a) split *Armascirus* from *Dactyloscirus* and *Cunaxa* and raised the subfamily Cunaxinae to accommodate them, thus refining the definitions of all three genera. Den Heyer (1979a) described *Dactyloscirus condylus* and *Dactyloscirus dolichosetosus*. Den Heyer (1980c) erected the tribe *Armascirini* and made *Dactyloscirus* and *Armascirus* the sole representatives. Gupta and Ghosh (1980) described *Cunaxoides nicobarensis*. *Dactyloscirus pataliputraensis* was described by Gupta (1981). Liang (1986) described *Dactyloscirus humuli* from China. Shiba (1986) described *Dactyloscirus mesonotus*. Michocka (1987) reported *Dactyloscirus inermis* from Poland. Smiley (1992) transferred *Cunaxoides nicobarensis* to *Dactyloscirus* (though see discussion below) and described *Dactyloscirus mansoni*, *Dactyloscirus johnstoni*, and *Dactyloscirus poppi*. Gupta (1992) described *Dactyloscirus bengalensis*. Corpuz-Raros (1995) described *Dactyloscirus philippinensis*, *Dactyloscirus rosarioae*,﻿ and *Dactyloscirus agricolus*. Inayatullah and Shahid (1996) described *Dactyloscirus illutus*, *Dactyloscirus minys*,﻿ and *Dactyloscirus orsi*. Swift (1996) described *Dactyloscirus hoffmannae* and *Dactyloscirus smileyi* from the Hawaiian Islands. Hu (1997) reported *Dactyloscirus inermis* and *Dactyloscirus humuli* from China. Bashir and Afzal (2006a) described *Dactyloscirus imbecillus* and *Dactyloscirus manzoori*. Bashir, Afzal, and Akbar (2005) described *Dactyloscirus kahrorensis*. Corpuz-Raros (2008) described *Dactyloscirus discocondylus* and *Dactyloscirus trifidus*. Skvarla and Dowling (2012) described *Dactyloscirus pseudophilippinensis*. Den Heyer and Castro (2012) described *Dactyloscirus saopauloensis*.

#### Diagnosis.

*Gnathosoma*. **Pedipalps** 5-segmented, extend beyond the subcapitulum by at least the last segment, and end in a strong claw. An apophysis between the genua and tibiotarsi usually present. This apophysis long or short and generally ends in a bulbous, hyaline tip; it can, however, end in a tapering point as in *Armascirus*. This apophysis approximately equal between males and females or shorter in males. Basifemora and telofemora complemented with spine-like setae; these two segments fused, although a line remains visible and they can thus be differentiated. **Subcapitulum** complemented with 6 pairs of setae (*hg_1–4_* and 2 pairs of adoral setae) and covered by integumental papillae that are either randomly distributed or form a polygonal, reticulated pattern.

*Idiosoma, dorsal*. Female dorsal idiosoma has at least one sclerotized plate that bears 2 pairs of setose sensillae (*at* and *pt*) and 2 pairs of simple setae (*lps* and *mps*). 0–4 other major plates and platelets present. All plates, if present, covered by integumental papillae that form a reticulated pattern. Integument between plates striated. 7 pairs of setae (*c_1–2_*, *d_1_–h_1_*) present. Each seta, when not on a major plate or platelet, surrounded by a minute platelet only slightly larger than the setal socket. Cupule *im* present, usually laterad or in the proximity of *e_1_*. Dorsal idiosoma of males similar except a single large plate complemented with *c_1–2_*, *d_1_–e_1_* present.

*Idiosoma, ventral*. **Coxae** I and II often fused; coxae III and IV often fused. Setal formula for coxae I–IV 3-3-3-3 (including paracoxal seta). Genital plates each bear 4 setae; 2 pairs of genital papillae visible underneath the plates. Anal plates bear 1 pair of setae (*ps_1_*). 2 pairs of setae (*ps_2_* and *h_2_*) associated with, but do not occur on, anal plates. Cupule *ih* present in close proximity to *h_2_*. Integument between plates striated and bears 5–7 pairs of additional setae. Ventral idiosoma of males similar except the coxae much more extensive. A sclerotized aedeagus often visible in association with the genital plates. **Legs** comparatively short, generally not exceeding ¾ the length of the body. Famulus on tarsi I enlarged and ends in a tri-tipped prong. Tarsi constricted apically, resulting in large tarsal lobes. Trichobothrium on leg tibia IV present. Ambulacral claws occur on either side of a 4-rayed empodium.

#### Key to adult female *Dactyloscirus*

(modified from Skvarla and Dowling 2012)

Smiley (1992) transferred *Cunaxoides nicobarensis* to *Dactyloscirus* as *Dactyloscirus nicobarensis* (Gupta & Ghosh, 1980). However, later in the same work he attributes the same holotype (No. 3146/17) and same description (viz. Gupta and Ghosh 1980:191) to *Cunaxoides nicobarensis* Gupta & Ghosh, 1980. The original description and illustration by Gupta and Ghosh clearly state the species in question has three pedipalpal segments, which precludes it from being assigned to *Dactyloscirus*. Smiley illustrated a *Dactyloscirus* with 5-segmented pedipalp “after Gupta and Ghosh 1980” when discussing *Dactyloscirus nicobarensis*, though it looks like nothing in the publication. Because of this *Dactyloscirus nicobarensis* (Gupta and Ghosh 1980) is declared *nomen dubium*.

**Table:** 

1	Pedipalpal tibiotarsi and genua with adjoining apophyses present (Figs 61a–i)	2
–	Pedipalpal tibiotarsi and genua with adjoining apophyses absent (Figs 62a–d)	21
2 (1)	Dorsal hysterosomal lateral platelets present (Figs 63a–d)	3
–	Dorsal hysterosomal lateral platelets absent (Figs 64a–f)	15
3 (2)	Pedipalp telofemora with one or two apophyses (Figs 65a–c)	4
–	Pedipalp telofemora without an apophysis; distribution unknown	*Dactyloscirus poppi* Smiley, 1992
4 (3)	Pedipalpal telofemora with 1 apophysis (Figs 65a, b)	5
–	Pedipalpal telofemora with 2 apophyses: 1 basal, flattened and disc-shaped, 1 apical, short, thick and bulbous (Fig. 65c); South Africa	*Dactyloscirus condylus* Den Heyer, 1979
5 (4)	Lateral platelets inconspicuous, length less than 2 times the length of *c_1_* or *c_2_*; cosmopolitan (Fig. 63a)	*Dactyloscirus inermis* (Trägårdh, 1905)
–	Lateral platelets large, length greater than 2 times the length of *c_1_* or *c_2_* (Figs 63b–d)	6
6 (5)	Dorsal setae *f_1_* and *h_1_* equal in length; median shield present (Figs 63b, c) or absent (Fig. 63d)	7
–	Dorsal setae *f_1_* shorter than *h_1_*; median shield absent (Fig. 63d)	11
7 (6)	Apophysis adjoining pedipalpal genua and telofemora shorter than length of genu, blunt distally (Fig. 61a); median shield absent (Fig. 63d)	8
–	Apophysis adjoining pedipalpal genua and telofemora as long or longer than length of genu, blunt or pointed distally (Fig. 61c); median shield present or absent(Figs 63b, c)	10
8 (7)	Median shield present	9
–	Median shield absent; Japan	*Dactyloscirus mesonotus* Shiba, 1986
9 (8)	Coxa IV with 2 sts; Pakistan	*Dactyloscirus manzoori* Bashir & Afzal, 2006
–	Coxa IV with 3 sts; South Africa	*Dactyloscirus dolichosetosus* Den Heyer, 1979
10 (7)	Apophysis adjoining pedipalpal genua and telofemora pointed distally (Fig. 61b); pedipalp tibiotarsi with 4 sts; median shield complimented with setae *c_1_*, *d_1_*; *e_1_* on small platelets (Fig. 63b); leg basifemora with 5-5-3-1 sts; Luzon I., Philippines	*Dactyloscirus philippinensis* Corpuz-Raros, 1995
–	Apophysis adjoining pedipalpal genua and telofemora blunted distally (Fig. 61c); setae *c_1_*–*e_1_* on median shield (Fig. 63c); pedipalp tibiotarsi with 5 sts; leg basifemora with 5-5-3-2 sts; Ozark Mountains, USA	*Dactyloscirus pseudophilippinensis* Skvarla & Dowling, 2012
11 (6)	Apophysis adjoining pedipalpal genua and telofemora inconspicuous: circular, minute and hyaline (Fig. 61d); Oahu I., Hawaiian Islands	*Dactyloscirus hoffmannae* Swift, 1996
–	Apophysis adjoining pedipalpal genua and telofemora conspicuous, blunt apically (Fig. 61e)	12
12 (11)	Coxa IV with 2 sts	13
–	Coxae IV with 3 sts	14
13 (12)	Tibiae I with 1 asl, 4 sts; tibiae III with 1 asl, 5 sts	*Dactyloscirus kahrorensis* Bashir, Afzal & Akbar, 2006
–	Tibiae I with 2 asl, 4 sts; tibiae III with 2 asl, 4 sts	*Dactyloscirus imbecillus* Bashir & Afzal, 2006
14 (12)	Genital setae *g_3_* longest, 1.5–1.7 times the length of *g_2_* and *g_4_*, more than 2 times the length of *g_1_*; Kauai I., Hawaiian Islands	*Dactyloscirus smileyi* Swift, 1996
–	Genital setae *g_4_* longest, 2 times the length of *g_1–3_*; Shanghai, China	*Dactyloscirus humuli* Liang, 1986
15 (2)	Dorsal hysterosomal median shield present (Figs 64a–e)	16
–	Dorsal hysterosomal median shield absent (Fig. 64f)	18
16 (15)	Median shield complemented with *c_1_*, *d_1_* (Fig. 64b); apophysis adjacent to pedipalpal genua and tibiotarsi blunt distally (Fig. 61c); Mexico, Philippines	*Dactyloscirus mansoni* Smiley, 1992
–	Median shield complemented with *c_1_–e_1_* (Figs 64c, d); apophysis adjacent to pedipalpal genua and tibiotarsi blunt or pointed distally	18
–	Median shield complemented with *c_1_–e_1_*, *c_2_* (Fig. 64e); apophysis adjacent to pedipalpal genua and tibiotarsi pointed distally	*Dactyloscirus illutus* Inayatullah & Shahid, 1996
17 (18)	Apophysis adjacent to pedipalpal genua and tibiotarsi blunt distally (Fig. 61e); median shield triangular and nearly as wide as proterosomal shield (Fig. 64c); Bihar, India	*Dactyloscirus pataliputraensis* Gupta, 1981
–	Apophysis adjacent to pedipalpal genua and tibiotarsi tapering and pointed distally (Fig. 61f); median shield subrectangular and not as wide as proterosomal shield (Fig. 64d); Mexico	*Dactyloscirus johnstoni* Smiley, 1992
18 (17)	Pedipalpal telofemora without apophysis (Fig. 61g); apophysis adjoining pedipalpal genua and telofemora longer than telofemora and tapering to a point; Sumatra, Indonesia	*Dactyloscirus machairodus* (Oudemans, 1922)
–	Pedipalpal telofemora with 1 or 2 apophyses (Figs 65a–d); apophysis adjoining pedipalpal genu and telofemur shorter than telofemora and with a bulbus tip (Fig. 61a, d)	19
19 (18)	Pedipalpal telofemora with 1 apical apophysis (Figs 65a, b); apophysis adjoining genua and tibiotarsi larger (Fig. 61a)	20
–	Pedipalpal telofemora inner surface with 2 apophyses: 1 basal, flattened and disc-shaped, 1 apical, short, thick and bulbous (Fig. 65d); apophysis adjoining genua and tibiotarsi small, inconspicuous (Fig. 61d); Luzon I., Philippines	*Dactyloscirus discocondylus* Corpuz-Raros, 2008
20 (19)	Basal pair of adoral setae very long, more than 4 times the distal pair; pedipalp telofemoral apophysis about as long as width of segment (Fig. 65a); genital setae *g_4_* twice as long as *g_1_–g_3_*; Luzon I., Philippines	*Dactyloscirus rosarioae* Corpuz-Raros, 1995
–	Basal pair of adoral setae not unusually long, subequal to distal pair; pedipalp telofemoral apophysis short, less than width of segment (Fig. 65b); genital setae *g_4_* only slightly longer than *g_1_–g_3_*; Luzon I., Philippines	*Dactyloscirus agricolus*, Corpuz-Raros, 1995
21 (1)	Median shield present (Figs 64d, e)	22
–	Median shield absent (Fig. 64f)	23
22 (21)	Median shield complimented with *c_1_–e_1_* (Fig. 64d); Europe, North and South America	*Dactyloscirus eupaloides* Berlese, 1916
23 (21)	Coxa I with 2 sts; Pakistan	*Dactyloscirus bengalensis* Gupta, 1992
–	Coxa I with 3 sts	24
24 (23)	Pedipalp tibiotarsal claw trifid (Fig. 62c, d); coxa II–IV setal formula 3-3-3 sts; Luzon I., Philippines	*Dactyloscirus trifidus* Corpus-Raros, 2008
–	Pedipalp tibiotarsal claw entire, unbranched (Fig. 62a, b); coxa II–IV setal formula not as above	25
25 (24)	Coxal setal formula II–IV 1-3-2 sts; Peshawar, Pakistan	*Dactyloscirus orsi* Inayatullah & Shahid, 1996
–	Coxal setal formula II–IV 2-3-1 sts; Brazil	*Dactyloscirus saopauloensis* Den Heyer & Castro, 2012

**Figures 61–62.**
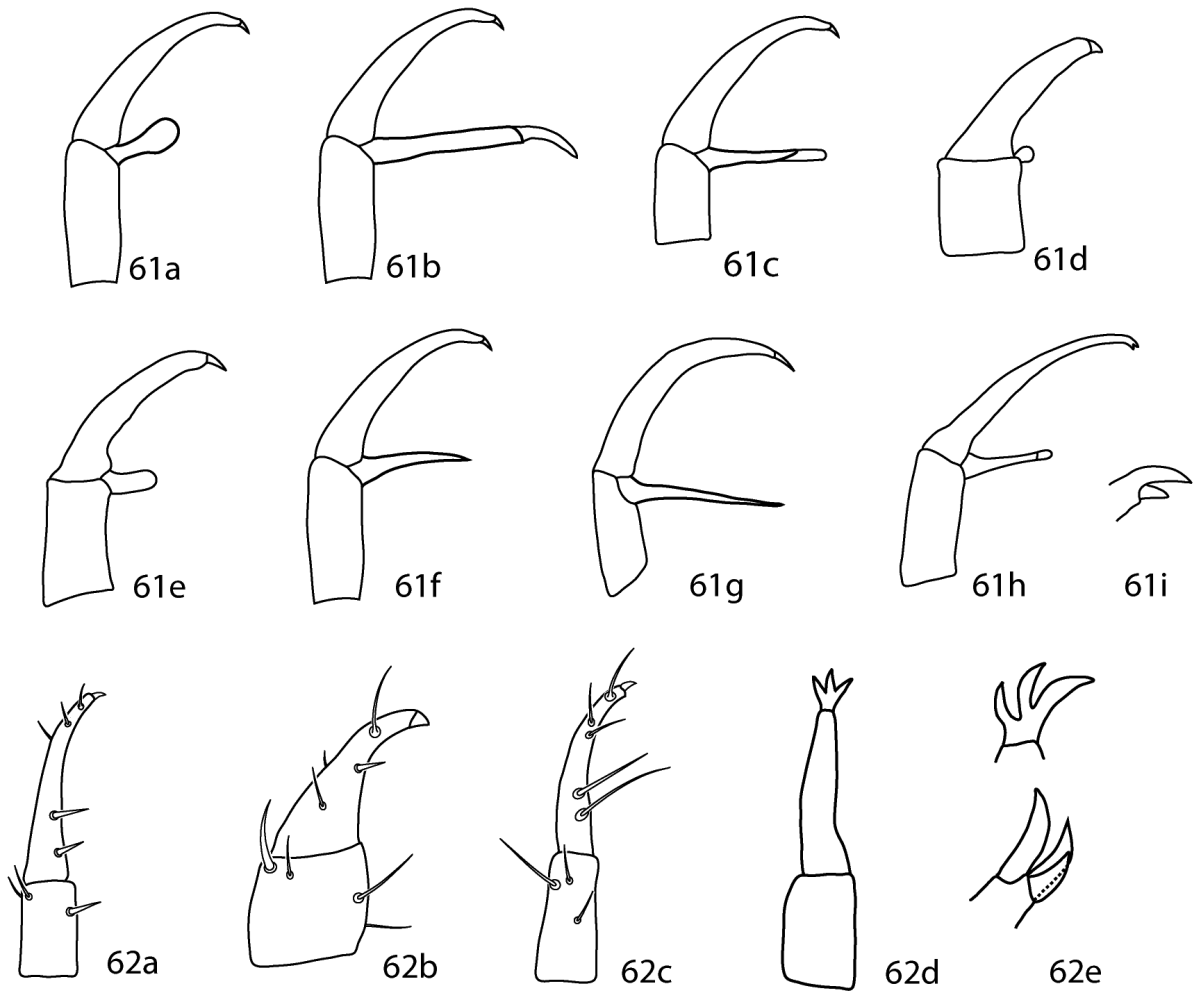
*Dactyloscirus* key illustrations. **61a–h** Pedipalp genu and tibiotarsus with adjoining apophysis present **61i** Close up of bifid claw **62a–d** Pedipalp genu and tibiotarsus with adjoining apophysis absent **62e** Close up of trifid claw.

**Figures 63–65.**
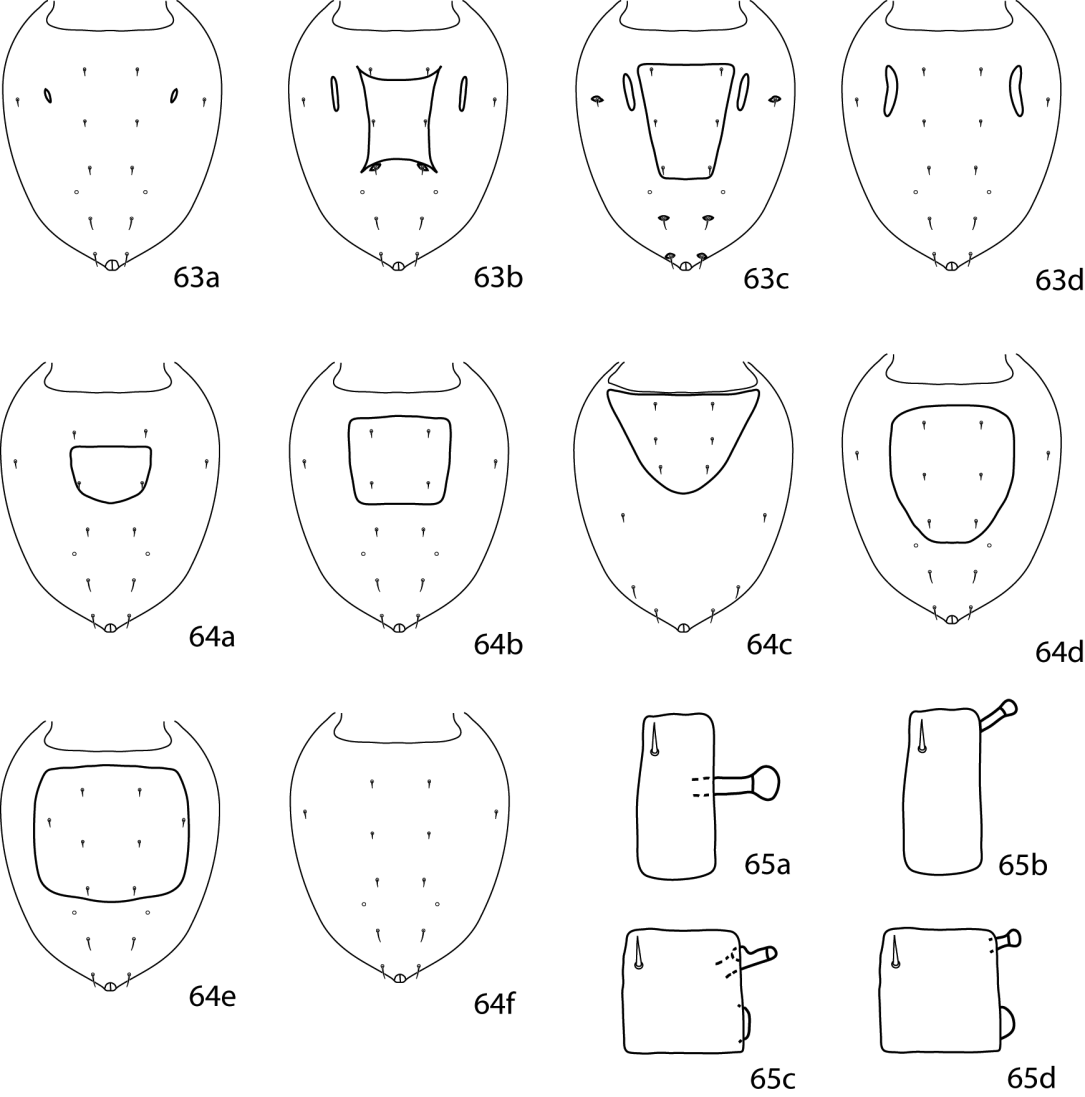
*Dactyloscirus* key illustrations. **63a–d** Dorsal idiosoma, lateral hysterosomal platelets present **64a–f** Dorsal idiosoma, lateral hysterosomal platelet absent **65a** Pedipalp telofemur with one apophysis, which is about as long as the width of the telofemur **65b** Pedipalp telofemur with one apophysis, which is shorter than the width of the telofemur **65c, d** Pedipalp telofemur with two apophyses, one apical and one basal which is flattened and disc-shaped.

### Riscus

Taxon classificationAnimaliaTrombidiformesCunaxidae

Den Heyer, 2006

#### Historical review.

Gupta and Ghosh (1980) described *Cunaxa bambusae*, *Cunaxa cynodonae* Den Heyer (2006) erected *Riscus* for *Riscus thailandensis*. Den Heyer (2011) transferred *Cunaxa bambusae* and *Cunaxa cynodonae* to *Riscus* based on the redescriptions by Corpuz-Raros (2008). Den Heyer and Castro (2012) described *Riscus austroamericanus*.

#### Diagnosis.

*Gnathosoma*. **Pedipalps** 5-segmented, extend beyond the subcapitulum by at least the last segment, and end in a strong claw; apophysis absent. Basifemora and telofemora complemented with simple setae; these two segments fused, although a line remains visible and they can thus be differentiated. **Subcapitulum** complemented with 6 pairs of setae (*hg_1–4_* and 2 pairs of adoral setae). Setae *hg_3_* and *hg_4_* both near the coxal bases of the pedipalps.

*Idiosoma, dorsal*. Female dorsal idiosoma has a sclerotized plate that bears 2 pairs of setose sensillae (*at* and *pt*) and 2 pairs of simple setae (*lps* and *mps*). Idiosomal shield covered by integumental papillae that form a reticulated pattern. Hysterosoma lacks a plate and bears 7 pairs of setae (*c_1–2_*, *d_1_–h_1_*). Cupule *im* present, usually laterad or in the proximity of *e_1_*.

*Idiosoma, ventral*. **Coxae** ill-defined. Coxae I and II fused; coxae III and IV fused. Coxae I–IV setal formula 3-1-3-1 (including paracoxal seta). Genital plates each bear 4 setae. Anal plates bear 1 pair of setae (*ps_1_*). 2 pairs of setae (*ps_2_* and *h_2_*) associated with, but do not occur on, the anal plates. Cupule *ih* present in close proximity to *h_2_*. Integument between plates striated and bears 5 pairs of additional setae. **Legs.** Ambulacral claws on either side of a 4-rayed empodium present.

#### Key to adult female *Riscus*

(modified from Den Heyer and Castro 2012)

**Table:** 

1	Five pairs of genital setae	*Riscus austroamericanus* Den Heyer & Castro, 2008
–	Four pairs of genital setae; tibiae IV with 1 T, 4 sts; tibiae II with {1 asl, 1 sts}, 4 sts	2
2 (1)	Pedipalpal genu with 3 sts	3
–	Pedipalpal genu with 4 sts	*Riscus bambusae* (Gupta & Ghosh 1980)
3 (2)	Pedipalpal tibiotarsus with 1 spls, 3 sts, 1 dorsoterminal solenidion	*Riscus thailandensis* Den Heyer, 2006
–	Pedipalpal tibiotarsus with 5 sts, 1 dorsoterminal solenidion (original description states 6 sts present; one of these is assumed to be a solenidion here)	*Riscus cynodonae* (Gupta & Ghosh, 1980)

### Rubroscirus

Taxon classificationAnimaliaTrombidiformesCunaxidae

Den Heyer, 1979

#### Historical review.

Baker and Hoffmann (1948) described *Cunaxa boneti*. Den Heyer (1979d) erected *Rubroscirus*, described *Rubroscirus africanus*, *Rubroscirus rarus*, and *Rubroscirus vestus*, and transferred *Cunaxa boneti* to the the genus. Tseng (1980) desbribed *Cunaxa exoterica*. Muhammad, Chaudhri, and Akbar (1989) described *Rubroscirus valentis*. Smiley (1992) synonymized *Rubroscirus* with *Cunaxa* and described *Cunaxa denmarki*, *Cunaxa floridanus*, *Cunaxa lehmanae*, *Cunaxa lukoschusi*, *Cunaxa metzi*, *Cunaxa newyorkensis*, *Cunaxa rackae*, and *Cunaxa reevesi*. Fan (1992) described *Rubroscirus denheyeri* and *Rubroscirus sinensis*. Muhammad and Chaudhri (1993) described *Rubroscirus rasile* and *Rubroscirus otiosus*. Corpuz-Raros and Garcia (1995) described *Cunaxa venusae* and *Cunaxa viscayana*. Bashir, Afzal, and Ali (2005) described *Cunaxa reticulatus* and transferred *Rubroscirus valentis*, *Rubroscirus rasile*, and *Rubroscirus otiosus* to *Cunaxa*. Sergeyenko (2006) recognized *Rubroscirus* as a valid genus and described *Rubroscirus khaustovi*. Ferla and Rocha (2012) described *Rubroscirus nidorum*.

#### Diagnosis.

*Gnathosoma*. **Pedipalps** 5-segmented and reach beyond the subcapitulum by at most the distal half of the tibiae. An apophysis on the telofemora present. Stout spine-like seta on the genua and tibiotarsi setae present or absent. Tibiotarsi end in a strong claw. **Subcapitulum** with 6 pairs of setae: 2 pairs of adoral setae and 4 pairs of subcapitular setae (*hg_1–4_*). Subcapitulum is reticulated.

*Idiosoma, dorsal*. Proterosoma bears a shield, complemented with 2 pairs of setose sensillae (*at* and *pt*) and 2 pairs of setae (*lps* and *mps*). Sensillae *at* and *pt* not as densely pilose as in *Allocunaxa*, *Cunaxatricha*, and *Riscus*. Proterosomal shield reticulated. Hysterosomal shield absent in females. Lateral platelets (as in *Armascirus* and *Dactyloscirus*) absent. Setae *c_1_*–*h_1_*, and *c_2_* present. Cupule *im* present laterad and caudally of *e_1_*. Integument not bearing the l shield striated. Striations papillated, not smooth or lobed as in *Cunaxa*.

*Idiosoma, ventral*. Coxae I–II may be fused; coxae III–IV may be fused. Coxae II–IV setal formula 1-3-1. Genital plates each bear 4 setae; 2 pairs of genital papillae visible underneath the plates. Anal plates bear 1 pair of setae (*ps_1_*). 1 pair of setae (*h_2_*) associated with, but do not occur on, the anal plates. Cupule *ih* present in close proximity to *h_2_*. Integument between plates striated and bears up to 7 pairs of additional setae. **Legs.** Tarsi long and slender, and constricted distally but tarsal lobes small and not conspicuous as in *Armascirus* and *Dactyloscirus*. A trichobothrium on tibia IV present. Ambulacral claws either side of a 4-rayed empodium present.

#### Key to adult female *Rubroscirus*

*Rubroscirus* is recognized as a valid genus. As suggested by Den Heyer (2011b) *Cunaxa boneti*, *Cunaxa denmarki*, *Cunaxa exoterica*, *Cunaxa floridanus*, *Cunaxa lehmanae*, *Cunaxa lukoschusi*, *Cunaxa metzi*, *Cunaxa newyorkensis*, *Cunaxa rackae*, *Cunaxa reevesi*, *Cunaxa reticulatus*, *Cunaxa venusae* and *Cunaxa viscayana* are transferred to *Rubroscirus* as they possess reticulated proterosomal shields.

**Table:** 

1	Basifemora I with 3 sts	2
–	Basifemora I with 5 sts	*Rubroscirus denmarki* (Smiley, 1992)
2 (1)	Basifemora III with 1 sts	3
–	Basifemora III with 2 sts; Pakistan	*Rubroscirus reticulatus* Bashir, Afzal & Ali, 2006
3 (2)	Basifemora IV with 1 sts	4
–	Basifemora IV with 2 sts; Mexico, Central America, USA	*Rubroscirus boneti* (Baker & Hoffmann, 1948)
4 (3)	Coxae I with 2 sts; Taiwan	*Rubroscirus exoterica* (Tseng, 1980)
–	Coxae I with 3 sts	5
5 (4)	Coxae II with 1 sts	6
–	Coxae II with 2 sts	16
6 (5)	Coxae IV with 1 sts	7
–	Coxae IV with 2 sts	12
7 (6)	Genua I with 1 asl, 5 sts; Ukraine	*Rubroscirus khaustovi* Sergeyenko, 2006
–	Genua I with 2 asl, 4 or 6 sts	8
–	Genua I with 3 asl, 5 or 6 sts	10
–	Genua I with 3 asl, 1 bsl, 5 sts; Pakistan	*Rubroscirus rasile* Chaudhri, 1993
8 (7)	Genua I with 2 asl, 4 sts; genua IV with 1 asl, 5 sts	9
–	Genua I with 2 asl, 6 sts; genua IV with 2 asl, 5 sts; USA	*Rubroscirus newyorkensis* (Smiley, 1992)
9 (8)	Genua II with 1 asl, 5 sts; China	*Rubroscirus denheyeri* Fan, 1992
–	Genua II with 1 asl, 6 sts; Brazil	*Rubroscirus nidorum* Ferla & Rocha, 2012
10 (7)	Genua I with 5 sts; genua II with 1 asl, 5 sts; USA	*Rubroscirus lehmanae* (Smiley, 1992)
–	Genua I with 6 sts; genua II with 2 asl, 5 or 6 sts	11
11 (10)	Genua II with 2 asl, 5 sts; Pakistan	*Rubroscirus valentis* Muhammad, Chaudhri & Akbar, 1989
–	Genua II with 2 asl, 6 sts; Pakistan	*Rubroscirus otiosus* Muhammad & Chaudhri, 1993
12 (6)	Genua I with 7 sts; Phillipines	*Rubroscirus viscayana* Corpuz-Raros & Garcia, 1995
–	Genua I with 2 asl, 5 or 6 sts	13
–	Genua I with 3 asl, 4 sts; China	*Rubroscirus sinensis* Fan, 1992
–	Genua I with 4 asl, 5 sts; USA	*Rubroscirus floridanus* (Smiley, 1992)
13 (12)	Genua I with 2 asl, 5 sts; genua II with 2 asl, 5 sts; genua IV with 1 asl, 5 sts	14
–	Genua I with 2 asl, 6 sts; genua II with 6 sts; genua IV with 6 sts; Philippines	*Rubroscirus venusae* Corpuz-Raros & Garcia, 1995
14 (13)	Genua III with 1 asl, 5 sts; setae c1, c2, d1, e1, f1, and h1 smooth	15
–	Genua III with 2 asl, 5 sts; setae c1, c2, d1, e1, f1, and h1 spiculate; Costa Rica	*Rubroscirus rackae* (Smiley, 1992)
15 (14)	Minute thorn-like seta adjacent to median spine-like seta on pedipalp tibiotarsus present; New Zealand	*Rubroscirus reevesi* (Smiley, 1992)
–	Minute thorn-like seta adjacent to median spine-like seta on pedipalp tibiotarsus absent; USA	*Rubroscirus metzi* Smiley, 1992
16 (5)	Basifemora I with 1 asl, 5 sts; basifemora II with 1 asl, 5 sts; basifemora III with 1 asl, 5 sts; basifemora IV with 1 asl, 5 sts; South Africa	*Rubroscirus africanus* Den Heyer, 1979
–	Basifemora I with 2 asl, 5 sts; basifemora II with 1 asl, 5 sts; basifemora III with 1 asl, 5 sts; basifemora IV with 2 asl, 5 sts	17
–	Basifemora I with 3 asl, 5 sts; basifemora II with 1 asl, 5 sts; basifemora III with 1 asl, 5 sts; basifemora IV with 1 asl, 5 sts; South Africa	*Rubroscirus vestus* Den Heyer, 1979
–	Basifemora I with 4 asl, 5 sts; basifemora II with 2 asl, 5 sts; basifemora III with 2 asl, 5 sts; basifemora IV with 1 asl, 5 sts; South Africa	*Rubroscirus rarus* Den Heyer, 1979
17 (16)	Setae c1, c2, d1, e1, f1, and h1 smooth; India	*Rubroscirus myabunderensis* (Gupta & Ghosh, 1980)
–	Setae c1, c2, d1, e1, f1, and h1 spiculate; Australia, Cominican Republic	*Rubroscirus lukoschusi* (Smiley, 1992)

### Coleoscirinae

Taxon classificationAnimaliaTrombidiformesCunaxidae

Den Heyer, 1978

#### Historical review.

Berlese (1888) described the first Coleoscirinae, *Scirus curtipalpus*, from Argentina. Berlese (1916) then erected *Coleoscirus* for two new species, *Coleoscirus halacaroides* and *Coleoscirus corniculatus* (*Coleoscirus corniculatus* was later synonomised with *Coleoscirus curtipalpus* by Den Heyer 1978b). Smiley (1975) erected *Pseudocunaxa* and *Pseudobonzia*. *Scutascirus* was erected by Den Heyer (1976) for a South African species, *Scutascirus polyscutosus*. Den Heyer (1977a) erected *Neoscirula* for three South African cunaxids. Den Heyer (1978b) synonymized *Pseudocunaxa* with *Coleoscirus* and erected Coleoscirinae for the known genera. Tseng (1980) erected *Lapicunaxa* for two species from Taiwan. Smiley (1992) moved *Neoscirula* from Coleoscirinae to Bonziinae, synomised *Lapicunaxa* with *Coleoscirus*, and erected *Neobonzia* in Neobonzinae. Den Heyer and Castro (2008b) erected *Coleobonzia* for some species previously contained in *Pseudobonzia*. Den Heyer and Castro (2008c) moved *Neoscirula* back to Coleoscirinae. Den Heyer (2011c) moved *Neobonzia* to Coleoscirinae, effectively disregarding Neobonzinae, and synonymized *Coleobonzia* with *Neobonzia*.

#### Diagnosis.

*Gnathosoma*. **Pedipalps** 5-segmented and reach beyond the subcapitulum by at most the distal half of the tibiotarsi. Basifemora and telofemora fused but retain a dark line. Tibiotarsi usually complemented with a tubercle and a dorsodistal solenidion. Pedipalps end in a stout claw. **Chelicera** with seta present or absent. **Subcapitulum** bears 6 pairs of setae: 2 pairs of adoral setae and 4 pairs of subcapitular setae (*hg_1_*_–_*_4_*). Setae *hg_4_* often longest.

*Idiosoma, dorsal*. Proterosoma covered in a shield which bears 4 pairs of setae: 2 pairs of simple setae (*lps* and *mps*) and 2 pairs of setose sensilla (*at* and *pt*). Dorsal hysterosoma median plate present or absent; if present this plate separate or fused to the proterosomal shield. Plates and shields smooth or variously covered with papillae that form reticulations. Up to 8 pairs of setae present on the dorsal hysterosoma (*c_1_*–*f_1_*, *c_2_*, *f_2_*, *h_2_*); if these setae do not occur on larger plates or shields they may be born on small platelets that are barely larger than the setal socket. Cupule *im* present, usually laterad or in the proximity of *e_1_*. Unsclerotized integument striated.

*Idiosoma, ventral*. **Coxae** I–II fused and may coalesce medially to form a single sternal plate. Each pair of coxae complemented with 3 pairs of setae; if they form an extensive sternal shield, setae normally born on the unsclerotized integument may be located on the shield. Coxae III–IV fused; they may be restricted to the trochantral bases or extend posteriorly beyond the genital plates. Each pair of coxae complemented with 3 pairs of setae; if the plates are extensive they may bear setae normally born on the unsclerotized integument. The genital plates each bear 4 setae; 2 pairs of genital papillae visible underneath the plates. 1–8 pairs of setae present on the integument between coxae III and the genital plates. Anal plates complemented with 2 pairs of setae (*ps_1-2_*). Two pairs of setae (*h_2_*, *pa*) located on the integument near the anal plates. Cupule *ih* present in close proximity to *h_2_*. **Legs** shorter than idiosoma; they are never constricted apically so as to end in lobes. Trichobothrium on leg tibia IV present. Ambulacral claws on either side of a four-rayed empodium present.

#### Key to adult female Coleoscirinae

(modified from Den Heyer and Castro 2008b)

**Table:** 

1	Idiosomal plates well-developed and defined; hysterosomal shield present and fused to proterosomal plate (Fig. 66a, b); females and most males with coxae I–II fused medially into a sternal shield (Fig. 67a); apices of some solenidia, especially on tarsi I, swollen	2
–	Idiosomal plates poorly developed and sometimes ill-defined; hysterosomal plate absent (Fig. 66c, d); coxae I–II usually not fused medially and restricted to trochantral bases (Fig. 67b, c); solenidia on tarsi I and II usually cylindrical	3
2 (1)	Idiosoma with 15 to 19 plates, including 4 pairs of dorsolateral plates (Fig. 66a); 2 dorsal plates; pedipalp tibiotarsal ventral tubercle often bifurcate (Fig. 68a)	*Scutascirus*
–	Idiosomal with no more than 8 plates; dorsolateral plates absent (Fig. 66b); females with only one dorsal plate but males with up to 3 dorsal plates; pedipalp tibiotarsal ventral tubercle not bifurcate, plain (Fig. 68b)	*Coleoscirus* Berlese, 1916
3 (1)	Pedipalp tibiotarsus short and nearly cone-like (Fig. 69a); cheliceral trochanters broad; ambulacral claws smooth	*Neoscirula* Den Heyer, 1977
–	Pedipalp tibiotarsus long and usually narrow and S-shaped (Fig. 69b); cheliceral trochanters narrow; ambulacral claws rippled	4
4 (3)	Subcuticular reticulated pattern present on proterosomal, coxal, and genital plates: usually very conspicuous, even proximal leg segments may possess such pattern (Fig. 67c)	Pseudobonzia Smiley, 1975
–	Subcuticular reticulated pattern absent or restricted to the edge of coxae (Fig. 67d)	*Neobonzia* Smiley, 1992

**Figures 66–69.**
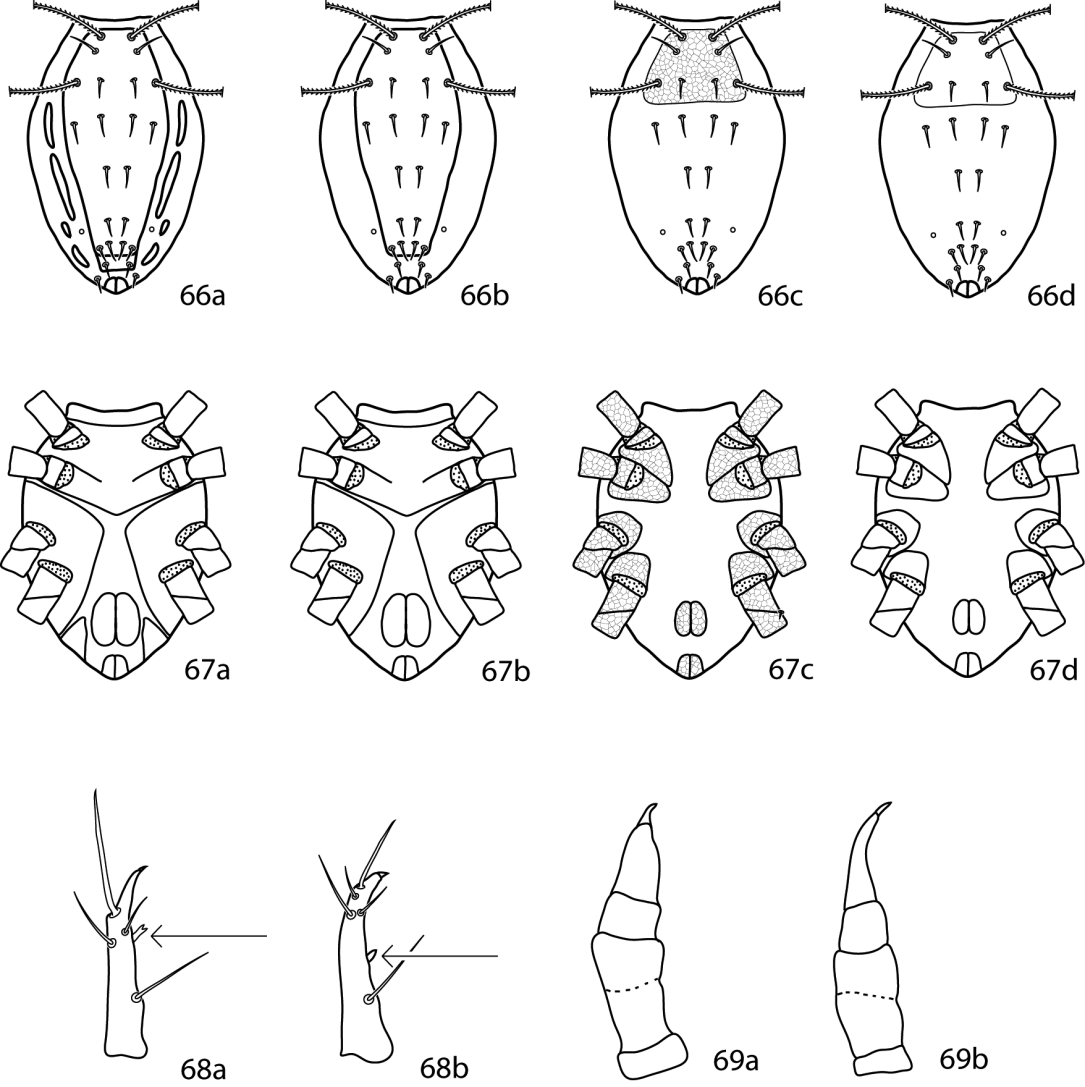
Cunaxoidinae key illustrations. **66a–d** Idiosoma, dorsal. Position of setae will vary between species. **67a–d** Idiosoma, ventral **66a, 67a** Generalized *Scutascirus*. Presence, position, and extent of lateral plates will vary between species **66b, 67b** Generalized *Coleoscirus* **66c, 67c** Generalized *Pseudobonzia* **66d, 67d** Generalized *Neobonzia* **68a** *Scutascirus* pedipalp tibiotarsus, arrow indicates bifurcate tubercle **68b** *Coleoscirus* pedipalp tibiotarsus, arrow indicates plan tubercle **69a** *Neoscirula* pedipalps with short, cone-like tibiotarsus **69b** *Pseudobonzia* and *Neobonzia* pedipalps with elongate, s-shaped tibiotarsus.

### Coleoscirus

Taxon classificationAnimaliaTrombidiformesCunaxidae

Berlese, 1916

#### Historical review.

Berlese (1916) erected *Coleoscirus* to accommodate two species, the type-species *Coleoscirus halacaroides* and *Coleoscirus corniculatus*. He had previously described two other species that would be assigned to the genus, *Scirus curtipalpus* (Berlese, 1888) and *Scirus brevicornis* (Berlese, 1905), but failed to recognize they belonged to *Coleoscirus*. Ewing (1917) described *Scirus simplex* from refuse hog hair in Illinois, USA. Thor and Willmann (1941) transferred *Scirus curtipalpus*, *Scirus brevicornis*, and *Scirus simplex* to *Cunaxa* and provided redescriptions and illustrations. Baker and Hoffmann (1948) described *Cunaxa mexicana*, as well as redescribing and illustrating *Cunaxa simplex*, *Coleoscirus curtipalpus*, and *Coleoscirus brevicornis*. Zaher et al. (1975b) reported *Coleoscirus simplex* from Egypt. Smiley (1975) provided an English translation of Berlese’s (1916) description of *Coleoscirus* but failed to include the genus in his key to genera; he also erected *Pseudocunaxa* for *Cunaxa simplex* and closely related species. Den Heyer (1978a) erected Coleoscirinae, designating *Coleoscirus* as the type genus and described *Coleoscirus magdalenae* and *Coleoscirus tuberculatus*; he also synonymized *Pseudocunaxa* with *Coleoscirus* and *Coleoscirus corniculatus* with *Coleoscirus curtipalpus*. Shiba (1978) described *Cunaxa mizunoi*. Tseng (1980) erected *Lapicunaxa horidula* and *Lapicunaxa monospinosus*. Chaudhri (1980) described *Pseudocunaxa baptus*. Den Heyer (1980b) described *Coleoscirus coatesi*, *Coleoscirus breslauensis*, and *Coleoscirus buartsus*, and synonymized *Coleoscirus magdalenae* with *Coleoscirus simplex*. Den Heyer (1980c) erected the tribes Coleoscirini for *Coleoscirus* and *Scutascirus* and Neoscirulini for *Neoscirula* and *Pseudobonzia*. Smiley (1992) synonymized *Lapicunaxa* with *Coleoscirus* and transferred *Cunaxa mizunoi* and *Pseudocunaxa baptus* to *Coleoscirus*; he also synonymized *Cunaxa mexicanus* with *Coleoscirus curtipalpus* and provided a key to known world species. *Coleoscirus carnus* and *Coleoscirus disparis* were described by Muhammad and Chaudhri (1992a). Inayatullah and Shahid (1993) described *Pseudocunaxa carex*, *Pseudocunaxa mardi*, and *Pseudocunaxa kifayati*, apparently unaware or ignoring that Den Heyer (1980) had synonymized *Pseudocunaxa* with *Coleoscirus* thirteen years earlier. Bu and Li (1987c) reported *Coleoscirus buartsus* from China. Corpuz-Raros (1996d) described six species of *Coleoscirus*: *Coleoscirus intermedius*, *Coleoscirus barrioni*, *Coleoscirus dayamilocus*, *Coleoscirus bakeri*, *Coleoscirus leytensis*, and *Coleoscirus philippinensis*. Hu (1997) reported *Coleoscirus monospinosus*, *Coleoscirus horidula*, and *Coleoscirus buartsus* from China. Bashir, Afzal, and Khan (2006) reaffirmed Den Heyer’s (1980) synonymization of *Pseudocunaxa* and *Coleoscirus* by treating *Pseudocunaxa carex*, *Pseudocunaxa mardi* and *Pseudocunaxa kifayati* as *Coleoscirus* and described *Coleoscirus trudus*; they also mention a second paper by Muhammad and Chaudhri (1992b) that described two additional species of *Coleoscirus* from Pakistan that I have been unable to obtain. Lin et al. (2003) reported *Coleoscirus simplex* from China. Fawzy (2007) described *Coleoscirus zaherii*. Bashir, Afzal, and Khan (2008) described *Coleoscirus raviensis* and *Coleoscirus tobaensis*. Bashir and Afzal (2009) described *Coleoscirus afzali*.

#### Diagnosis.

*Gnathosoma*. **Pedipalps** 5-segmented; basifemora and telofemora fused but retain a dark line which indicates the presence of the joint. Pedipalps extend beyond the subcapitulum by at most the apical half of the tibiotarsi. Pedipalp tibiotarsal tubercle plain, not bifurcate as in *Scutascirus*. **Subcapitulum** bears 6 pairs of setae: 2 pairs of adoral setae and 4 pairs of subcapitular setae (*hg_1_*_–_*_4_*).

*Idiosoma, dorsal*. Dorsal idiosoma heavily sclerotized and the plates well-demarcated. A single dorsal shield present; it may range in size from terminating anteriorly to cupule *im* to being holodorsal. No papillated line or other marking indicates the separation of the proterosomal and hysterosomal shields. 2 pairs of setae and 2 pairs of setose sensillae present on the proterosomal. Setae *c_1_*–*h_1_*, *c_2_*, and *f_2_* and cupule *im* present dorsally. Dorsolateral plates (such as present in *Scutascirus*) absent.

*Idiosoma, ventral*. **Coxae** I–II fused and coalesce medially to form a sternal shield which often has a prominent apex caudally. Sternal plate complemented with 5–7 pairs of setae. Coxae III–IV fused and may extend laterally and caudally past the genital plates. Genital plates each bear 4 setae; 2 pairs of genital papillae visible underneath the plates. Anal plates bear two pairs of setae (*ps_1_* and *ps_2_*). Seta *h_2_* located ventrally near the anal plates. Cupule *ih* present in close proximity to *h_2_*. **Legs** shorter than the idiosoma, never constricted apically so as to end in lobes. The apices of solenidia, especially on tarsi I, may be swollen. Trichobothrium on leg tibia IV present. Ambulacral claws on either side of a four-rayed empodium present.

Males similar, except up to three shields or plates may occur on the dorsal idiosoma (that is the proterosomal shield may not be fused to a hysterosomal plate and up to two hysterosomal plates may be present) and coxae I–IV may be fused into a holoventral shield.

#### Key to adult female *Coleoscirus*

*Coleoscirus brevicornis* (Berlese) has been excluded from the key as the original publication (Berlese 1904) and subsequent publication detailing the species (Thor and Willmann 1941) are in Italian and German and the accompanying illustrations provide too little detail. Den Heyer (1978b) is the last author to mention the species, but only indicates that it belongs to the genus *Coleoscirus*.

*Coleoscirus carex*, *Coleoscirus kifayati*, and *Coleoscirus mardi* have been excluded from the key as the authors did not provide enough information in the original descriptions to include them.

*Coleoscirus zaherii* is not included in the key as, despite the best efforts of the authors and the University of Arkansas Interlibrary Loan Department, the description could not be obtained.

**Table:** 

1	Basifemora I with 4 setae	2
–	Basifemora I with 5 setae	4
2 (1)	Basifemora II-IV setal formula 5-4-2	3
–	Basifemora II-IV setal formula 6-4-2; Pakistan	*Coleoscirus trudus* Bashir, Afzal & Khan, 2006
–	Basifemora II-IV setal formula 6-5-2; Pakistan	*Coleoscirus afzali* Bashir & Afzal, 2009
3 (2)	Telofemora I-IV setal formula 4-4-4-3; Pakistan	*Coleoscirus baptus* (Chaudhri, 1980)
–	Telofemora I-IV setal formula 4-5-4-3; Pakistan	*Coleoscirus raviensis* Bashir, Afzal & Khan, 2008
4 (1)	Basifemora II with 5 setae	5
–	Basifemora II with 6 setae	12
5 (4)	Basifemora III with 4 setae	6
–	Basifemora III with 5 setae	8
6 (5)	Basifemora IV with 2 setae	7
–	Basifemora IV with 3 setae; Java, South Africa	*Coleoscirus halacaroides* Berlese, 1916
7 (6)	Horizontal reticulations on dorsal shield present (Fig. 70); Taiwan	*Coleoscirus horidula* (Tseng, 1980)
–	Horizontal reticulations on dorsal shield absent; Taiwan	*Coleoscirus monospinosus* (Tseng, 1980)
8 (5)	Basifemora I-IV setal formula 4-5-3-3; Argentina	*Coleoscirus curtipalpus* (Berlese, 1888)
–	Basifemora I-IV setal formula not as above	9
9 (8)	Sternal shield bilobed posteriorly; Philippines	*Coleoscirus barrioni* Corpuz-Raros, 1996
–	Sternal shield not bilobed posteriorly	10
10 (9)	Extensive reticulations on gnathosoma present (Fig. 71); Philippines	*Coleoscirus bakeri* Corpuz-Raros, 1996
–	Extensive reticulations on gnathosoma absent	11
11 (10)	Hysterosomal shield present, complemented with *c_1_*-*f_1_*, *c_2_*, *f_2_*; Philippines	*Coleoscirus philippinensis* Corpuz-Raros, 1996
–	Hysterosomal shield absent; Philippines	*Coleoscirus intermedius* Corpuz-Raros, 1996
12 (4)	Basifemora III with 4 setae	13
–	Basifemora III with 5 setae	17
–	Basifemora III with 6 setae	20
13 (12)	Telofemora I-IV setal formula 4-4-4-3; USA, South Africa, Japan	*Coleoscirus simplex* (Ewing, 1917)
–	Telofemora I-IV setal formula 5-5-4-3	14
14 (13)	Setae *f_1_*, *f_2_* born on soft integument	15
–	Setae *f_1_*, *f_2_* born on dorsal shield; Pakistan	*Coleoscirus tobaensis* Bashir, Afzal & Khan, 2008
15 (14)	Sternal plate rounded posteriomedially (Figs 72a, b); South Africa	*Coleoscirus tuberculatus* Den Heyer, 1978
–	Sternal plate truncated posteriomedially (Fig. 72c)	16
16(15)	Light reticulation on dorsal shield present; dorsal shield evenly sclerotized (Fig. 73a); South Africa	*Coleoscirus buartsus* Den Heyer, 1980
–	Light reticulation on dorsal shield absent; dorsal shield unevenly sclerotized (Fig. 73b); South Africa	*Coleoscirus coatesi* Den Heyer, 1980
17(12)	Sternal shield indented posteriomedially (Fig. 72a); Malaysia	*Coleoscirus mizunoi* (Shiba, 1978)
–	Sternal shield not indented posteriomedially (Fig. 72b)	18
18 (17)	Setae *f_2_* born on soft integument; Pakistan	*Coleoscirus disparis* Muhammad & Chaudhri, 1992
–	Setae *f_2_* born on dorsal shield	19
19 (18)	Integumental dots on legs I-IV forming rows (Fig. 74a); Pakistan	*Coleoscirus carnus* Muhammad & Chaudhri, 1992
–	Integumental dots on legs I-IV forming random (Fig. 74b); South Africa	*Coleoscirus breslauensis* Den Heyer, 1980
20 (12)	Basifemora IV with 2 setae; Philippines	*Coleoscirus leytensis* Corpuz-Raros, 1996
–	Basifemora IV with 3 setae; Philippines	*Coleoscirus dayamilocus* Corpuz-Raros, 1996

**Figures 70–74.**
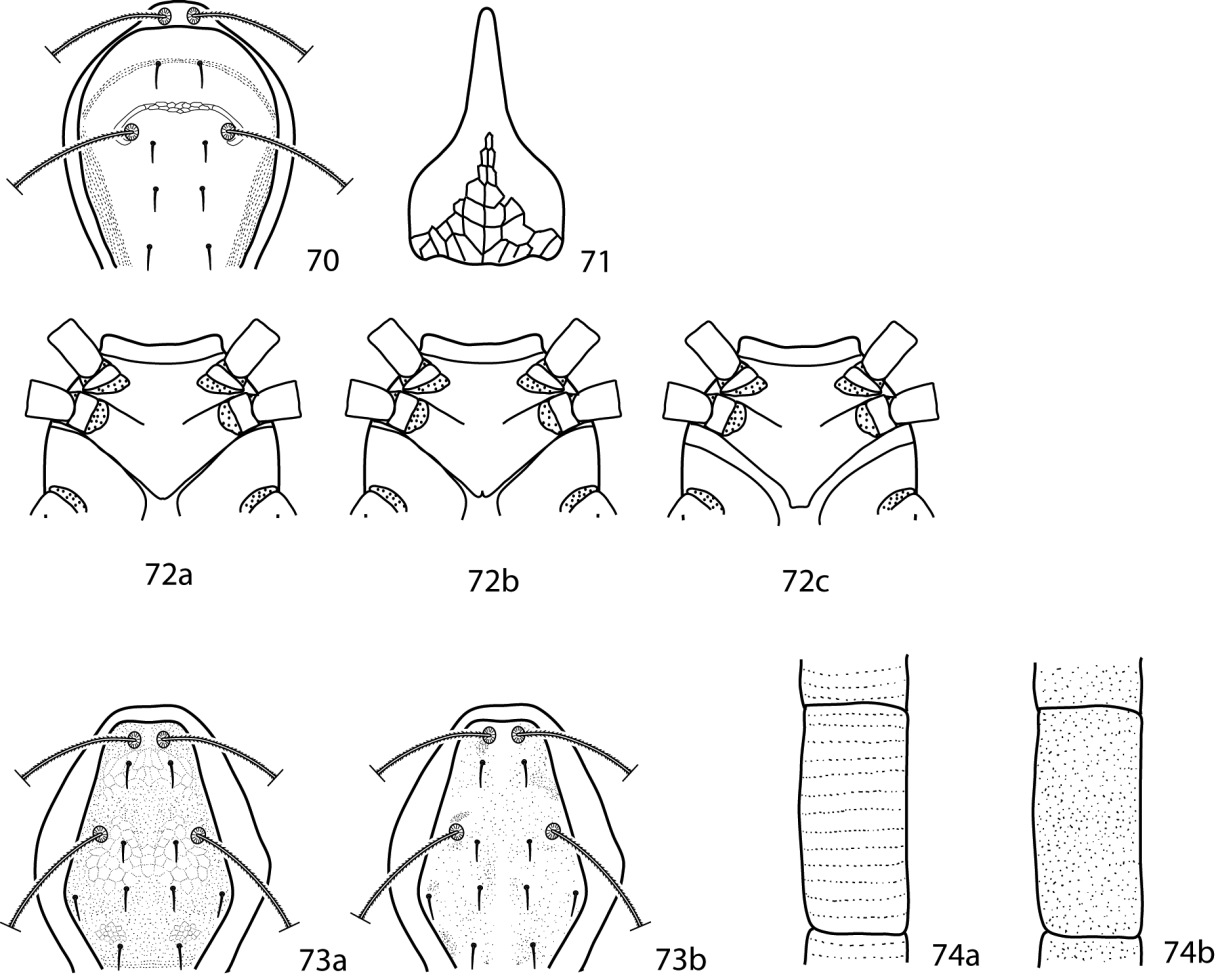
*Coleoscirus* key illustrations. **70** Dorsal idiosomal shield with horizontal reticulations present **71** Gnathosoma with extensive reticulations present **72a** Sternal plate rounded posteriomedially, indentation absent **72b** Sternal plate rounded posteriomedially, indentation present **72c** Sternal plate truncated posteriomedially **73a** Dorsal idiosomal shield even sclerotized, light reticulation present **73b** Dorsal idiosomal shield unevenly sclerotized, light reticulation absent **74a** Integumental dots on legs forming rows **74b** Integumental dots on legs random.

### Neobonzia

Taxon classificationAnimaliaTrombidiformesCunaxidae

Smiley, 1992

#### Historical review.

Berlese (1910) described the first species of *Neobonzia*, *Scirus parvirostris*. Thor and Willmann (1941) moved *Scirus parvirostris* to *Cunaxa*. Baker and Hoffmann (1948) described *Cunaxa snowi*. Heryford (1965) described *Cunaxa reticulata*. Smiley (1975) erected the genus *Pseudobonzia*, with *Cunaxa reticulata* as the type species. Den Heyer (1977c) redescribed *Pseudobonzia*, moved *Coleoscirus parvirostris* to *Pseudobonzia*, and described six new species from South Africa: *Pseudobonzia argillae*, *Pseudobonzia nona*, *Pseudobonzia lootsi*, *Pseudobonzia themedae*, and *Pseudobonzia saaymani*. *Pseudobonzia parilis* was described by Chaudhri (1977). Den Heyer (1980b) described *Pseudobonzia smileyi* and transferred *Cunaxa snowi* to *Pseudobonzia*. Chaudhri (1980) described *Pseudobonzia numida*. Luxton (1982) described *Pseudobonzia breviscuta* from New Zealand peat moss. Liang (1983) reported *Pseudobonzia themedae* from China. *Pseudobonzia shanghaiensis* was described by Liang (1984). Smiley (1992) described *Pseudobonzia newzealandicus*, *Pseudobonzia landwehri*, and *Pseudobonzia summersi*; reported *Pseudobonzia saaymani* from the USA and Canada; and erected a new monotypic subfamily, Neobonzinae, and genus, *Neobonzia*, for *Neobonzia moseri*. Corpuz-Raros and Garcia (1996) described two species from the Philippines, *Pseudobonzia gruezoi* and *Pseudobonzia longispina*. Hu (1997) reported *Pseudobonzia shanghaiensis* and *Pseudobonzia themedae* from China. Sergeyenko (2005) described *Pseudobonzia kuznetzovi*. *Pseudobonzia clavata* was described by Corpuz-Raros (2008). Den Heyer and Castro (2008b) split a new genus, *Coleobonzia*, from *Pseudobonzia*; They retained 6 speciesin *Pseudobonzia* (*Pseudobonzia clathratus*, *Pseudobonzia delfinadobaakerae*, *Pseudobonzia landwehri*, *Pseudobonzia neoreticulata*, *Pseudobonzia reticulata*, and *Pseudobonzia yini*) and transferred all other species to *Coleobonzia* and described *Coleobonzia clava* and *Coleobonzia moraesi*. Bashir and Afzal (2009) described *Pseudobonzia bakari*, *Pseudobonzia malookensis*, and *Pseudobonzia shamshadi*. Den Heyer (2011) synonymized *Coleobonzia* with *Neobonzia* and moved *Neobonzia* to Coleoscirinae, effectively disregarding Neobonzinae.

#### Diagnosis.

*Gnathosoma*. **Pedipalps** 5-segmented and reach beyond the subcapitulum by at most the distal half of the last segment. Simple setae present on the basi- and telofemora. Pedipalp tibiotarsi long and S-shaped (as opposed to short and cylindrical as in *Neoscirula*). **Subcapitulum** with 4 pairs of setae (*hg_1–4_*). 2 pairs of adoral setae present. **Chelicera** with seta usually present. Extensive reticulated pattern absent from the gnathosoma, though a row of single cells may be present caudally.

*Idiosoma, dorsal*. Plates lightly sclerotized and may not be well defined or demarcated. Proterosomal plate bears 2 pairs of setae (*lps* and *mps*) and 2 pairs of setose sensillae (*at* and *pt*). Extensive reticulated pattern absent, although a pair of rows of up to 6 cells may be present. Proterosomal plate may be covered with random dots or papillae. Hysterosomal plate absent. Setae *c_1_*–*h_1_*, and usually *c_2_* and *f_2_* present dorsally; *h_2_* present or absent. Cupules *im* present laterad and sometimes caudally of *e_1_*. Integument striated.

*Idiosoma, ventral*. **Coxae** usually restricted to the trochantral bases, though sometimes coxae I–II may nearly touch medially. Coxae I–II fused. Coxae III–IV fused. All coxae lightly sclerotized and may be ill-defined. Extensive reticulated pattern absent from the coxae, though a row of cells or reticulated pattern may be present near the edges. Coxae may be covered with random dots or papillae. Coxae I–IV usually have the simple setal formula 3-3-3-3 (*Neobonzia parilis* is the exception with 2-2-3-2). Genital plates each bear 3–4 setae; 2 pairs of genital papillae visible underneath the plates. 2 pairs of setae (*ps_1_*_–_*_2_*) usually occur on the anal plates and 1 pair of setae (*pa*) occurs on the integument near the anal plates. However, at least one species (*Neobonzia clavata*) has 3 pairs of setae present on the anal plates and 0 pairs of setae on the integument. Cupules *ih* present ventrally near the anal plates. **Legs.** Tarsi never constricted apically so as to end in lobes. The apices of solenidia cylindrical, not swollen as in *Coleoscirus* and *Scutascirus*. Trichobothrium on leg tibia IV present. Ambulacral claws rippled and occur on either side of a 4-rayed empodium.

#### Key to adult female *Neobonzia*

As suggested by Den Heyer (2013) *Pseudobonzia bakari*, *Pseudobonzia malookensis*, and *Pseudobonzia shamshadi* are transferred to *Neobonzia*.

*Neobonzia parvirostris* (Berlese, 1910) is known only from the male and so is not included in the key. *Neobonzia breviscuta* (Luxton, 1982) is not included in the key as an insufficient number of characters are given in the original description.

**Table:** 

1	Sensilla *at* and *pt* clavate (Figs 75a, b)	2
–	Sensilla *at* and *pt* not clavate, normal (Fig. 75c)	3
2 (1)	Sensilla *at* and *pt* short, length less than width of proterosomal plate (Fig. 75a); Philippines	*Neobonzia clavata* (Corpuz-Raros, 2008)
–	Sensilla *at* and *pt* long, length greater than width of proterosomal plate (Fig. 75b); Brazil	*Neobonzia clava* (Den Heyer & Castro, 2008)
3 (1)	Coxae I–IV setal formula 2-2-3-2 sts; Pakistan	*Neobonzia parilis* (Chaudhri, 1977)
–	Coxae I–IV setal formula 3-3-3-3 sts	4
4 (3)	Basifemora I with 2 sts	5
–	Basifemora I with 3 sts; Philippines	*Neobonzia longispina* (Corpuz-Raros & Garcia, 1996)
–	Basifemora I with 4 sts	7
–	Basifemora I with 5 sts	12
5 (4)	Basifemora II–IV setal formula 2-2-1 sts; USA	*Neobonzia moseri* Smiley, 1992
–	Basifemora II–IV setal formula 2-1-0 sts; Pakistan	*Neobonzia malookensis* (Bashir & Afzal, 2009)
–	Basifemora II–IV setal formula 3-3-1 sts	6
–	Basifemora II–IV setal formula 4-4-1 sts	*Neobonzia bakari* (Bashir & Afzal, 2009)
6 (5)	Telofemora I–IV setal formula 4-6-4-2 sts; China	*Neobonzia themedae* (Den Heyer, 1977)
–	Telofemora I–IV setal formula 5-5-4-3 sts; South Africa	*Neobonzia lootsi* (Den Heyer, 1977)
7 (4)	Basifemora II with 4 sts	8
–	Basifemora II with 5 sts	*Pseudobonzia shamshadi* (Bashir & Afzal, 2009)
–	Basifemora II with 6 sts	10
8 (7)	Basifemora III–IV setal formula 4-2 sts	9
–	Basifemora III–IV setal formula 6-1 sts; New Zealand	*Neobonzia newzealandicus* (Smiley, 1992)
9 (8)	Pedipalp tibiotarsal tubercle present; Brazil	*Neobonzia moraesi* (Den Heyer & Castro, 2008)
–	Pedipalp tibiotarsal tubercle absent; South Africa	*Neobonzia saaymani* (Den Heyer, 1977)
10 (7)	Basifemora III–IV setal formula 3-0 sts; South Africa	*Neobonzia nona* (Den Heyer, 1977)
–	Basifemora III–IV setal formula 3-1 sts; South Africa	*Neobonzia argillae* (Den Heyer, 1977)
–	Basifemora III–IV setal formula 4-2 sts	11
11 (10)	Setae *lps* and *mps* subequal; South Africa	*Neobonzia smileyi* (Den Heyer, 1980)
–	Setae *lps* about half as long as *mps*; USA	*Neobonzia summersi* (Smiley, 1992)
12 (4)	Basifemora II with 5 sts	13
–	Basifemora II with 6 sts	14
13 (12)	Coxae I–II nearly touching medially (Fig. 76a); USA, Austria	*Neobonzia snowi* (Baker & Hoffmann, 1948)
–	Coxae I–II widely separated medially (Fig. 76b); Philippines	*Neobonzia gruezoi* (Corpuz-Raros & Garcia, 1996)
14 (12)	Basifemora III–IV with 5-2 sts	15
–	Basifemora III–IV with 6-2 sts; Pakistan	*Neobonzia numida* (Chaudhri, 1980)
15 (14)	Setae *g_4_* longest; posterior corners of proterosomal shield angled; China	*Neobonzia shanghaiensis* (Liang, 1980)
–	Setae *g_3_* longest; posterior corners of proterosomal shield rounded; Russia	*Neobonzia kuznetzovi* (Sergeyenko, 2005)

**Figures 75, 76.**
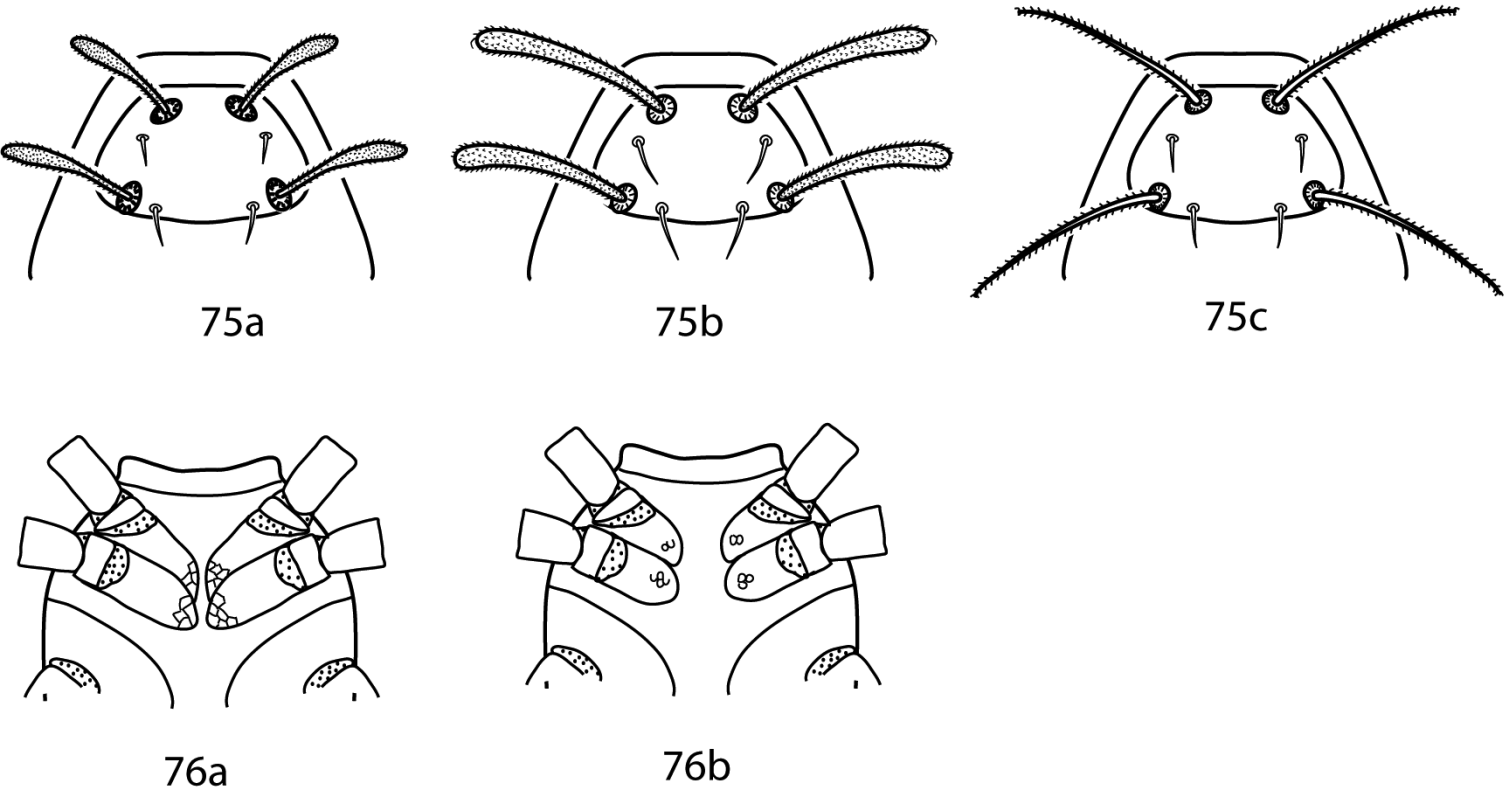
*Neobonzia* key illustrations **75a** Sensilla *at* and *pt* clavate, short, length less than the width of the proterosomal shield **75b** Sensilla *at* and *pt* clavate, long, length greater than the width of the proterosomal shield **75c** Sensilla *at* and *pt* normal, not clavate **76a** Coxae I–II nearly touching medially **76b** Coxae I–II widely separated medially.

### Neoscirula

Taxon classificationAnimaliaTrombidiformesCunaxidae

Den Heyer, 1977

#### Historical review.

Den Heyer (1977a) erected *Neoscirula* for three African cunaxids, *Neoscirula theroni*, *Neoscirula natalensis*, and *Neoscirula sevidi*. Shiba (1978) described the first *Neoscirula* outside of Africa, *Coleoscirus ogawai*. Den Heyer (1978b) erected the subfamily Coleoscirinae, tribus Neoscirulini and assigned *Neoscirula* to it. Den Heyer (1980b) described another African *Neoscirula*, *Neoscirula delareyi*. *Neoscirula vitulus* was described from Ukraine by Barilo (1991). Smiley (1992) transferred *Neoscirula* from Coleoscirinae to Bonziinae as he thought setae *g_1_* were geniculate; he also described *Neoscirula luxtoni*, *Neoscirula proctorae*, *Neoscirula kenworthyi*, moved *Neoscirula ogawai* from *Coleoscirus*, and provided a key to known world species. *Neoscirula abraensis*, *Neoscirula aspirasi*, *Neoscirula imperata*, *Neoscirula makilingica*, *Neoscirula puntiglupa* were described by Corpuz-Raros (1996e) from the Philippines. Lin and Zhang (1998) described *Neoscirula miaofengensis* and *Neoscirula bidens*. *Neoscirula saitoi* was described by Lin and Zhang (2002). Corpuz-Raros (2007) described two more Philippine *Neoscirula*: *Neoscirula laboensis*, *Neoscirula taclobanensis*. Mejía-Recamier and Palacios-Vargas (2007) described *Neoscirula aliciae*, *Neoscirula baloghi*, and *Neoscirula hoffmannae*. Den Heyer and Castro (2008c) described *Neoscirula flechtmanni*, *Neoscirula oliveirai*, and *Neoscirula queirozi*. Skvarla, Fisher, and Dowling (2011) described *Neoscirula reticulata*. Den Heyer (2011c) described *Neoscirula sepasgosariani*.

#### Diagnosis.

*Gnathosoma*. **Pedipalps** 5-segmented and end in a strong claw, which is complemented with a tooth in some species; they extend to the tip of the hypognathum or slightly beyond. Basifemur and telofemur are fused but retain the suture; each has a dorsolateral simple or spine-like seta. Pedipalp tibiotarsus short and cone-like. **Subcapitulum** with 4 pairs of setae (*hg_1_*_–_*_4_*). Seta *hg_1_* longest and in some species bent at 90 degrees, though not geniculate as in Bonziinae. Adoral setae present or absent. **Chelicera** with seta present or absent.

*Idiosoma, dorsal*. Proterosomal shield weakly sclerotized and ill-defined, granulated or papillated; some species possess subcuticular reticulations.

*Idiosoma, ventral*. **Coxae** I–II separate or fused medially into a single sternal shield. Coxae III–IV contiguous on either side, restricted to area around trochantral bases. Dorsal cupules *im* present laterad to *e_1_*; ventral cupules *ih* present near *h_2_*, anal plates. **Legs** shorter than body. Tarsi never constricted apically so as to end in lobes. Apices of solenidia cylindrical, not swollen as in *Coleoscirus* and *Scutascirus*. Trichobothrium on leg tibia IV present. Ambulacral claws smooth and occur on either side of a 4-rayed empodium.

#### Key to adult female *Neoscirula*.

*Neoscirula hoffmannae* Mejía-Recamier & Palacios-Vargas, 2007 is excluded from the following key as it is only known from the male.

**Table:** 

1	Coxae I–II fused to form a sternal shield	2
–	Coxae I–II separated	6
2 (1)	Cheliceral seta present	3
–	Cheliceral seta absent	5
3 (2)	Pedipalp basifemoral dorsal seta spine-like (Fig. 77a); Luzon Is., Philippines	*Neoscirula makilingica* Corpuz-Raros, 1996
–	Pedipalp basifemoral dorsal seta simple (Fig. 77b)	4
4 (3)	Proterosomal shield with polygonal subcuticular sculpturing present (Fig. 78a); posteromedial portion of sternal shield V-shaped, polygonal subcuticular sculpturing absent (Fig. 79a); 6 pairs of setae between coxae III–IV (excluding genital setae); Luzon Is., Philippines	*Neoscirula aspirasi* Corpuz-Raros, 1996
–	Proterosomal shield with polygonal subcuticular sculpturing absent (Fig. 78b); posteromedial portion of sternal shield rounded, polygonal subcuticular sculpturing present (Fig. 79b); 4 pairs of setae between coxae III–IV (excluding genital setae); Malaysia; Philippines	*Neoscirula ogawai* (Shiba, 1978)
5 (2)	Chelicerae with dorsomedial reticulations present (Fig. 80a); genua II with 5 setae and 2 solenidia; genua IV with 5 setae and 1 solenidion; Interior Highlands, USA	*Neoscirula reticulata* Skvarla, 2011
–	Chelicerae dorsomedial reticulations absent (Fig. 80b); genua II with 4 setae and 2 solenidia; genua IV with 4 setae and 1 solenidion; Jalisco, Mexico	*Neoscirula baloghi* Mejía-Recamier & Palacios-Vargas, 2007
6 (1)	Pedipalp genua hook-like apophysis present (Fig. 81a); South Africa	*Neoscirula natalensis* Den Heyer, 1977
–	Pedipalp genua hook-like apophysis absent (Fig. 81b)	7
7 (6)	Pedipalp tibiotarsal claw a tooth present, giving bifid appearance (Fig. 82a)	8
–	Pedipalp tibiotarsal claw a tooth absent (Fig. 82b)	13
8 (7)	Cheliceral seta present; pedipalp tibiotarsal tubercle present (Fig. 83a)	9
–	Cheliceral seta absent; pedipalp tibiotarsal tubercle absent (Fig. 83b); São Paulo, Brazil	*Neoscirula oliveirai* Den Heyer & Castro, 2008
9 (8)	Basifemora II with 4 setae; telofemora I–II 4-4 setae; hypognathum with ventroapical shield-like process present (Fig. 84a); New Zealand; Philippines	*Neoscirula luxtoni* Smiley, 1992
–	Basifemora II with 5 or 6 setae; telofemora I–II 5-5 setae; hypognathum with ventroapical shield-like process absent (Fig. 84b)	10
10 (9)	Basifemora II with 5 setae	11
–	Basifemora II with 6 setae	12
11 (10)	Basifemora I with 4 setae; telofemora III with 4 setae; 7 pairs of setae between coxae III–IV (excluding genital setae); Jalisco, Mexico	*Neoscirula aliciae* Mejía-Recamier & Palacios-Vargas, 2007
–	Basifemora I with 5 setae; telofemora III with 3 setae; 5 pairs of setae between coxae III–IV (excluding genital setae); Luzon Is., Philippines	*Neoscirula laboensis* Corpuz-Raros, 2007
12 (10)	Chelicerae tapering gradually (Fig. 85a); Fujian, China	*Neoscirula bidens* Lin & Zhang, 1988
–	Chelicerae tapering suddenly (Fig. 85b); São Paulo, Brazil	*Neoscirula flechtmanni* Den Heyer & Castro, 2008
13 (7)	Pedipalp basifemoral dorsal seta spine-like (Fig. 77a)	14
–	Pedipalp basifemoral dorsal seta simple (Fig. 77b)	18
14 (13)	Telofemora I–II with 4-4 setae; New Zealand	*Neoscirula proctorae* Smiley, 1992
–	Telofemora I–II with 5-5 setae	15
15 (14)	Proterosomal shield with polygonal subcuticular sculpturing present (Fig. 86a); Fujian, China	*Neoscirula saitoi*
–	Proterosomal shield with polygonal subcuticular sculpturing absent (Fig. 86b)	16
16 (15)	Cheliceral seta short, less than half the length of movable digit; South Africa	*Neoscirula sevidi* Den Heyer, 1977
–	Cheliceral seta long, nearly as long or longer than movable digit	17
17 (16)	Basifemora I–IV setal formula 5-5-4-3; Iran	*Neoscirula sepasgosariani* Den Heyer, 2011
–	Basifemora I–IV setal formula 4-4-3-1; Brazil	*Neoscirula queirozi* Den Heyer & Castro, 2008
18 (13)	Coxae I–II with polygonal subcuticular sculpturing present (as in Fig. 79a)	19
–	Coxae I–II with polygonal subcuticular sculpturing absent (as in Fig. 79b)	23
19 (18)	Proterosomal shield with polygonal subcuticular sculpturing present (Fig. 78a)	20
–	Proterosomal shield with polygonal subcuticular sculpturing absent (Fig. 78b)	21
20 (19)	Basifemora II with 4 setae; telofemora I–II 4-4 setae; Maryland, USA	*Neoscirula kenworthyi* Smiley, 1992
–	Basifemora II with 5 setae; telofemora I–II with 5-5 setae; Leyte Is., Philippines	*Neoscirula taclobanensis* Corpuz-Raros, 2007
21 (19)	Hypognathal setae *hg_1_* more than two times as long as setae *hg_2–4_*; coxae II with 4 setae; Fujian, China	*Neoscirula miaofengensis* Lin & Zhang, 1988
–	Hypognathal setae *hg_1_* no more than two times as long as setae *hg_2–4_*; coxae II with 3 setae	22
22 (21)	Chelicerae basally narrow, less than three times the width of the distal end; hypognathum narrow, nearly twice as long as wide; Uzbekistan	*Neoscirula vitulus* Barilo, 1991
–	Chelicerae basally broad, four times the width of the distal end; hypognathum wide, nearly as wide as long; South Africa	*Neoscirula delareyi* Den Heyer, 1980
23 (18)	Proterosomal shield with polygonal subcuticular sculpturing present	24
–	Proterosomal shield with polygonal subcuticular sculpturing absent; Luzon Is., Philippines	*Neoscirula imperata* Corpuz-Raros, 1996
24 (23)	Subcapitulum with row of basal polygonal subcuticular sculpturing present (Fig. 87a); ventrally with 7 pairs of simple setae between coxae III–IV	25
–	Subcapitulum with row of basal polygonal subcuticular sculpturing absent (Fig. 87b); ventrally with 6 pairs of simple setae between coxae III–IV; Luzon Is., Philippines	*Neoscirula abraensis* Corpuz-Raros, 1996
25 (24)	Basifemora II with 4 setae; telofemora I–II with 4-4 setae; Western Transvaal, South Africa	*Neoscirula theroni* Den Heyer, 1977
–	Basifemora II with 5 setae; telofemora I–II with 5-5 setae; Luzon Is., Philippines	*Neoscirula puntiglupa* Corpuz-Raros, 1996

**Figures 77–87.**
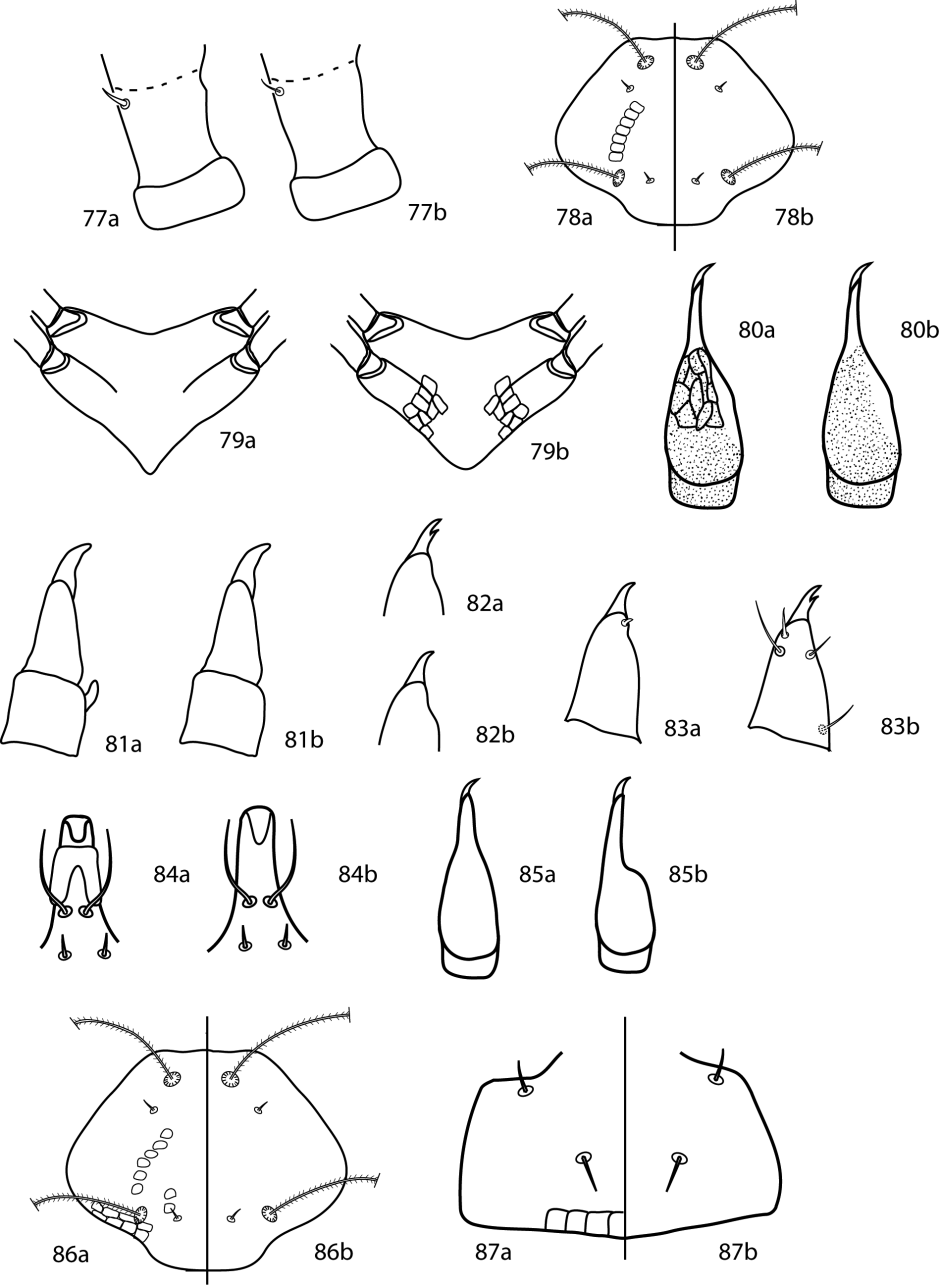
*Neoscirula* key illustrations **77a** Pedipalp basifemoral dorsal seta spine-like **77b** Pedipalp basifemoral dorsal seta simple **78a** Proterosomal shield with polygonal subcuticular sculpturing present **78b** Proterosomal shield with polygonal subcuticular sculpturing absent **79a** Sternal shield v-shaped posteriomedially, with polygonal subcuticular sculpturing absent **79b** Sternal shield rounded posteriomedially, with polygonal subcuticular sculpturing present **80a** Chelicera with dorsomedial reticulations present **80b** Chelicera with dorsomedial reticulations absent **81a** Pedipalp genua with hook-like apophysis present **81b** Pedipalp genua with hook-like apophysis absent **82a** Pedipalp tibiotarsal claw with tooth present **82b** Pedipalp tibiotarsal claw with tooth absent **83a** Pedipalp tibiotarsus with tubercle present **83b** Pedipalp tibiotarsus with tubercle absent **84a** Hypognathum with ventroapical shield-like process present **84b** Hypognathum with ventroapical shield-like process absent **85a** Chelicera tapering gradually **85b** Chelicera tapering suddenly **86a** Proterosomal shield with polygonal subcuticular sculpturing present **86b** Proterosomal shield with polygonal subcuticular sculpturing absent **87a** Subcapitulum with row of basal subcuticular sculpturing present **87b** Subcapitulum with row of basal subcuticular sculpturing absent.

### Pseudobonzia

Taxon classificationAnimaliaTrombidiformesCunaxidae

Smiley, 1975

#### Historical review.

Heryford (1965) described the first *Pseudobonzia*, *Cunaxa reticulata*. Smiley (1975) erected the genus *Pseudobonzia*, with *Cunaxa reticulata* as the type species. Den Heyer (1977c) redescribed the genus and described *Pseudobonzia neoreticulata*. Shiba (1978) described *Cunaxoides clathratus*. Smiley (1992) described *Pseudobonzia delfinadobakerae*, *Pseudobonzia landwehri*, and *Pseudobonzia yini* and moved *Cunaxoides clathratus* to *Pseudobonzia*; he also provided a key to known world species. Fuangarown and Lekprayoon (2004) described *Pseudobonzia tangkansingae*. Den Heyer and Castro (2008b) split *Coleobonzia* from *Pseudobonzia*. Bashir, Afzal, and Akbar (2008) described *Pseudobonzia ashfaqi*. Skvarla et al. (2013) reported *Pseudobonzia reticulata* from Arkansas and corrected the description to include setae *f_2_*, which were not reported by Heryford (1965).

#### Diagnosis.

*Gnathosoma*. **Pedipalps** 5-segmented and reach beyond the subcapitulum by at most the distal half of the last segment. Simple or spine-like setae on the basi- and telofemora present. Pedipalp tibiotarsi long and S-shaped (as opposed to short and cylindrical as in *Neoscirula*). **Subcapitulum** with 4 pairs of setae (*hg_1–4_*). 2 pairs of adoral setae present. **Chelicera** with seta present (usually) or absent. Extensive reticulated pattern present on the gnathosoma.

*Idiosoma, dorsal*. Plates lightly sclerotized and not be well defined or demarcated. The proterosomal plate bears 2 pairs of setae (*lps* and *mps*) and 2 pairs of setose sensillae (*at* and *pt*). Extensive reticulated pattern present. Hysterosomal plate absent. Setae *c_1_*–*h_1_* present; setae *c_2_*, *f_2_*, and *h_2_* present or absent. Cupules *im* present laterad and caudally of *e_1_*. Integument striated.

*Idiosoma, ventral*. **Coxae** restricted to the trochantral bases. Coxae I–II fused. Coxae III–IV fused. All coxae lightly sclerotized and may be ill-defined. Coxae with extensive reticulated pattern. Coxae I–IV usually have setal formula 3-3-3-3. Genital plates each bear 3–4 setae; 2 pairs of genital papillae visible underneath the plates. 2 pairs of setae (*ps_1_*_–_*_2_*) occur on the anal plates and 1 pair of setae (*pa*) occurs on the integument near the anal plates. Cupules *ih* present ventrally near the anal plates. **Legs.** Basal leg podomeres with reticulated pattern present or absent. Tarsi never constricted apically so as to end in lobes. Apices of solenidia cylindrical, not swollen as in *Coleoscirus* and *Scutascirus*. Trichobothrium on leg tibia IV present. Ambulacral claws are rippled and occur on either side of a 4-rayed empodium.

#### Key to adult female *Pseudobonzia*

(modified from Den Heyer and Castro 2008)

**Table:** 

1	Pedipalp basifemora and telofemora with similar setae, either spine-like or simple (Fig. 88a, b); proterosomal shield conspicuously reticulated	2
–	Pedipalp basifemora with simple seta, pedipalp telofemora with spine-like seta (Fig. 88c); proterosomal shield not conspicuously reticulated; Mexico	*Pseudobonzia delfinadobakerae* Smiley, 1992
2 (1)	Pedipalp basifemora and telofemora with simple setae (Fig. 88a); setae *f_2_* present or absent	3
–	Pedipalp basifemora and telofemora with spine-like setae (Fig. 88b); setae *f_2_* present; Guam	*Pseudobonzia yini* Smiley, 1992
3 (2)	Setae *f_2_* present	4
–	Setae *f_2_* absent	5
4 (3)	Proterosomal shield concave posteromedially (Fig. 89a); South Africa	*Pseudobonzia neoreticulata* Den Heyer, 1977
–	Proterosomal shield straight posteromedially (Fig. 89b); USA	*Pseudobonzia landwehri* Smiley, 1992
–	Proterosomal shield convex posteromedially (Fig. 89c); Pakistan	*Pseudobonzia ashfaqi* Bashir, Afzal & Akbar, 2008
5 (3)	Proximal leg podomeres reticulated; Malaysia	*Pseudobonzia clathratus* (Shiba, 1978)
–	Proximal leg podomeres not reticulated; USA	*Pseudobonzia reticulata* (Heryford, 1965)

**Figures 88, 89.**
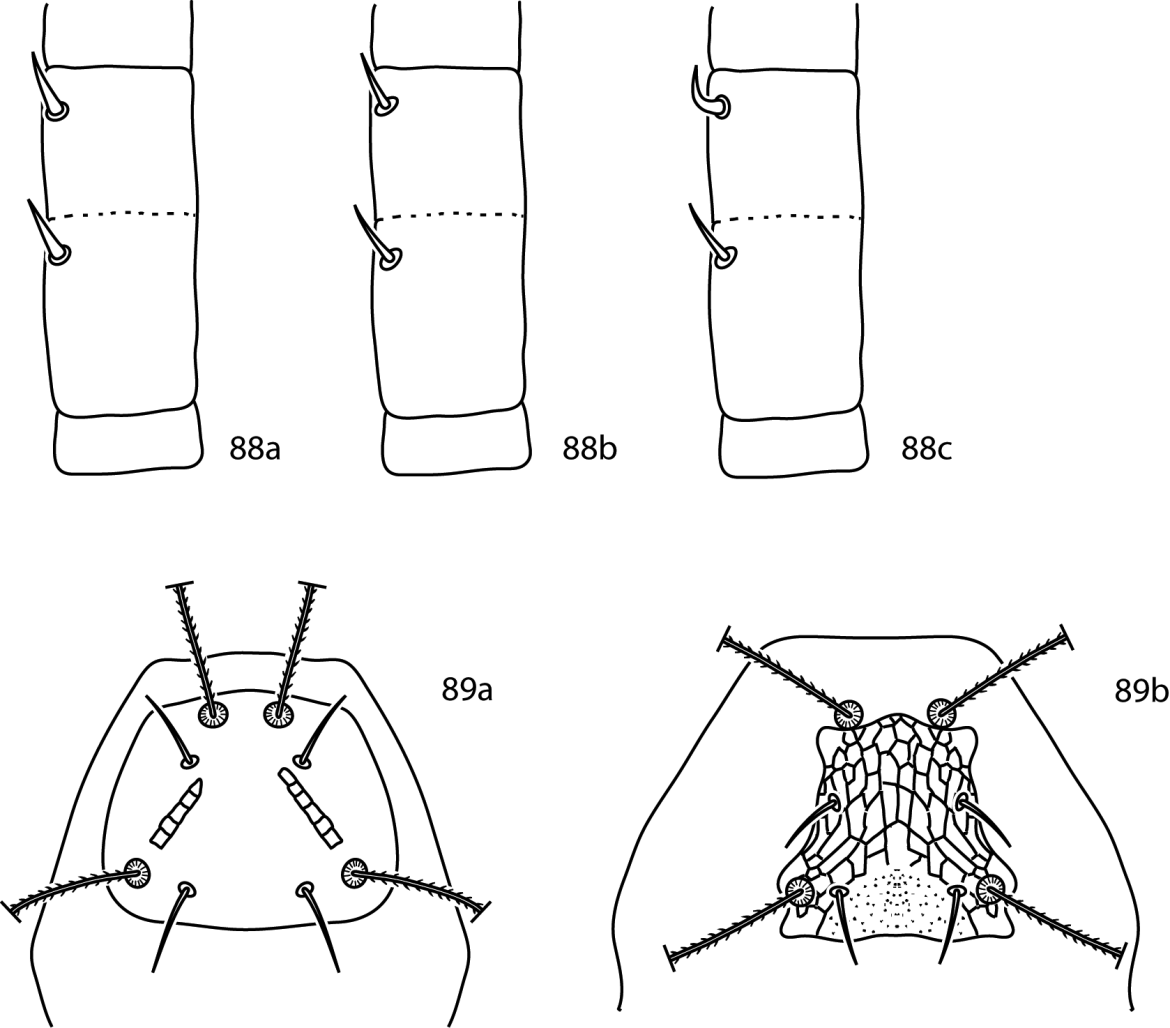
*Pseudobonzia* key illustrations **88a** Pedipalp basifemur and telofemur with spine-like setae on both segments **88b** Pedipalp basifemur and telofemur with simple setae on both segments **88c** Pedipalp with simple seta on basifemur, spine-like seta on telofemur **89a** Proterosomal plate convex posteriomedially **89b** Proterosomal plate not convex posteriomedially.

### Scutascirus

Taxon classificationAnimaliaTrombidiformesCunaxidae

Den Heyer, 1976

#### Historical review.

Den Heyer (1976) erected *Scutascirus* for *Scutascirus polyscutosus*. Shiba (1978) described *Cunaxa exasperatus*. Den Heyer (1980b) described *Scutascirus braziliensis*. Chaudhri (1980) described *Scutascirus pigrus*. Smiley (1992) transferred *Cunaxa exasperatus* to *Scutascirus*. Corpuz-Raros and Garcia (1996) described *Scutascirus contiguus* and *Scutascirus pentascutellus*. Lin, Zhang and Ji (2001) described *Scutascirus triangulum*.

#### Diagnosis.

*Gnathosoma*. **Pedipalps** 5-segmented and reach beyond the subcapitulum by at most the distal half of the tibiotarsi. Basifemora and telofemora fused but retain a dark line. The tibiotarsi complemented with a tubercle and a dorsodistal solenidion. Pedipalps end in a stout claw. **Chelicera** with seta present or absent. **Subcapitulum** bears 6 pairs of setae: 2 pairs of adoral setae and 4 pairs of subcapitular setae (*hg_1_*_–_*_4_*). Setae *hg_4_* often the longest.

*Idiosoma, dorsal*. Proterosoma covered in a shield which bears 4 pairs of setae: 2 pairs of simple setae (*lps* and *mps*) and 2 pairs of setose sensilla (*at* and *pt*). Dorsal hysterosoma bears a median plate which is fused with the proterosomal shield and four pairs of lateral platelets. Plates and shields covered with papillae that form reticulations. 8 pairs of setae present on the dorsal hysterosoma (*c_1_*–*f_1_*, *c_2_*, *f_2_*, *h_2_*); these setae occur on the fused dorsal shield. Cupule *im* present, usually laterad or in the proximity of *e_1_*. Unsclerotized integument striated.

*Idiosoma, ventral*. **Coxae** I–II fused and coalesce medially to form a single sternal plate. Each pair of coxae complemented with 3 pairs of setae; if they form an extensive sternal shield setae normally born on the unsclerotized integument may be located on the shield. Coxae III–IV fused and extend posteriorly beyond the genital plates. Genital plates each bear 4 setae; 2 pairs of genital papillae visible underneath the plates. 1–8 pairs of setae present on the integument between coxae III and the genital plates. Anal plates complemented with 2 pairs of setae (*ps_1-2_*). Two pairs of setae (*h_2_*, *pa*) located on the integument near the anal plates. Cupule *ih* present in close proximity to *h_2_*. **Legs** shorter than idiosoma. Tarsi never constricted apically so as to end in lobes. Trichobothrium on leg tibia IV present. Ambulacral claws on either side of a four-rayed empodium present.

#### Key to adult female *Scutascirus*

*Scutascirus tactus* is not included in the following key as it is described only from the male.

**Table:** 

1	Tubercle on inner margin of pedipalp tibiotarsus not branched (Fig. 90a)	2
–	Tubercle on inner margin of pedipalp tibiotarsus bifurcate (Figs 90b, c)	5
–	Tubercle on inner margin of pedipalp tibiotarsus trifurcate (Fig. 90d); China	*Scutascirus triangulum* Lin, Zhang & Ji, 2001
2 (1)	Telofemora III-IV setal formula 4-3	5
–	Telofemora III-IV setal formula 5-2; Philippines	*Scutascirus contiguus* Corpuz-Raros & Garcia, 1996
3 (2)	Genua II with 1 asl, 5 sts; dorsum with lateral scutella absent; Pakistan	*Scutascirus pigrus* Chaudhri, 1980
–	Genua II with 2 asl, 1 bsl, 5 sts; dorsum with lateral scutella present; Malaysia	*Scutascirus exasperatus* (Shiba, 1978)
4 (1)	Basifemora I–IV setal formula 4-6-4-2; Telofemora I–IV setal formula 5-5-4-3; 4 pairs of dorsolateral hysterosomal plates present (Fig. 91a)	5
–	Basifemora I–IV setal formula 5-5-4-3; Telofemora I–IV setal formula 5-5-5-2; 5 pairs of dorsolateral hysterosomal plates present (Fig. 91b); Luzon Is., Philippines	*Scutascirus pentascutellus* Corpuz-Raros & Garcia, 1996
5 (4)	Pedipalp with entire tibiotarsus projecting past entomalae; bifurcate tubercle positioned halfway along the length of the tibiotarsus (Fig. 90b); Brazil	*Scutascirus braziliensis* Den Heyer, 1978
–	Pedipalp with distal 2/3 of tibiotarsus projecting past entomalae; bifurcate tubercle positioned on distal third of tibiotarsus (Fig. 90c); South Africa	*Scutascirus polyscutosus* Den Heyer, 1976

**Figures 90, 91.**
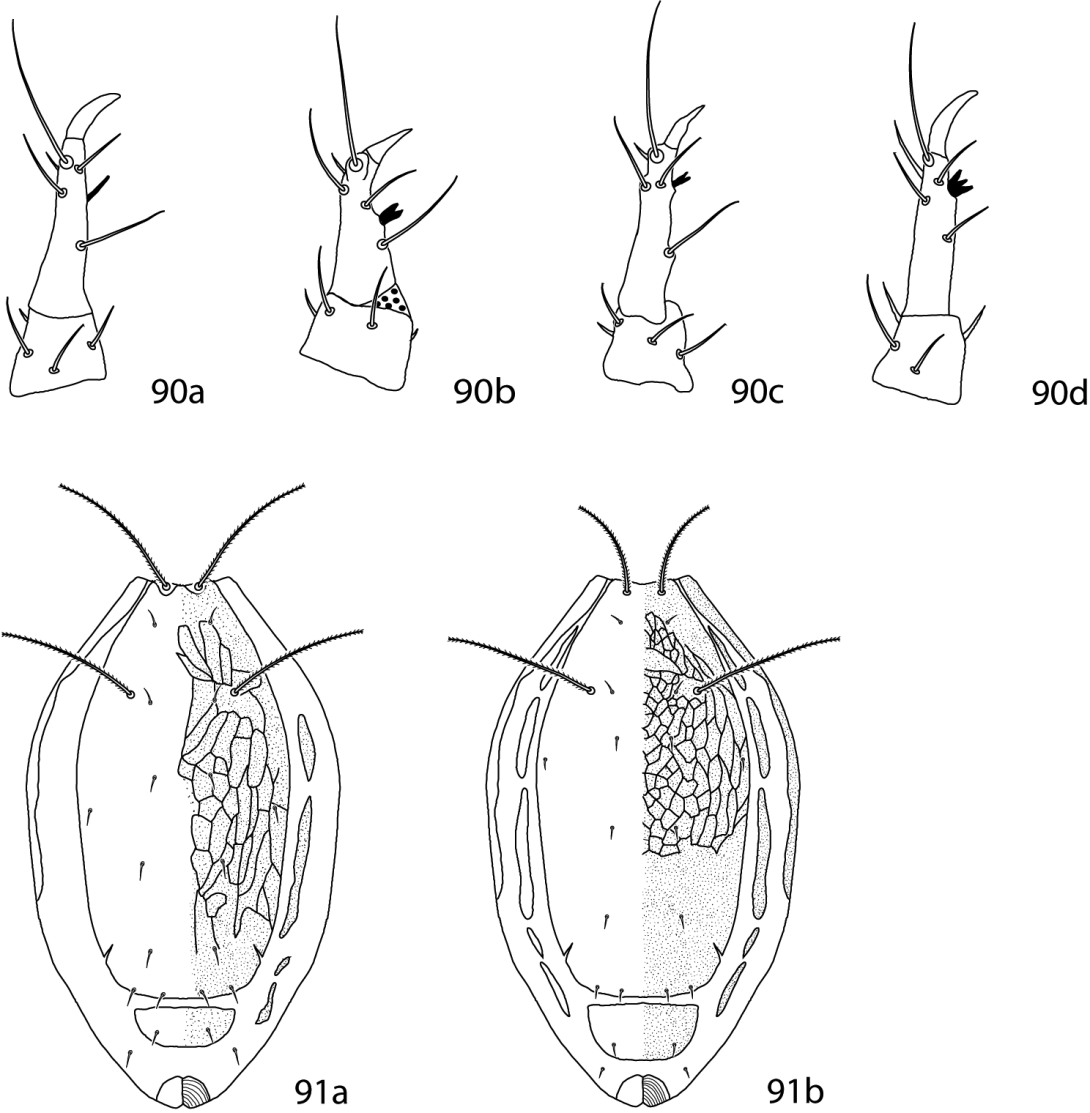
*Scutascirus* key illustrations. **90a** (after Corpuz-Raros and Garcia 1996). Pedipalp with tubercle not branched **90b** (after Den Heyer 1980b). Pedipalp tibiotarsus with bifurcate tubercle positioned halfway along the length of the segment **90c** (after Den Heyer 1980b). Pedipalp tibiotarsus with bifurcate tubercle positioned on distal third of segment **90d** (after Lin et al. 2001). Pedipalp tibiotarsus with trifurcate tubercle **90a** (after Den Heyer 1980b). Four pairs of dorsolateral hysterosomal plates present **91b** (after Corpuz-Raros and Garcia 1996). Five pairs of dorsolateral hysterosomal plates present.

### Orangescirlinae Bu & Li, 1987

#### Orangescirula

Taxon classificationAnimaliaTrombidiformesCunaxidae

Bu & Li, 1987

##### Historical review.

Bu and Li (1987a) erected Orangescirulinae and *Orangescirula* for a new species, *Orangescirula yongchuanensis*. Smiley (1992) described *Orangescirula kethleyi*. Corpuz-Raros (1996e) described *Orangescirula filipina*.

##### Diagnosis.

*Gnathosoma*. **Pedipalps** 5-segmented and reach beyond the subcapitulum by at most the distal half of the tibiotarsi. Basifemoral seta simple or spine-like. Telofemoral seta spine-like. Pedipalps end in a stout claw. **Subcapitulum** bears 6 pairs of setae: 2 pairs of adoral setae and 4 pairs of subcapitular setae (*hg_1_*_–_*_4_*). Setae *hg_1_* long and bent.

*Idiosoma, dorsal*. Proterosoma covered in a shield which bears 4 pairs of setae: 2 pairs of simple setae (*lps* and *mps*) and 2 pairs of setose sensilla (*at* and *pt*). Dorsal hysterosoma median plate present, fused to proterosomal shield; 1 to 5 pairs of dorsolateral plates present. Plates and shields smooth or reticulated. Seven pairs of setae present on the dorsal hysterosoma (*c_1_*–*f_1_*, *c_2_*, *h_2_*). Unsclerotized integument striated.

*Idiosoma, ventral*. **Coxae** I–II fused, coxae III–IV fused; coxae may coalesce medially for form a sternal shield. Each pair of coxae complemented with 3 pairs of setae. The genital plates each bear 4 setae; 2 pairs of genital papillae visible underneath the plates. 4–9 pairs of setae present on the integument between coxae II and the genital plates. Anal plates complemented with 2 pairs of setae (*ps_1-2_*). Two pairs of setae (*h_2_*, *pa*) located on the integument near the anal plates. Cupule *ih* present in close proximity to *h_2_*. **Legs** shorter than idiosoma; they are never constricted apically so as to end in lobes. Trichobothrium on leg tibia IV present. Ambulacral claws on either side of a four-rayed empodium present.

##### Key to adult female *Orangescirula*

(in part modified from Smiley 1992)

**Table:** 

1	Pedipalpal basifemora seta simple	*Orangescirula filipina*
–	Pedipalpal basifemora seta spine-like	2
2 (1)	Dorsal shields with large subcuticular reticulations; 2 pairs of dorsolateral plates present	*Orangescirula yongchuanensis*
–	Dorsal shield with extremely small subcuticular reticulations; 5 pairs of dorsolateral plates present	*Orangescirula kethleyi*

### New locality data

#### Scirula papillata

Taxon classificationAnimaliaTrombidiformesCunaxidae

Scirula papillata Lin, 1997: 169, Figs 1–6

##### Remarks.

The specimens examined represent the first report of *Scirula papillata* from the Western Hemisphere. The specimens examined correspond to Lin’s (1997) description except for telofemora I, which have 6 sts instead of 7 sts, and genua I, which have 9 setae (2 asl, 7 sts) instead of 8 setae.

**Material examined**(2 individuals on slides). 1 female adult (APGD 10-0424-008, #135719), ex deciduous leaf litter, USA, Arkansas, Washington Co, Devil’s Den State Park (35°46.817N, 94°14.750W), 24 April 2010, col. M. J. Skvarla ● 1 female adult (APGD 10-0826-003, # 135720), ex thick moss by creek near deciduous litter (maple, oak), USA, Pennsylvania, Somerset Co, Laurel Hill State Park, 1985’ elevation (40°00.963 N, 79°14.233 W), 26 August 2010, col. M. J. Skvarla.

#### Armascirus ozarkensis

Taxon classificationAnimaliaTrombidiformesCunaxidae

Armascirus ozarkensis Skvarla & Dowling, 2012: 6, Figs 2–4.

##### Remarks.

The specimens examined expand the range of this species within the Interior Highlands and are a new state record for Missouri.

##### Material examined

(2 individuals on slides). 1 adult female (APGD 11-1129-002), ex litter, USA, Arkansas, Bradley/Drew Co, Warren Prairie Natural Area, 21 June 2010, col. L. C. Thompson ● 1 adult female (APGD 10-0523-004), ex litter, USA, Missouri, Taney Co (36°41'11.98"N, 92°58'16.44"W), 23 May 2010, col. J. R. Fisher, D. M. Keeler.

#### Armascirus primigenius

Taxon classificationAnimaliaTrombidiformesCunaxidae

Armascirus primigenius Skvarla & Dowling, 2012: 13, Figs 8–10.

##### Remarks.

The specimens examined significantly expand the range of this species within the United States. The Ouachita specimens correspond to Skvarla and Dowling’s (2012) description except for genua IV, which have 1 asl, 5 sts instead of 1 asl, 4 sts.

##### Material examined

(3 individuals on slides). 1 adult female (APGD 13-0304-041, #131238), ex. Malaise trap in marsh, USA, Fairfax Co, George Washington Memorial Parkway, Dyke Marsh Wildlife Preserve, 11 April 2009, col. E. M. Barrows ● 2 adult females (APGD 12-0706-002, #135716), ex very dry oak.pine litter in small, rocky depression, USA, Arkansas, Polk Co, Ouachita National Forest, Black Fork Mountain Wilderness, Black Fork Trail (34°41.312'N, 94°18.691'W), 6 July 2012, col. M. J. Skvarla.

#### Dactyloscirus dolichosetosus

Taxon classificationAnimaliaTrombidiformesCunaxidae

Dactyloscirus dolichosetosus Den Heyer, 1979: 96, figs 71–77; Sepasgosarian 1984: 141; Smiley 1992: 223, Figs 117A, B; Castro 2008: 91; Skvarla and Dowling 2012: 30.

##### Remarks.

The specimens examined significantly expand the range of this species within the United States.

##### Material examined

(3 individuals on slides). 2 adult females (APGD 12-1020-012, #135721), ex. deciduous litter (maple, sweet gum, poison ivy) in disturbed area, USA, Virginia, Fairfax Co, George Washington Memorial Parkway, Dyke Marsh Wildlife Preserve (38°46'25"N, 77°03'06"W), 22 October 2012, col. A. P. G. Dowling ● 1 adult female (JRF 12-1028-010, #135722), ex. dry mixed litter with little tree cover in recently (~5 years) cut pine stand with shrubby oaks, USA, Arkansas, Montgomery Co, Ouachita National Forest (34°23'56"N, 93°51'22"W), 28 October 2010, col. J. R. Fisher, D. M. Keeler.

## Supplementary Material

XML Treatment for Cunaxidae

XML Treatment for Bonzinae

XML Treatment for Bonzia

XML Treatment for Parabonzia

XML Treatment for Cunaxoidinae

XML Treatment for Bunaxella

XML Treatment for Cunaxoides

XML Treatment for Denheyernaxoides

XML Treatment for Dunaxeus

XML Treatment for Funaxopsis

XML Treatment for Lupaeus

XML Treatment for Neocunaxoides

XML Treatment for Paracunaxoides

XML Treatment for Pulaeus

XML Treatment for Qunaxella

XML Treatment for Scutopalus

XML Treatment for Scirula

XML Treatment for Cunaxinae

XML Treatment for Allocunaxa

XML Treatment for Armascirus

XML Treatment for Cunaxa

XML Treatment for Cunaxatricha

XML Treatment for Dactyloscirus

XML Treatment for Riscus

XML Treatment for Rubroscirus

XML Treatment for Coleoscirinae

XML Treatment for Coleoscirus

XML Treatment for Neobonzia

XML Treatment for Neoscirula

XML Treatment for Pseudobonzia

XML Treatment for Scutascirus

XML Treatment for Orangescirula

XML Treatment for Scirula papillata

XML Treatment for Armascirus ozarkensis

XML Treatment for Armascirus primigenius

XML Treatment for Dactyloscirus dolichosetosus
